# Rapid *N*‑Trifluoromethylsulfinylation of Sulfoximines in Batch and Flow

**DOI:** 10.1021/acs.joc.6c00569

**Published:** 2026-06-04

**Authors:** Karthick Govindan, Pushbaraj Palani, Nian-Qi Chen, Meng-Yang Chang, Wei-Yu Lin

**Affiliations:** † Department of Medicinal and Applied Chemistry, 38023 Kaohsiung Medical University, Kaohsiung 80708, Taiwan; ‡ Department of Medical Research, Kaohsiung Medical University Hospital, Kaohsiung 80708, Taiwan; § Drug Development and Value Creation Research Centre, 38023 Kaohsiung Medical University, Kaohsiung 80708, Taiwan

## Abstract

Sulfoximines are privileged motifs in medicinal chemistry, and direct installation of the −SOCF_3_ group on these scaffolds remains challenging. Herein, we report an Et_3_N-catalyzed *N*-trifluoromethylsulfinylation of sulfoximines using the bench-stable reagent *N*-trifluoromethylsulfinylphthalimide under mild conditions. The process furnishes *N*-trifluoromethylsulfinylated sulfoximines in moderate to excellent yields and features a broad scope with high functional-group tolerance. Notably, phthalimide is formed as a valuable, readily recoverable coproduct, enabling a waste-minimized and sustainable process. Translation to a continuous-flow microreactor enables ultrafast reactivity with a residence time of only 6 s, delivering products in up to 86% yield. Mechanistic studies, gram-scale experiments, and structurally complex bioactive molecules further underscore the synthetic and practical utility of this transformation.

## Graphical abstract

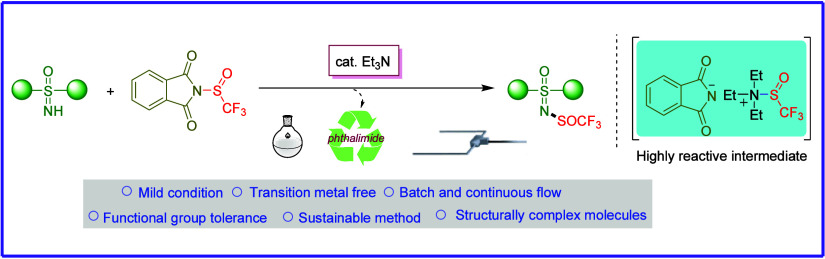

## Introduction

Sulfoximines, the monoaza congeners of sulfones, have emerged as valuable structural motifs in medicinal chemistry, agrochemical research, and asymmetric catalysis, owing to their architecturally rich *S­(VI)* framework and highly tunable stereoelectronic properties.^1^ The tetrahedral sulfur center within this versatile scaffold allows precise control over polarity, acidity, and 3D-orientation, while the nitrogen substituent provides an additional handle for modulating physicochemical behavior and improving biological performance.^2^ These features have supported their growing use in therapeutic agents, including antiasthmatics,^3^ HIV-1 protease inhibitors,^4^ antiproliferative molecules^5^ and pan-CDK inhibitors,^6^ and have established sulfoximines as important chiral auxiliaries in asymmetric synthesis.^7^ A parallel surge of interest has centered on the chemical union of sulfur–fluorine motifs; trifluoromethyl-derived groups across the *S­(II)*, *S­(IV)*, and *S­(VI)* oxidation states, have attracted significant attention.^8^ Advances in fluorinated building blocks and in understanding fluorine-mediated electronic effects have enabled diverse *N*-fluoroalkyl functionalizations.^9^ Within this context, *N*-SCF_3_ ^10^ and *N*-SO_2_CF_3_ ^11^ groups are well-developed, supported by reliable synthetic methods and demonstrated utility in molecular design. In contrast, the *N*-SOCF_3_ functionality remains less explored. The SOCF_3_ group combines strong electron-withdrawing character with high hydrophilicity—features that are highly desirable for tuning sulfoximine polarity and biological performance, yet no general method exists for installing this group onto the sulfoximine nitrogen.^12^ This gap represents a clear unmet need in the *N*-functionalization of sulfoximines (Scheme 1a).

**Scheme 1:**
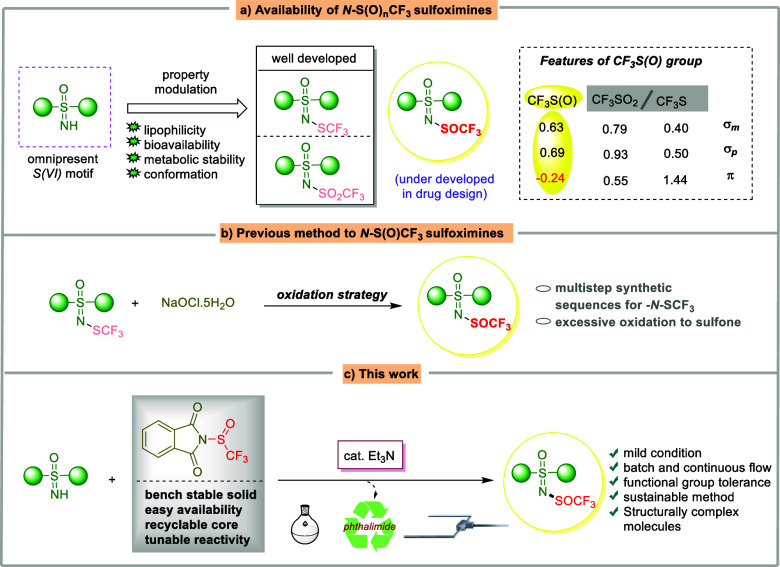
Background and Concept Overview

Existing strategies provide only limited access to *N*-SOCF_3_ sulfoximines.^13^ Oxidative upgrading of trifluoromethyl sulfides can generate sulfinyl-trifluoromethyl derivatives, but this method typically requires multistep synthesis of R-SCF_3_ precursors and often suffers from poor selectivity due to overoxidation (Scheme 1b).^14^ Moreover, the classical reagent CF_3_SOCl offers a direct route,^15^ but its toxicity, volatility, and instability severely restrict practicality, and only limited examples of *N*-functionalization have been reported. Although electrophilic sulfinyl-trifluoromethylating reagents have been introduced as safer alternatives,^16^ their applicability remains narrow. In a related context, phthalimide-derived acyl transfer reagents have recently emerged as efficient and practical electrophilic transfer reagents for acylation chemistry, highlighting the versatility and synthetic utility of imide-based transfer platforms.^17^

Motivated by these limitations and aligned with our research goal of developing metal-free, sustainable methods using bench-stable reagents for small molecules synthesis,^18^ we herein describe a mild and operationally straightforward electrophilic trifluoromethylsulfinylation protocol. This method employs *N*-(trifluoromethylsulfinyl)­phthalimide as a stable, recyclable, easily handled SOCF_3_ donor in the presence of a Lewis base catalyst. The approach provides rapid access to *N*-sulfinyl-trifluoromethylated sulfoximines in good yields. The transformation is rapidly adaptable to continuous-flow processes, exhibits broad functional group tolerance, and accommodates substrates derived from bioactive molecules (Scheme 1c).

## Results and Discussion

To assess the feasibility of our proposed strategy, we selected readily available phenyl sulfoximine (**1a**) as the model substrate and bench-stable *N*-(trifluoromethylsulfinyl)­phthalimide (**2a**) as the trifluoromethylsulfinylating reagent (Table 1) (see SI Tables S1–S4 for detailed optimization studies). Systematic evaluation of various reaction parameters identified optimal conditions consisting of **1a** (1.0 equiv), **2a** (1.5 equiv), and Et_3_N (20 mol %) in DMSO (0.1 M), stirred at room temperature for 1 h. Under these conditions, the desired product **3a** was obtained in 81% isolated yield (Table 1, entry 1). Replacing triethylamine with other bases, including DIPEA, DBU, or diethylamine, did not lead to any improvement in the formation of product **3a** (Table 1, entry 2). Increasing the reaction temperature to 80 °C also failed to enhance the yield relative to the standard conditions (Table 1, entry 3). Likewise, changing the solvent to 2-MeTHF, THF, 1,4-dioxane, or DMF resulted in no improvement in the yield of **3a** (Table 1, entry 4). Notably, either decreasing or increasing the loading of triethylamine led to diminished yields 76% and 75%, respectively (Table 1, entry 5). In addition, variation of the equivalents of reagent **2a** from the optimal amount, whether higher or lower, resulted in reduced yields of **3a** (Table 1, entry 6). Furthermore, shortening the reaction time to 0.5 h also led to a decreased yield (75%).

**Table 1:**
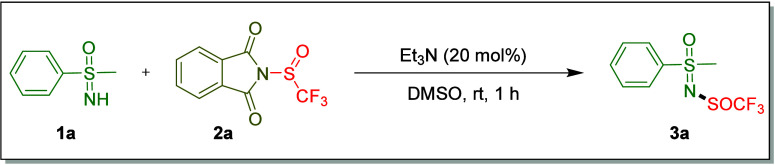
Reaction Optimization^a^,^b^

entry	variation	yield (%)^b^
1	none	81
2	DIPEA, DBU, Et_2_NH as a base	34/37/30
3	Et_3_N (80 °C)	27
4	2-MeTHF, THF, 1,4-dioxane, DMF as solvent	57/62/59/51
5	10.0, 50.0 mol % of Et_3_N instead of 20 mol %	76/75
6	1.2, 2.0 equiv of **2a** instead of 1.5 equiv.	63/59
7	0.5 h	75

a Reaction condition: **1a** (0.2 mmol), **2a** (1.5 equiv), Et_3_N (20 mol %) in DMSO (0.1 M) were stirred at rt for 1 h.

b Isolated yield.

With the optimized conditions established, we next evaluated the substrate scope of sulfoximines bearing diverse electronic properties. A broad range of electron-donating and electron-withdrawing substituents were compatible, delivering a series of *N*-trifluoromethylsulfinylated sulfoximines **3a**–**3j** in generally good yields (Scheme 2). Sulfoximines containing para electron-rich substituents, such as 4-Me and 4-OMe, performed efficiently to furnish products **3b** and **3c**. Notably, halogenated sulfoximines (−Cl and −Br) exhibited excellent compatibility, affording the corresponding products **3d** and **3e** in good yields. Electron-deficient sulfoximines also proceeded smoothly: substrates incorporating −COMe, −CN, and −NO_2_ substituents afforded the desired products **3f**–**3h** in 58–73% yields. The generality of this protocol was further demonstrated with alternative sulfoximine frameworks **2i** and **2j**, which readily underwent the transformation to give **3i** and **3j** in 71 and 57% yield, respectively. It is noteworthy that, in substrate containing potentially competing *N*-nucleophilic functional group **3j**, the reaction proceeded with excellent selectivity toward sulfoximine trifluoromethylsulfinylation without any observable side reactions at the additional nucleophilic sites. This selectivity likely arises from the higher nucleophilicity and favorable reactivity of the sulfoximine nitrogen under the optimized reaction conditions. In contrast, the bulky diphenyl substrate **2k** and the sulfonimidamide substrate **2l** failed to deliver the corresponding products, possibly due to steric hindrance.

**Scheme 2:**
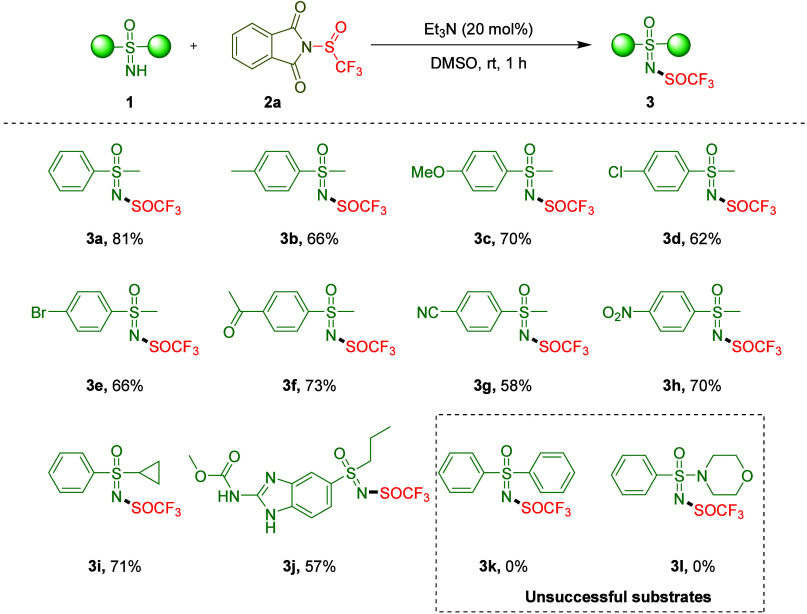
Substrate Scope of Sulfoximines^a^,^b^

a Reaction condition: **1** (0.2 mmol), **2a** (1.5 equiv), Et_3_N (20 mol %) and DMSO (0.1 M), rt, 1 h.
b Isolated yield.

To further demonstrate the practicality and broad applicability of this operationally simple protocol, we applied the optimized conditions to structurally elaborated sulfoximine derivatives containing pharmaceutically relevant motifs (Scheme 3i). Gratifyingly, sulfoximine derivatives of ibuprofen, an anti-inflammatory agent (**5a**), gemfibrozil, a lipid-lowering drug (**5b**), oxaprozin, an antirheumatic agent (**5c**), erucic acid, a naturally occurring omega-9-fatty acid (**5d**), and probenecid, an antigout drug (**5e**), were all amenable to Lewis base (Et_3_N) catalyzed trifluoromethylsulfinylation using a bench-stable reagent. Under the optimized conditions, the corresponding *N*-trifluoromethanesulfinylated sulfoximines **6a–6e** were obtained in good yields (58–78%).

**Scheme 3:**
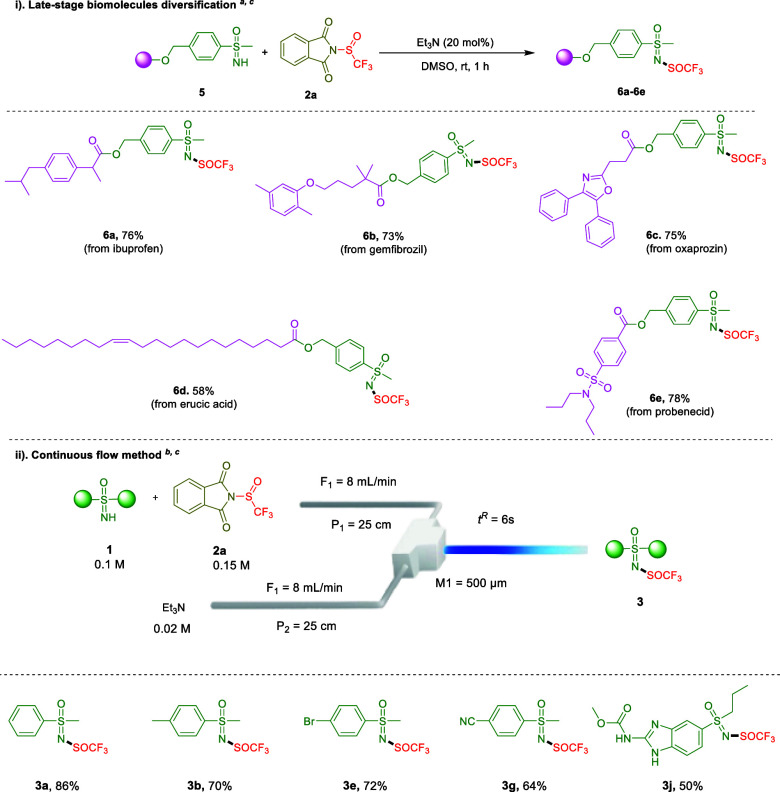
(i) Structurally Complex Molecules and (ii) Continuous-Flow Method

a (i) Reaction condition in batch: **1** (0.2 mmol), **2a** (1.5 equiv), Et_3_N (20 mol %) and DMSO (0.1 M), rt, 1 h.
b (ii) The reactions were performed under optimized continuous-flow conditions.
c Isolated yield.

Furthermore, we sought to translate the *N*-trifluoromethylsulfinylation protocol to a continuous-flow system. Although the batch protocol already proceeded efficiently, translation to a continuous-flow microreactor system was investigated to demonstrate the operational robustness, rapid reaction capability, and potential of the methodology. Owing to efficient mixing and enhanced mass- and heat-transfer characteristics,^19^ the transformation could be accomplished with an exceptionally short residence time at room temperature while maintaining good product yields. After comprehensive optimization of key reaction parameters, the optimal conditions were identified as a flow rate of 8 mL min^–1^ in DMSO with a residence time of 6 s. Under these conditions, products **3a**–**b**, **3e**, **3g**, and **3j** were obtained in 50–86% isolated yields (Scheme 3ii).

Furthermore, scalability is an important factor for the next stage of process development; herein, we tested using compounds **1a** and **1e** on a gram scale under the standard conditions. The reaction afforded the desired products, **3a** and **3e** in 70 and 62% yield, respectively (Scheme 4a). Notably, phthalimide **10**, a commercially valuable coproduct formed upon completion of the reaction, was recovered in 82% yield by simple filtration of the crude mixture. The recovered phthalimide **10** was subsequently converted to **2a** by reaction with trifluoromethylsulfinyl chloride under the same conditions, completing a two-step sequence that highlights the sustainability of this approach. Furthermore, the trifluoromethylsulfinyl group could be readily oxidized to the corresponding trifluoromethylsulfonyl group using *m*-CPBA at ambient temperature for 6 h, yielding product **7** in 96% yield (Scheme 4b (i)).^20^ The synthetic utility of the method was further demonstrated by subjecting **3e** to a Sonogashira coupling with a bromo-functionalized partner, delivering the coupled product **9** in good efficiency (Scheme 4b (ii)).^21^

**Scheme 4:**
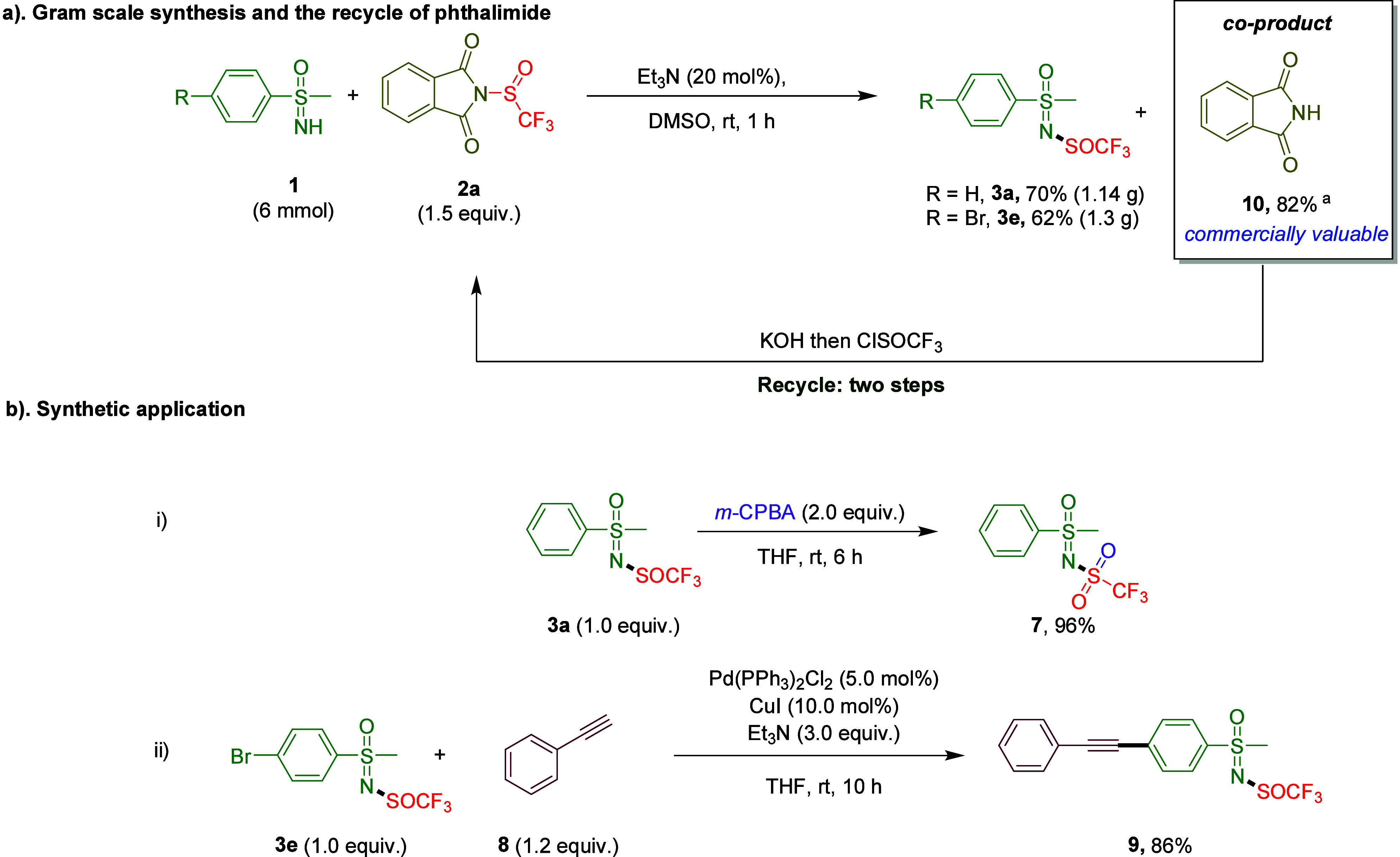
(a) Upscaling and Recycling (b) Post-Modifications

a The coproduct **10** was recovered only in the case of **3e**.

To gain further insight into the role of catalytic triethylamine and elucidate the underlying reaction mechanism, preliminary investigations were performed, and the results are summarized in Figure 1. We proposed that triethylamine initially interacts with reagent **2a**, forming an ion-pair adduct through nucleophilic displacement of the phthalimide moiety. This intermediate was expected to serve as the active species enabling subsequent trifluoromethylsulfinylation. To examine this hypothesis, a stoichiometric reaction between reagent **2a** and triethylamine was carried out. In contrast, mixing reagent **2a** (typically resonating at −85.5 ppm Figure 1A and product **3a** at −81.73 ppm Figure 1B) with 1.0 equiv of triethylamine led to the appearance of a new signal at −88.96 ppm after 1 h, coinciding with complete consumption of **2a** (Figure 1C).

**Figure 1:**
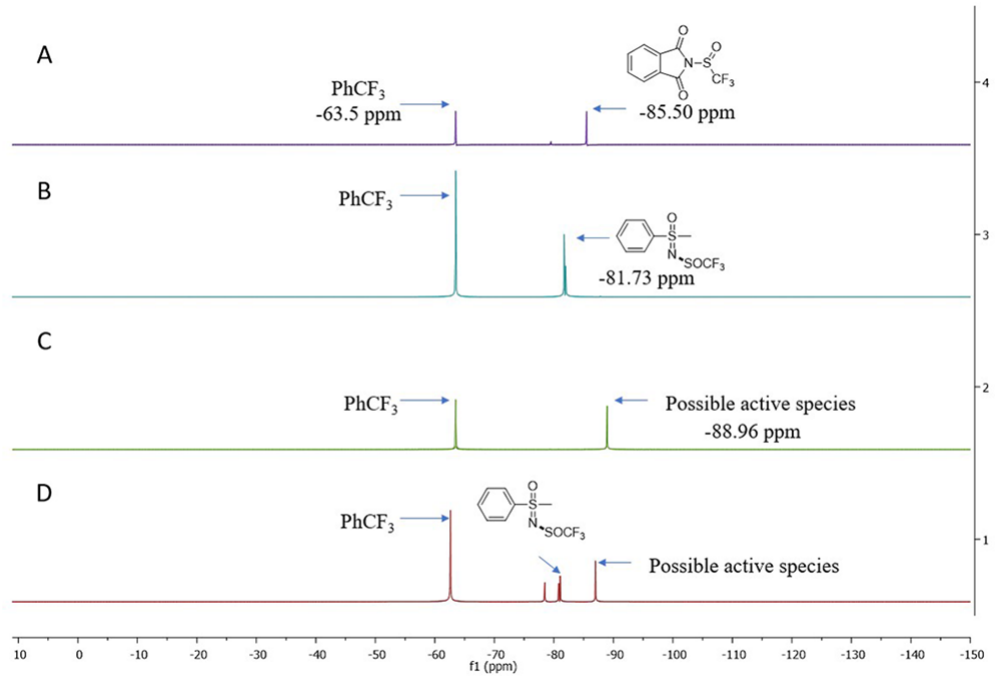
Et_3_N-catalyzed trifluoromethylsulfinylation of sulfoximines in THF, as determined by ^19^F NMR spectroscopy using PhCF_3_ as an internal standard: (A) reagent **2a**; (B) product **3a**; (C) Et_3_N and reagent **2a** (1:1) at RT for 1 h; (D) Et_3_N, reagent **1a**, **2a** (1:1:1), at RT for 1 h.

Subsequent addition of 1.0 equiv of imino­(methyl)­(phenyl)-λ^6^-sulfanone (**1a**) to the crude mixture afforded product **3a** in 28% yield. Notably, the newly formed species at −88.5 ppm was detected in 25% yield by ^19^F NMR (Figure 1D), indicating that it arises directly from the reaction of **2a** with triethylamine. Importantly, no detectable opening or cleavage of the internal N–C­(O) bond of the phthalimide framework was observed under the optimized conditions, indicating the stability and chemoselective behavior of the reagent during the transformation. Additional control experiments are provided in the Supporting Information (Scheme S1). Based on the mechanistic control studies described above, we propose a plausible mechanism for the formation of *N*-trifluoromethylsulfinyl sulfoximines, as illustrated in (Scheme 5). The reaction is initiated by the formation of an Et_3_N–S­(O)­CF_3_ adduct, generated through nucleophilic attack of triethylamine on *N*-(trifluoromethylsulfinyl)­phthalimide (**2a**). The resulting highly reactive intermediate **2a**’ then undergoes nucleophilic substitution by the sulfoximine substrate, furnishing the corresponding trifluoromethylsulfinylated sulfoximines as the final products, accompanied by phthalimide as a coproduct.

**Scheme 5:**
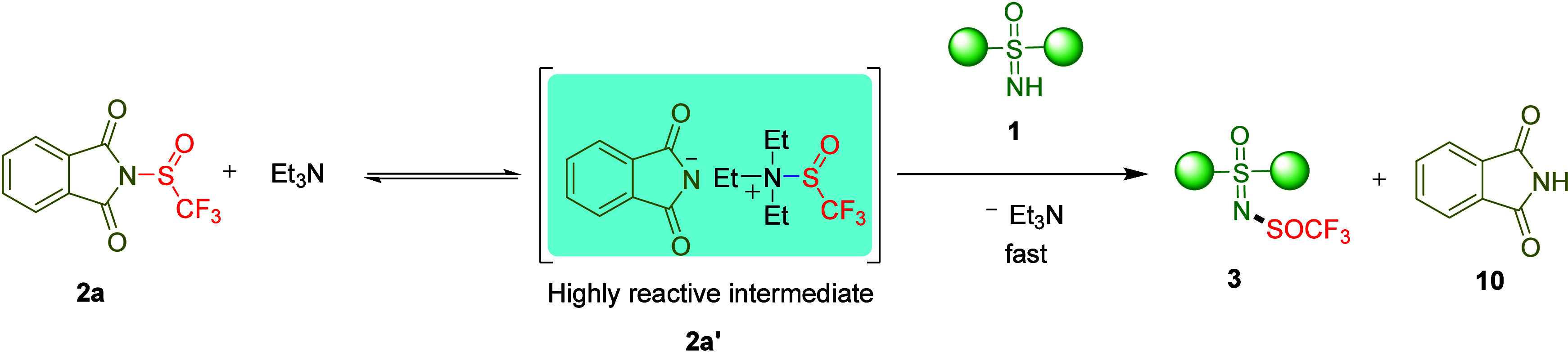
Plausible Mechanism

## Conclusion

In summary, an efficient Lewis base–catalyzed, transition-metal-free strategy for the synthesis of *N*-trifluoromethylsulfinyl sulfoximines has been developed using bench-stable *N*-(trifluoromethylsulfinyl)­phthalimide. The method exhibits broad substrate scope and excellent functional-group tolerance, and enables structurally complex molecules. Importantly, the transformation was translated to a continuous-flow microreactor, delivering up to 86% isolated yield with a residence time of only 6 s. Mechanistic studies implicate an ion-pair complex formed between the Lewis base and the reagent as the catalytically active species. This practical and scalable protocol provides rapid access to complex fluorine-containing sulfoximines of relevance to medicinal and process chemistry.

## Experimental Section

### General Information

Unless noted, all chemicals were purchased from commercial providers (Sigma-Aldrich, Alfa Aesar, TCI, Angene, ChemScene, and Matrix Scientific) and used directly without further purification, unless otherwise noted. Well-cleaned and oven-dried glassware was used for the experiments. Reaction was monitored by Thin Layer Chromatography (TLC), purchased as precoated with silica gel 60 F254 from Merck. Column chromatography was carried out using silica gel 230–400 mesh (purchased from Merck) with a mixture of ethyl acetate/hexane or hexane as the eluent. ^1^H NMR spectra were recorded on 400 MHz, and ^13^C NMR spectra were recorded on 100 MHz using a Varian mercury spectrometer using CDCl_3_ or DMSO-*d*_*6*_ as a solvent. The spectra were recorded and presented in chemical shifts (ppm) with tetramethylsilane (TMS) used as an internal standard. Multiplicities were provided in *s* (singlet), *d* (doublet), *t* (triplet), *q* (quartet), *bs* (broad singlet), m (multiplet), *dd* (doublet of doublet), *ddd* (doublet of doublet of doublet), and *ddt* (doublet of doublet of triplet). Coupling constants (*J*) were reported in Hz. All the compounds were characterized by high-resolution-ESI (electrospray) ionization were conducted on a JMS-T100LP AccuTOF LC-plus 4G mass spectrometer (JEOL, Tokyo, Japan) and HRMS (ESI+) on JMS-700 spectrometer. Melting points were determined using Fargo instruments.

### Experimental Procedure for *N*-trifluoromethylsulfinyl Sulfoximines (**3a–3j** and **6a–6e**)

An oven-dried 5 mL reaction tube was charged with (**1a**–**1j** and **5a**–**5e**) (0.2 mmol, 1.0 equiv), **2a** (0.3 mmol, 1.5 equiv), Et_3_N (20 mol %), DMSO (0.1 M, 2.0 mL). The resulting mixture was stirred at room temperature for about 1 h. After the completion of the reaction, reaction mixture was diluted with 10 mL of water. The aqueous layer was extracted with Ethyl acetate (3 × 10 mL), and the combined organic layer was washed with brine solution (1 × 5 mL). The final organic layer was then dried over MgSO_4_ and concentrated under reduced pressure to get the crude product. The obtained crude product was purified using column chromatography by eluting with ethyl acetate/hexane to afford desired product (**3a**–**3j** and **6a**–**6e**) up to 57–81% yields.

### Experimental Procedure for *N*-trifluoromethylsulfinyl Sulfoximines in the Continuous Flow Method (**3a**, **3b**, **3e**, **3g,** and **3j**)

A microreactor system consists of one T-shaped micromixer (M1), one microtube reaction (*R*_1_), two precooling units P_1_ (inner diameter Φ = 800 μm, length *L* = 25 cm), P_2_ (Φ = 800 μm, length *L* = 25 cm). A solution containing **1 and 2a** (0.1 M and 0.15 M in DMSO) (flow rate: *F*_1_ = 8 mL/min) and a solution of triethylamine (0.02 M in DMSO) (flow rate: *F*_2_ = 8 mL/min) were introduced into M1 (Φ = 500 μm) by syringe pumps. The resulting solution was passed through microtubing (*R*_1_ = 250 cm, Φ = 800 μm) with room temperature. After a steady state was reached, the solution was collected for 6 s in a vial. The resulting solution was extracted with ethyl acetate (10 mL × 3), and the organic layer was combined and washed with brine solution (5 mL). The organic layer was dried over anhydrous MgSO_4_, filtered, and concentrated under a vacuum. The crude product was purified by column chromatography (EtOAc/Hexane) and obtained **3a, 3b, 3e, 3g,** and **3j** in 50–86% yields.

#### 1,1,1-Trifluoro-*N*-(methyl­(oxo)­(phenyl)-*λ* ^6^-sulfaneylidene)­methanesulfinamide **(3a)**

The title compound was prepared according to the general experimental procedure on a 0.2 mmol for 1 h and the product was isolated by column chromatography (Ethyl acetate/Hexane) to afford a yellow oil (44 mg, 81%); trans:cis ratio 1:1; ^1^H NMR (CDCl_3_, 400 MHz) δ 8.07–8.02 (m, 1H), 8.01–7.95 (m, 1H), 7.78–7.71 (m, 1H), 7.68–7.61 (m, 2H), 3.39 (d, *J* = 18.4 Hz, 3H). ^13^C­{^1^H} NMR (CDCl_3_, 101 MHz) δ 138.3, 137.8, 135.2, 135.0, 130.2, 130.1, 128.3, 127.5, 47.4, 47.1. ^19^F­{^1^H} NMR (CDCl_3_, 376 MHz) δ −79.95 (s), −80.30 (s) (3F); HRMS (ESI) calculated for C_8_H_8_NO_2_F_3_NaS_2_ [M + Na]; 293.9841found 293.9837.

#### 1,1,1-Trifluoro-*N*-(methyl­(oxo)­(p-tolyl)- *λ* ^6^-sulfaneylidene)­methanesulfinamide **(3b)**

The title compound was prepared according to the general experimental procedure on a 0.2 mmol for 1 h and the product was isolated by column chromatography (Ethyl acetate/Hexane) to afford a yellow oil (37.7 mg, 66%); trans:cis ratio 1:1; ^1^H NMR (CDCl_3_, 400 MHz) δ 7.89 (ddt, *J* = 25.1, 8.8, 2.2 Hz, 2H), 7.44 (ddd, *J* = 8.8, 4.7, 0.7 Hz, 2H), 3.37 (d, *J* = 17.2 Hz, 3H), 2.48 (s, 3H). ^13^C­{^1^H} NMR (CDCl_3_, 101 MHz) δ 146.5, 146.3, 135.1, 134.4, 130.7, 130.5, 128.2, 127.4, 125.6, 125.5, 122.3, 122.2, 47.3, 47.0, 21.7, 21.6. ^19^F­{^1^H} NMR (CDCl_3_, 376 MHz) δ −79.98 (s), −80.35 (s) (3F); HRMS (ESI) calculated for C_9_H_10_NO_2_F_3_NaS_2_ [M + Na]; 307.9997 found 307.9994.

#### 1,1,1-Trifluoro-*N*-((4-methoxyphenyl)­(methyl)­(oxo)-λ^6^-sulfaneylidene)­methanesulfinamide **(3c)**

The title compound was prepared according to the general experimental procedure on a 0.2 mmol for 1 h and the product was isolated by column chromatography (Ethyl acetate/Hexane) to afford a yellow oil (42 mg, 70%); trans:cis ratio 1.1:1; ^1^H NMR (CDCl_3_, 400 MHz) δ 8.00–7.85 (m, 2H), 7.13–7.02 (m, 2H), 3.90 (s, 3H), 3.36 (d, *J* = 17.4 Hz, 3H). ^13^C­{^1^H} NMR (CDCl_3_, 101 MHz) δ 165.0, 164.9, 130.69, 129.9, 129.1, 128.3, 125.7, 125.6, 122.4, 122.3, 115.4, 115.2, 56.01, 47.6, 47.4. ^19^F­{^1^H} NMR (CDCl_3_, 376 MHz) δ −79.98 (s), −80.36 (s) (3F); HRMS (ESI) calculated for C_9_H_10_NO_3_F_3_NaS_2_ [M + Na]; 323.9946 found 323.9941.

#### *N*-((4-Chlorophenyl)­(methyl)­(oxo)-*λ* ^6^-sulfaneylidene)-1,1,1-trifluoromethanesulfinamide**(3d)**

The title compound was prepared according to the general experimental procedure on a 0.2 mmol for 1 h and the product was isolated by column chromatography (Ethyl acetate/Hexane) to afford a yellow oil (40.3 mg, 62%); ^1^H NMR (CDCl_3_, 400 MHz) δ 7.96–7.92 (m, 2H), 7.67–7.60 (m, 2H), 3.43 (s, 3H). ^13^C­{^1^H} NMR (CDCl_3_, 101 MHz) δ 142.0, 136.8, 130.5, 130.4, 129.8, 129.0, 125.7, 122.3, 47.5, 47.2. ^19^F­{^1^H} NMR (CDCl_3_, 376 MHz) δ −79.88 (s) (3F); HRMS (ESI) calculated for C_8_H_7_NO_2_F_3_NaS_2_Cl [M + Na]; 327.9451 found 327.9444.

#### *N*-((4-Bromophenyl)­(methyl)­(oxo)-*λ* ^6^-sulfaneylidene)-1,1,1-trifluoromethanesulfinamide **(3e)**

The title compound was prepared according to the general experimental procedure 4 on a 0.2 mmol for 1 h and the product was isolated by column chromatography (Ethyl acetate/Hexane) to afford a white crystalline powder (39 mg, 66%); mp 116–118; ^1^H NMR (CDCl_3_, 400 MHz) δ 7.69–7.64 (m, 2H), 7.54–7.49 (m, 2H), 2.72–2.69 (m, 3H). ^13^C­{^1^H} NMR (CDCl_3_, 101 MHz) δ 144.9, 132.7, 125.6, 125.2, 44.1. ^19^F­{^1^H} NMR (CDCl_3_, 376 MHz) δ −79.96 (s) (3F); HRMS (ESI) calculated for C_8_H_7_NO_2_F_3_NaS_2_Br [M + Na]; 371.8946 found 371.8954.

#### *N*-((4-Acetylphenyl)­(methyl)­(oxo)-λ^6^-sulfaneylidene)-1,1,1-trifluoromethanesulfinamide **(3f)**

The title compound was prepared according to the general experimental procedure 4 on a 0.2 mmol for 1 h and the product was isolated by column chromatography (Ethyl acetate/Hexane) to afford a white powder (45.7 mg, 73%); trans:cis ratio 1.2:1; mp 98–102; ^1^H NMR (CDCl_3_, 400 MHz) δ 8.18 (dd, *J* = 7.2, 1.6 Hz, 3H), 8.12–8.08 (m, 1H), 3.42 (d, *J* = 20.0 Hz, 3H), 2.67 (dd, *J* = 2.8, 0.8 Hz, 3H). ^13^C­{^1^H} NMR (CDCl_3_, 101 MHz) δ 196.4, 196.3, 142.2, 1141.9, 141.9, 141.8, 129.7, 129.6, 128.7, 128.0, 125.6, 125.6, 122.3, 122.3, 47.3, 46.9, 27.1, 27.0. ^19^F­{^1^H} NMR (CDCl_3_, 376 MHz) δ −80.00 (s), −80.18 (s) (3F); HRMS (ESI) calculated for C_10_H_10_NO_3_F_3_NaS_2_ [M + Na]; 335.9946 found 335.9946.

#### *N*-((4-Cyanophenyl)­(methyl)­(oxo)-*λ* ^6^-sulfaneylidene)-1,1,1-trifluoromethanesulfinamide **(3g)**

The title compound was prepared according to the general experimental procedure on a 0.2 mmol for 1 h and the product was isolated by column chromatography (Ethyl acetate/Hexane) to afford a yellow oil (34.4 mg, 58%); ^1^H NMR (CDCl_3_, 400 MHz) δ 8.17–8.11 (m, 2H), 7.99–8.11 (dt, *J* = 8.5, 1.8 Hz, 2H), 3.47 (s, 3H). ^13^C­{^1^H} NMR (CDCl_3_, 101 MHz) δ 142.7, 133.7, 129.0, 128.3, 125.6, 122.3, 118.8, 116.7, 47.2. ^19^F­{^1^H} NMR (CDCl_3_, 376 MHz) δ −79.75 (s) (3F); HRMS (ESI) calculated for C_9_H_7_N_2_O_2_F_3_NaS_2_ [M + Na]; 318.9793 found 318.9801.

#### 1,1,1-Trifluoro-*N*-(methyl­(4-nitrophenyl)­(oxo)-*λ* ^6^ *-*sulfaneylidene)­methanesulfinamide **(3h)**

The title compound was prepared according to the general experimental procedure on a 0.2 mmol for 1 h and the product was isolated by column chromatography (Ethyl acetate/Hexane) to afford a yellow oil (44.3 mg, 70%); ^1^H NMR (CDCl_3_, 400 MHz) δ 8.52–8.47 (m, 2H), 8.26–8.20 (m, 2H), 3.50 (s, 3H). ^13^C­{^1^H} NMR (CDCl_3_, 101 MHz) δ 151.4, 144.1, 129.0, 125.5, 125.1, 122.2, 47.3. ^19^F­{^1^H} NMR (CDCl_3_, 376 MHz) δ −79.72 (s) (3F); HRMS (ESI) calculated for C_8_H_7_N_2_O_4_F_3_NaS_2_ [M + Na]; 338.9692 found 338.9693.

#### *N*-(Cyclopropyl­(oxo)­(phenyl)-*λ* ^6^-sulfaneylidene)-1,1,1-trifluoromethanesulfinamide **(3i)**

The title compound was prepared according to the general experimental procedure on a 0.2 mmol for 1 h and the product was isolated by column chromatography (Ethyl acetate/Hexane) to afford colorless liquid (42.2 mg, 71%); ^1^H NMR (CDCl_3_, 400 MHz) δ 7.97 (m, 2H), 7.74–7.68 (m, 1H), 7.65–7.58 (m, 2H), 2.76 (m, 1H), 1.67–1.55 (m, 1H), 1.46–1.20 (m, 2H), 1.16–1.05 (m, 1H). ^13^C­{^1^H} NMR (CDCl_3_, 101 MHz) δ 134.6, 134.5, 129.9, 129.7, 128.1, 127.5, 35.2, 35.1, 7.1, 7.1, 6.7, 6.1. ^19^F­{^1^H} NMR (CDCl_3_, 376 MHz) δ −80.29 (s), −80.50 (s) (3F); HRMS (ESI) calculated for C_10_H_10_NO_2_F_3_NaS_2_ [M + Na]; 319.9997 found 319.9989.

#### Methyl-(5-(*N*-((trifluoromethyl)­sulfinyl)­propylsulfonimidoyl)-1*H*-benzo­[*d*]­imidazol-2-yl)­carbamate **(3j)**

The title compound was prepared according to the general experimental procedure on a 0.2 mmol for 1 h and the product was isolated by column chromatography (Ethyl acetate/Hexane) to afford colorless liquid (47 mg, 57%); ^1^H NMR (DMSO, 400 MHz) δ 12.46 (s, 1H), 11.70 (s, 1H), 8.02 (d, *J* = 22.4 Hz, 1H), 7.72–7.63 (m, 2H), 3.80 (s, 3H), 1.75–1.50 (m, 2H), 0.93 (td, *J* = 7.5, 1.1 Hz, 3H). ^13^C­{^1^H} NMR (DMSO, 101 MHz) δ 154.6, 126.2, 126.2, 122.9, 122.9, 58.8, 53.3, 16.3, 16.3, 12.5, 12.5. ^19^F­{^1^H} NMR (DMSO, 376 MHz) δ −79.35 (s), −79.87 (s) (3F); HRMS (ESI) calculated for C_13_H_15_N_4_O_4_F_3_NaS_2_ [M + Na]; 435.0379 found 435.0381.

#### 4-(*S*-Methyl-*N*-((trifluoromethyl)­sulfinyl)­sulfonimidoyl)­benzyl-2-(4-isobutylphenyl) propanoate **(6a)**

The title compound was prepared according to the general experimental procedure on a 0.2 mmol for 1 h and the product was isolated by column chromatography (Ethyl acetate/Hexane) to afford colorless liquid (74.5 mg, 76%); ^1^H NMR (CDCl_3_, 400 MHz) δ 8.29 (d, *J* = 8.4 Hz, 1H), 8.22 (d, *J* = 8.4 Hz, 1H), 7.79–7.71 (m, 2H), 7.54 (d, *J* = 6.8 Hz, 2H), 7.45 (d, *J* = 8.0 Hz, 2H), 5.64–5.44 (m, 2H), 4.13 (q, *J* = 7.2 Hz, 1H), 3.70 (d, *J* = 18.0 Hz, 3H), 2.80 (d, *J* = 7.2 Hz, 2H), 2.23–2.16 (m, 1H), 1.87 (d, *J* = 7.2 Hz, 3H), 1.24 (d, *J* = 6.8 Hz, 6H). ^13^C­{^1^H} NMR (CDCl_3_, 101 MHz) δ 174.51, 144.5, 144.3, 141.3, 137.5, 129.8, 128.8, 128.7, 128.6, 128.0, 127.6, 65.0, 47.7, 47.4, 45.4, 45.3, 30.5, 22.7, 18.5. ^19^F­{^1^H} NMR (CDCl_3_, 376 MHz) δ −79.68 (s), −80.00 (s) (3F); HRMS (ESI) calculated for C_22_H_26_NO_4_F_3_NaS_2_ [M + Na]; 512.1148 found 512.1141.

#### 4-(*S*-Methyl-*N*-((trifluoromethyl)­sulfinyl)­sulfonimidoyl)­benzyl-4-(2,5-dimethylphenoxy)-2,2-dimethylbutanoate **(6b)**

The title compound was prepared according to the general experimental procedure on a 0.2 mmol for 1 h and the product was isolated by column chromatography (Ethyl acetate/Hexane) to afford colorless liquid (76 mg, 73%); ^1^H NMR (CDCl_3_, 400 MHz) δ 8.42 (d, *J* = 8.4 Hz, 1H), 8.36 (d, *J* = 8.4 Hz, 1H), 8.02–7.96 (m, 2H), 7.38 (d, *J* = 7.5 Hz, 1H), 7.04 (d, *J* = 7.5 Hz, 1H), 6.99 (s, 1H), 5.59 (s, 2H), 4.30 (s, 2H), 3.76 (d, *J* = 20.0 Hz, 3H), 2.69 (s, 3H), 2.54 (s, 3H), 2.16 (dd, *J* = 7.2, 4.8 Hz, 4H), 1.67 (s, 6H). ^13^C­{^1^H} NMR (CDCl_3_, 101 MHz) δ 177.6, 157.2, 144.6, 144.4, 138.1, 137.6, 136.9, 130.7, 129.0, 128.9, 128.1, 123.8, 121.2, 112.4, 68.1, 65.0, 47.6, 47.3, 42.6, 37.4, 25.5, 21.7, 16.1. ^19^F­{^1^H} NMR (CDCl_3_, 376 MHz) δ −79.67 (s), −79.98 (s) (3F); HRMS (ESI) calculated for C_24_H_30_NO_5_F_3_NaS_2_ [M + Na]; 556.1415 found 556.1409.

#### 4-(*S*-Methyl-*N*-((trifluoromethyl)­sulfinyl)­sulfonimidoyl)­benzyl-3-(4,5-diphenyloxazol-2-yl)­propanoate **(6c)**

The title compound was prepared according to the general experimental procedure on a 0.2 mmol for 1 h and the product was isolated by column chromatography (Ethyl acetate/Hexane) to afford colorless liquid (86.5 mg, 75%); ^1^H NMR (CDCl_3_, 400 MHz) δ 7.90–7.86 (m, 1H), 7.82–7.77 (m, 1H), 7.63–7.52 (m, 6H), 7.39–7.30 (m, 6H), 5.26 (s, 2H), 3.29 (d, *J* = 16.4 Hz, 3H), 3.21 (t, *J* = 7.1 Hz, 2H), 3.02 (t, *J* = 7.2 Hz, 2H). ^13^C­{^1^H} NMR (CDCl_3_, 101 MHz) δ 171.6, 171.5, 161.4, 145.5, 143.6, 143.5, 137.6, 135.0, 132.3, 128.7, 128.6, 128.5, 128.4, 128.3, 128.2, 127.8, 127.6, 126.4, 126.3, 64.9, 64.8, 47.2, 46.9, 30.8, 23.3. ^19^F­{^1^H} NMR (CDCl_3_, 376 MHz) δ −79.91 (s), −80.22 (s) (3F); HRMS (ESI) calculated for C_27_H_23_N_2_O_5_F_3_NaS_2_ [M + Na]; 599.0893 found 599.0888.

#### 4-(*S*-Methyl-*N*-((trifluoromethyl)­sulfinyl)­sulfonimidoyl)­benzyl-(*Z*)-docos-13-enoate **(6d)**

The title compound was prepared according to the general experimental procedure on a 0.2 mmol for 1 h and the product was isolated by column chromatography (Ethyl acetate/Hexane) to afford colorless liquid (72.2 mg, 58%); ^1^H NMR (CDCl_3_, 400 MHz) δ 8.05–8.01 (m, 1H), 7.99–7.95 (m, 1H), 7.60 (dd, *J* = 8.5, 3.0 Hz, 2H), 5.37–5.28 (m, 2H), 5.20 (s, 2H), 3.38 (d, *J* = 19.7 Hz, 3H), 2.38 (t, *J* = 7.6 Hz, 2H), 1.99 (dd, *J* = 12.4, 6.7 Hz, 4H), 1.69–1.59 (m, 2H), 1.37–1.16 (m, 28H), 0.86 (t, *J* = 6.9 Hz, 3H). ^13^C­{^1^H} NMR (CDCl_3_, 101 MHz) δ 172.2, 143.1, 142.9, 128.8, 127.5, 126.7, 63.4, 46.3, 46.1, 33.1, 30.8, 28.7, 28.6, 28.5, 28.4, 28.2, 28.1, 26.1, 23.8, 21.6, 13.0. ^19^F­{^1^H} NMR (CDCl_3_, 376 MHz) δ −79.95 (s), −80.26 (s) (3F); HRMS (ESI) calculated for C_31_H_50_NO_4_F_3_NaS_2_ [M + Na]; 644.3026 found 644.3019.

#### 4-(*S*-Methyl-*N*-((trifluoromethyl)­sulfinyl)­sulfonimidoyl)­benzyl-4-(*N*,*N*-dipropylsulfamoyl)­benzoate **(6e)**

The title compound was prepared according to the general experimental procedure on a 0.2 mmol for 1 h and the product was isolated by column chromatography (Ethyl acetate/Hexane) to afford colorless liquid (89 mg, 78%); ^1^H NMR (CDCl_3_, 400 MHz) 1H NMR (400 MHz,) δ 8.20–8.16 (m, 2H), 8.09–7.99 (m, 2H), 7.90–7.86 (m, 2H), 7.73–7.68 (m, 2H), 5.48 (s, 2H), 3.39 (d, J = 19.6 Hz, 3H), 3.13–3.05 (m, 4H), 1.53 (dq, *J* = 14.9, 7.4 Hz, 4H), 0.87–0.82 (m, 6H). ^13^C­{^1^H} NMR (CDCl_3_, 101 MHz) δ 163.8, 144.0, 143.9, 142.4, 142.2, 137.3, 136.9, 131.7, 129.4, 128.3, 128.1, 127.7, 126.9, 126.2, 64.7, 49.0, 46.4, 46.1, 21.0, 10.2. ^19^F­{^1^H} NMR (CDCl_3_, 376 MHz) δ −79.91, −80.23 (3F); HRMS (ESI) calculated for C_22_H_27_N_2_O_6_F_3_NaS_3_ [M + Na]; 591.0876 found 591.0869.

#### 1,1,1-Trifluoro-*N*-(methyl­(oxo)­(phenyl)-*λ* ^6^-sulfaneylidene)­methanesulfonamide **(7)**

An oven-dried 20 mL reaction tube equipped with a magnetic stir bar was charged with compound **3a** (1.0 mmol, 1.0 equiv), meta-chloroperbenzoic acid (*m*-CPBA, 2.0 equiv), and tetrahydrofuran (THF, 6.0 mL). The reaction mixture was stirred at room temperature for approximately 6 h. Upon completion, water (30 mL) was added, and the mixture was extracted with ethyl acetate (3 × 30 mL). The combined organic extracts were dried over anhydrous MgSO_4_, filtered, and concentrated under reduced pressure. The resulting residue was purified by silica gel column chromatography using a mixture of ethyl acetate and hexane as the eluent to afford the oxidation product **7** as a pale-yellow oil (276 mg, 96% yield). ^1^H (CDCl_3_, 400 MHz) δ 8.04–7.99 (m, 2H), 7.80–7.75 (m, 1H), 7.70–7.64 (m, 2H), 3.50 (s, 3H). ^13^C­{^1^H} NMR (CDCl_3_, 101 MHz) δ 137.1, 135.4, 130.1, 127.2, 120.8, 117.6, 46.7. ^19^F­{^1^H} NMR (CDCl_3_, 376 MHz) δ −78.63 (3F); HRMS (ESI) calculated for C_8_H_8_NO_3_F_3_NaS_2_ [M + Na]; 309.9790 found 309.97984.

#### 1,1,1-Trifluoro-*N*-(methyl­(oxo)­(4-(phenylethynyl)­phenyl)-*λ* ^6^-sulfaneylidene)­methanesulfinamide **(9)**

A mixture of **3e** (0.5 mmol), CuI (0.05 mmol, 10 mol %), Pd­(PPh_3_)_2_Cl_2_ (0.025 mmol, 5 mol %), and Et_3_N (5 mL) was sequentially added under a nitrogen atmosphere. Phenylacetylene (0.65 mmol, 1.3 equiv) was introduced via syringe, and the reaction mixture was stirred at room temperature for 10 h, with progress monitored by TLC. Upon completion, the mixture was diluted with water (10 mL) and extracted with ethyl acetate (3 × 10 mL). The combined organic layers were washed with water and brine, dried over MgSO_4_, and filtered. Removal of the solvent under reduced pressure afforded a crude residue, which was purified by silica gel column chromatography to give compound **9** in (160 mg, 86% yield). mp. 130–133 °C; ^1^H (CDCl_3_, 400 MHz) δ 7.96 (dt, *J* = 9.2, 2.2 Hz, 2H), 7.75 (dt, *J* = 8.8, 2.2 Hz, 2H), 7.58–7.52 (m, 2H), 7.42–7.34 (m, 3H), 3.43 (s, 3H). ^13^C­{^1^H} NMR (CDCl_3_, 101 MHz) δ 137.0, 132.7, 131.9, 130.6, 129.4, 128.6, 127.4, 121.9, 94.7, 87.1, 47.3. ^19^F­{^1^H} NMR (CDCl_3_, 376 MHz) δ −79.89 (3F); HRMS (ESI) calculated for C_16_H_12_NO_2_F_3_NaS_2_ [M + Na]; 394.0154 found 394.0159.

## Supplementary Material



## Data Availability

The data underlying this study are available in the published article and its Supporting Information.

## Notes

General reaction procedures, detailed optimization, and NMR spectra (PDF)
