# Supplementary material for: Rapid N‑Trifluoromethylsulfinylation of Sulfoximines in Batch and Flow
Source: J Org Chem. 2026 Jun 4;91(24):8280–8. doi: 10.1021/acs.joc.6c00569 (PMC13288634; doi:10.1021/acs.joc.6c00569)

Supporting Information

for

Rapid *N*-Trifluoromethylsulfinylation of Sulfoximines in Batch and Flow

Karthick Govindan, [a] ‡ Pushbaraj Palani, [a] ‡ Nian-Qi Chen, [a] ‡ Meng-Yang Chang [a] and Wei-Yu Lin [a-c] *

[a] Department of Medicinal and Applied Chemistry, Kaohsiung Medical University, Kaohsiung 80708, Taiwan.

[b] Department of Medical Research, Kaohsiung Medical University Hospital, Kaohsiung 80708, Taiwan.

[c] Drug Development and Value Creation Research Centre, Kaohsiung Medical University, Kaohsiung 80708, Taiwan.

*Corresponding author: Wei-Yu Lin, wylin@kmu.edu.tw

‡ Equally contributed

Table of Contents

1	Optimization of reaction parameters	S3-S5
	Table S1. Optimization of base	S3
	Table S2. Optimization of temperature and atmosphere	S3-S4
	Table S3. Optimization of solvent	S4
	Table S4. Optimization of base mol% and reaction time	S4-S5
2	Synthesis of starting materials	S5-S7
	2.1 Synthesis of potassium phthalimide	S5
	2.2 Synthesis of 2-((trifluoromethyl)sulfinyl) isoindoline-1,3-dione (**2a**)	S5
	2.3 Synthesis of *NH*-sulfoximines (**1a-1j**)	S6
	2.4 Synthesis of biomolecule coupled *NH*-sulfoximines (**5a-5e**)	S6-S7
	2.5 Characterization data of starting materials (**5b-5d**)	S7-S8
3	Experimental procedure for *N*-trifluoromethylsulfonyl sulfoximines (**3a-3j** and **6a-6e**)	S8-S9
4	General procedure for the integrated continuous flow approach	S9-S10
	4.1 Table S5. Optimization conditions of continuous flow method	S9
	4.2 Experimental procedure for *N*-trifluoromethylsulfonyl sulfoximines in continuous flow method (**3a, 3b, 3e, 3g & 3j**)	S10
5	Experimental procedure for gram scale synthesis	S10-S11
6	Control studies	S11-S12
	6.1 Experimental procedure for radical trapping experiments	S11
	6.2 Experimental procedure for study of triethylamine role in the reaction	S11-S12
	6.3 Result and discussion of control studies	S12
7	Mechanistic studies	S13-S16
8	References	S17
9	Copies of ^{1}H, ^{13}C and ^{19}F	S18-S74

1. Optimization of reaction parameters

Table S1. Optimization of base

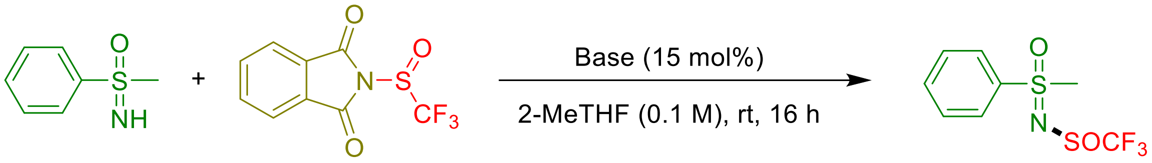

S. No.	1a (equiv.)	2a (equiv.)	Base	Solvent	Time (h)	Yield (%) [a]
1.	1.0	1.2	Et_3N	2-MeTHF	16	57
2.	1.2	1.0	Et_3N	2-MeTHF	16	38
3.	1.0	1.2	-	2-MeTHF	16	20
4.	1.0	1.2	DIPEA	2-MeTHF	16	34
5.	1.0	1.2	DBU	2-MeTHF	16	37
6.	1.0	1.2	DMAP	2-MeTHF	16	48
7.	1.0	1.2	Et_2NH	2-MeTHF	16	20
8.	1.0	1.2	DABCO	2-MeTHF	16	34
9.	1.0	1.2	N-Butylamine	2-MeTHF	16	40
10.	1.0	1.2	Na_2CO_3	DMSO	16	41
11.	1.0	1.2	K_2CO_3	DMSO	16	39
12.	1.0	1.2	Cs_2CO_3	DMSO	16	31

Table S2. Optimization of temperature and atmosphere

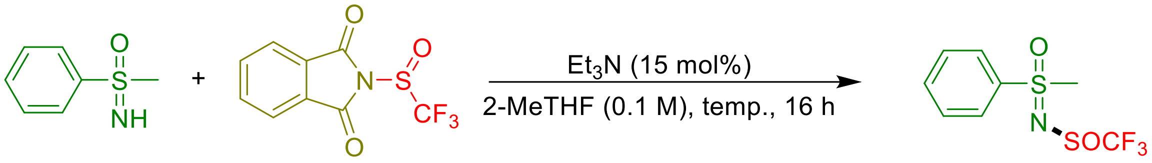

S. No.	1a (equiv.)	2a (equiv.)	Base	Solvent	Time (h)	Yield (%) [a]
1.	1.0	1.2	Et_3N (40 ^{o}C)	2-MeTHF	16	25
2.	1.0	1.2	Et_3N (60 ^{o}C)	2-MeTHF	16	28

3.	1.0	1.2	Et_3N (80 °C)	2-MeTHF	16	27
4.	1.0	1.2	Et_3N (N_2)	2-MeTHF	16	52
5.	1.0	1.2	Et_3N (O_2)	2-MeTHF	16	30

Table S3. Optimization of solvent

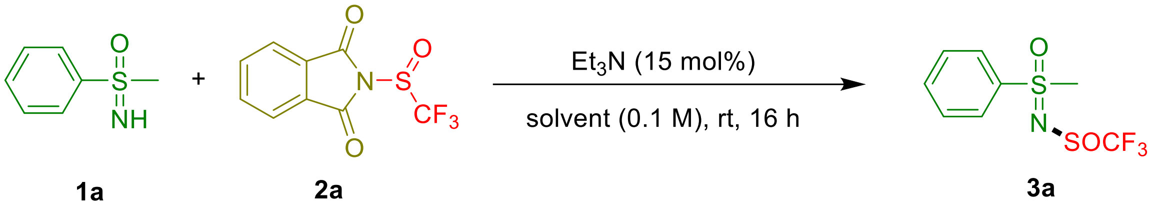

S. No.	1a (equiv.)	2a (equiv.)	Base	Solvent	Time (h)	Yield (%) [a]
1.	1.0	1.2	Et_3N	THF	5	60
2.	1.0	1.2	Et_3N	Acetonitrile	3	29
3.	1.0	1.2	Et_3N	DMSO	3	63
4.	1.0	1.2	Et_3N	DCE	3	17
5.	1.0	1.2	Et_3N	1,4-Dioxane	3	59
6.	1.0	1.2	Et_3N	DMF	3	51
7.	1.0	1.2	Et_3N	MeOH	3	48

Table S4. Optimization of mol% of base and time

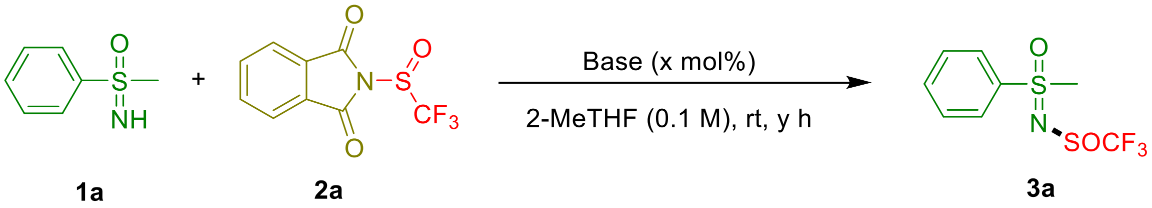

S. No.	1a (equiv.)	2a (equiv.)	Base (x mol%)	Solvent	Time (h)	Yield (%) [a]
1.	1.0	1.5	Et_3N	DMSO	3	73
2.	1.0	2.0	Et_3N	DMSO	3	59
3.	1.0	1.5	Et_3N (10)	DMSO	3	71
4.	1.0	1.5	Et_3N (20)	DMSO	3	80

5.	1.0	1.5	Et_3N (50)	DMSO	3	76
**6.**	**1.0**	**1.5**	**Et_3N**	**DMSO**	**1**	**81**
7.	1.0	1.5	Et_3N	DMSO	0.5	75

2. Synthesis of starting materials

2.1 Synthesis of potassium phthalimide

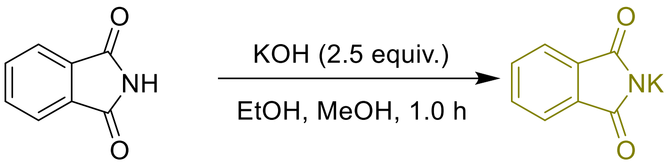

Following a modified literature procedure, [1] an oven-dried 100 mL round-bottom flask equipped with a magnetic stir bar was charged with isoindoline-1,3-dione (1.47 g, 10 mmol, 1.0 equiv.) dissolved in methanol (5 mL). A solution of potassium hydroxide (1.40 g, 25 mmol, 2.5 equiv.) in ethanol (10 mL) was then added dropwise under stirring. The resulting mixture was stirred at 60 °C for 1 h and subsequently allowed to cool to room temperature. The solid formed was collected by filtration, washed thoroughly with absolute ethanol, and dried to afford a white solid (1.76 g, 9.5 mmol, 98.5% yield).

2.2 Synthesis of 2-((trifluoromethyl)sulfinyl)isoindoline-1,3-dione (2a)

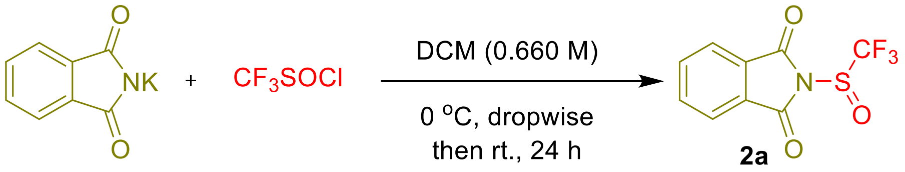

Following a modified literature procedure, [2] an oven-dried 50 mL round-bottom flask equipped with a magnetic stir bar was charged with potassium phthalimide (1.00 g, 5.37 mmol, 1.0 equiv.). The flask was evacuated and backfilled with nitrogen three times to ensure an inert atmosphere. Dry dichloromethane (11.0 mL) was then added under nitrogen, and the reaction mixture was cooled to 0 °C. A solution of trifluoromethanesulfinyl chloride (0.82 g, 5.37 mmol, 1.0 equiv.) in dry dichloromethane (5.0 mL) was added dropwise under nitrogen. The reaction mixture was gradually warmed to room temperature and stirred for 24 h. Upon completion, the mixture was filtered through a short pad of Celite and concentrated under reduced pressure. The crude product was purified by repeated recrystallization from toluene at –20 °C to afford the desired product as a white solid (0.608 g, 2.31 mmol, 43% yield).

2.3 Synthesis of *NH*-sulfoximines (1a-1j)

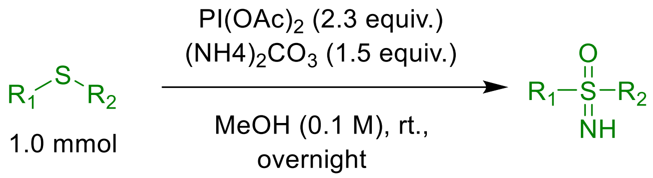

Following a modified literature procedure, [3] an oven-dried 50 mL round-bottom flask equipped with a magnetic stir bar was charged with sulfide (1.0 mmol), iodobenzene diacetate ($PhI(OAc)_2$, 2.3 mmol, 2.3 equiv.), and ammonium carbonate ($(NH_4)_2CO_3$, 1.5 mmol, 1.5 equiv.). Methanol (10.0 mL, 0.10 M) was then added, and the reaction mixture was stirred at room temperature overnight. After completion, the solvent was removed under reduced pressure, and the residue was extracted with ethyl acetate (3 × 10 mL). The combined organic layers were dried over anhydrous $MgSO_4$, filtered, and concentrated under reduced pressure. The crude product was purified by silica gel column chromatography using a mixture of hexane and ethyl acetate as the eluent to afford the desired *NH*-sulfoximine (**1a-1j**).

2.4. Synthesis of biomolecule coupled *NH*-sulfoximines (5a-5e)

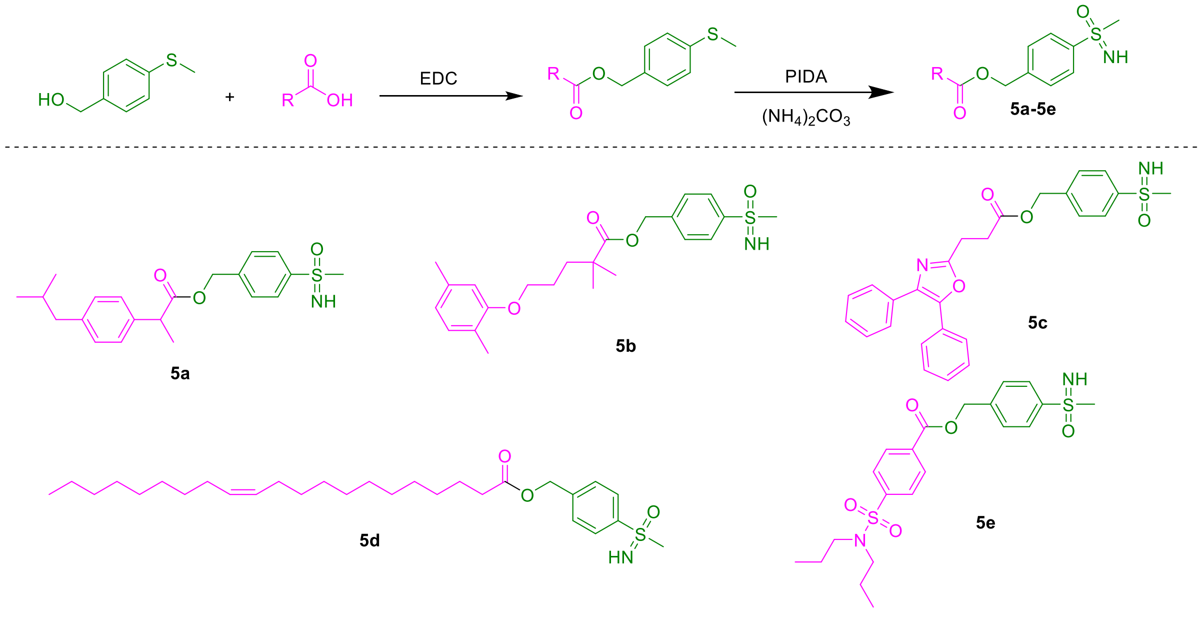

Step I: Following a modified literature procedure, [4] an oven-dried Schlenk flask equipped with a magnetic stir bar was charged with 4-(methylthio)benzyl alcohol (0.6 mmol), the corresponding carboxylic acid (1.0 equiv.), 4-dimethylaminopyridine (DMAP, 1.5 equiv.), and *N*-(3-dimethylaminopropyl)-*N′*-ethylcarbodiimide (EDC, 1.5 equiv.). Dry dichloromethane (0.2 M) was then added under an air atmosphere, and the reaction mixture was stirred at room temperature 3 h. After completion, the reaction was quenched with saturated aqueous

ammonium chloride solution and stirred for 10 minutes. The layers were separated, and the aqueous phase was extracted with dichloromethane (3 × 10 mL). The combined organic layers were washed with brine, dried over anhydrous $MgSO_4$, filtered, and concentrated under reduced pressure. The crude residue was purified by silica gel column chromatography to afford the desired ester.

Step II: Following a modified literature procedure, [3] an oven-dried 50 mL round-bottom flask equipped with a magnetic stir bar was charged with the sulfide (1.0 mmol), iodobenzene diacetate ($PhI(OAc)_2$, 2.3 mmol, 2.3 equiv.), and ammonium carbonate ($(NH_4)_2CO_3$, 1.5 mmol, 1.5 equiv.). Methanol (10.0 mL, 0.10 M) was then added, and the reaction mixture was stirred at room temperature overnight. After completion, the solvent was removed under reduced pressure, and the residue was extracted with ethyl acetate (3 × 10 mL). The combined organic layers were dried over anhydrous $MgSO_4$, filtered, and concentrated under reduced pressure. The crude product was purified by silica gel column chromatography using a mixture of hexane and ethyl acetate as the eluent to afford the desired *NH*-sulfoximine (**5a-5e**). **5a** and **5e** were matched with previous literature. [5] The rest of the new starting materials **5b-5d** were characterized and the data were presented as followed.

2.5. Characterization data of starting materials (5b-5d)

4-(*S*-methylsulfonimidoyl)benzyl-5-(2,5-dimethylphenoxy)-2,2-dimethylpentanoate (5b).

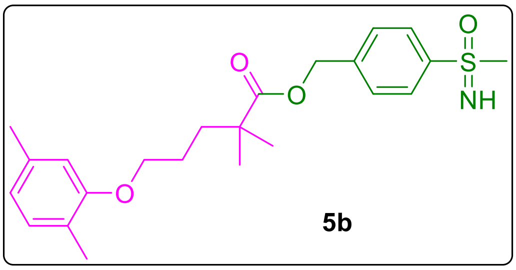

5b

The title compound was prepared according to the general experimental procedure 3.4 on a 1.0 mmol for 12 h and the product was isolated by column chromatography (Ethyl acetate/Hexane) to afford a yellow viscous gel (226 mg, 56%); ^{1}H NMR ($CDCl_3$, 400 MHz) δ 8.01 – 7.97 (m, 2H), 7.50 (d, *J* = 8.40 Hz, 2H), 6.99 (d, *J* = 7.6 Hz, 1H), 6.65 (d, *J* = 7.6 Hz, 1H), 6.59 (s, 1H), 5.17 (s, 2H), 3.90 (t, *J* = 5.8 Hz, 2H), 3.10 (s, 3H), 2.30 (s, 3H), 2.14 (s, 3H), 1.77 – 1.72 (m, 4H), 1.26 (s, 6H). $^{13}C\{^1H\}$ NMR ($CDCl_3$, 101 MHz) δ 177.3, 156.9, 143.0, 142.0, 136.5, 130.3, 128.2, 128.0, 123.5, 120.8, 112.0, 67.8, 65.0, 46.1, 42.2, 37.0, 25.1, 21.4, 15.7; HRMS (ESI) calculated for $C_{23}H_{31}NO_4NaS$ [M+Na]; 440.1866 found 440.1859.

4-(*S*-methylsulfonimidoyl)benzyl-3-(4,5-diphenyloxazol-2-yl)propanoate (5c). The title compound was prepared according to the general experimental procedure 3.4 on a 1.0 mmol for 12 h and the product was isolated by column chromatography (Ethyl acetate/Hexane) to afford a colourless viscous gel (360 mg, 78%); ^{1}H NMR ($CDCl_3$, 400 MHz) δ 7.91 – 7.87 (m, 2H), 7.62 – 7.58 (m, 2H), 7.55 – 7.52 (m, 2H), 7.48 (d, J = 8.0 Hz, 2H), 7.39 – 7.30 (m, 6H), 5.24 (s, 2H), 3.21 (t, J = 7.2 Hz, 2H), 3.04 (s, 3H), 3.01 (t, J = 7.2 Hz, 2H). ^{13}C{^{1}H} NMR ($CDCl_3$, 101 MHz) δ 171.6, 161.4, 145.5, 143.1, 141.3, 135.1, 132.3, 128.8, 128.6, 128.5, 128.3, 128.1, 127.9, 127.8, 126.4, 65.3, 46.0, 30.9, 23.4; HRMS (ESI) calculated for $C_{26}H_{24}N_2O_4NaS$ [M+Na]; 483.1349 found 483.1344.

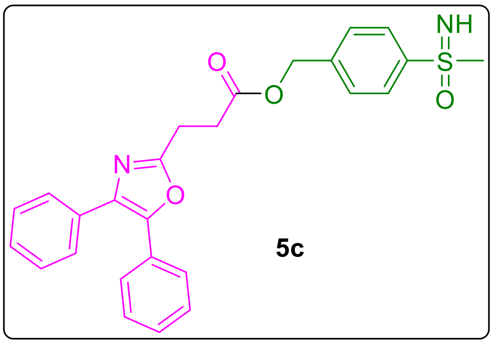

4-(*S*-methylsulfonimidoyl)benzyl-(*Z*)-henicos-12-enoate (5d). The title compound was prepared according to the general experimental procedure 3.4 on a 1.0 mmol for 12 h and the product was isolated by column chromatography (Ethyl acetate/Hexane) to afford a colourless viscous gel (350 mg, 69%); ^{1}H NMR ($CDCl_3$, 400 MHz) δ 8.06 – 7.96 (m, 2H), 7.54 – 7.49 (m, 2H), 5.34 (t, J = 5.4 Hz, 2H), 5.18 (s, 2H), 3.11 (s, 3H), 2.38 (t, J = 7.6 Hz, 2H), 2.00 (dd, J = 12.2, 6.6 Hz, 4H), 1.72 – 1.58 (m, 2H), 1.32 – 1.23 (m, 28H), 0.87 (t, J = 6.8 Hz, 3H). ^{13}C{^{1}H} NMR ($CDCl_3$, 101 MHz) δ 173.34, 143.0, 141.8, 129.9, 129.8, 128.4, 128.0, 64.8, 46.1, 34.2, 31.9, 29.7, 29.6, 29.5, 29.4, 29.3, 29.2, 29.1, 27.2, 24.9, 22.6, 14.1; HRMS (ESI) calculated for $C_{30}H_{51}NO_3NaS$ [M+Na]; 528.3482 found 528.3476.

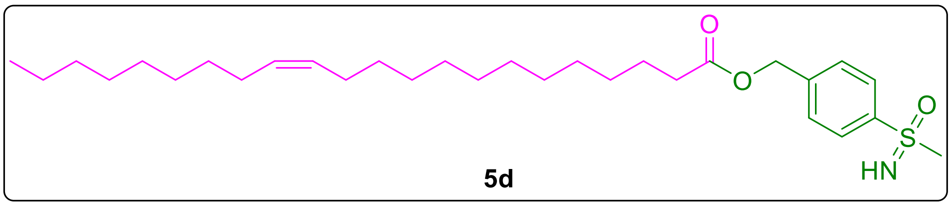

3. Experimental procedure for *N*-trifluoromethylsulfonyl sulfoximines (3a-3j and 6a-6e)

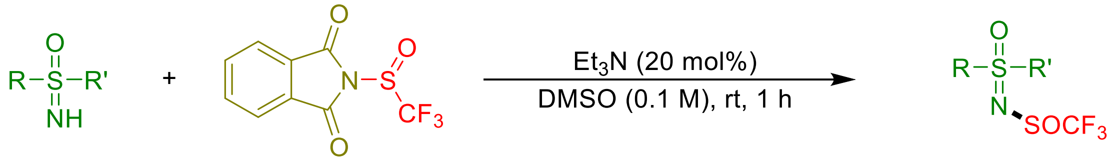

1a-1j, **5a-5e** **2a** **3a-3j & 6a-6e**

An oven dried 15 mL reaction tube was charged with (**1a-1j** and **5a-5e**) (0.2 mmol, 1.0 equiv.), **2a** (0.3 mmol, 1.5 equiv.), Et_3N (20 mol%), DMSO (0.1 M, 2.0 mL). The resulting mixture was stirred at room temperature for about 1 h. After the completion of the reaction, reaction mixture was diluted with 10 mL of water. The aqueous layer was extracted with Ethyl acetate (3 × 10 mL), and the combined organic layer was washed with brine solution (1 × 5 mL). The final organic layer was then dried over $MgSO_4$ and concentrated under reduced pressure to get the crude product. The obtained crude product was purified using column chromatography by eluting with ethyl acetate/hexane to afford desired product (**3a-3j** & **6a-6e**) up to 57-81% yields.

4. General procedure for the integrated continuous flow approach

4.1 Table S5 Optimization conditions of continuous flow method [a,b]

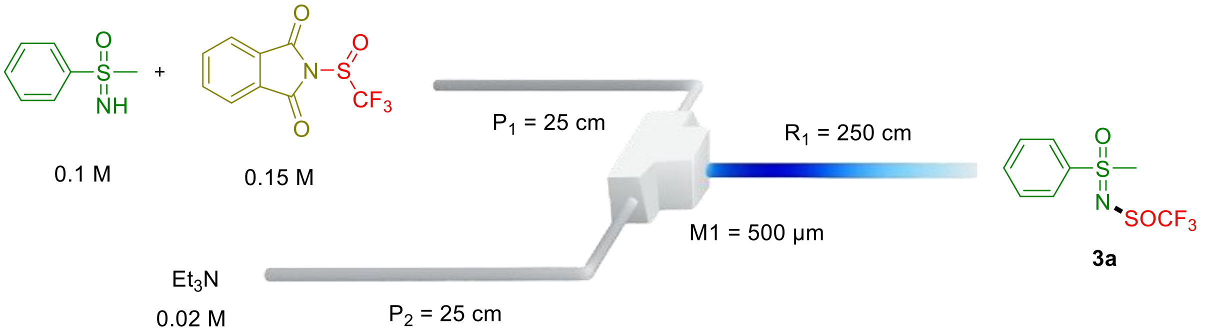

S. No.	Flow rate (P_1:P_2)	Reaction time (s)	Yield (%) [b]
1.	1000:1000 µL min^{-1}	45	87
2.	2000:2000 µL min^{-1}	23	86
3.	4000:4000 µL min^{-1}	12	86
**4.**	**8000:8000 µL min^{-1}**	**6**	**86**
5.	16000:16000 µL min^{-1}	3	82

Reaction condition: [a] A continuous flow system consists of two reagents delivering units (P_1, and P_2, Φ= 800 µm, length L= 25 cm) T-shaped micromixer (M1, Φ = 500 µm), one microtube reactor (R_1= 250 cm, Φ = 800 µm) a Reaction was performed under standard optimized conditions (F_1, F_2 = x µL/min) and the product was collected for y seconds. [b] isolated yield.

4.2 Experimental procedure for *N*-trifluoromethylsulfonyl sulfoximines in continuous flow method (3a, 3b, 3e, 3g & 3j)

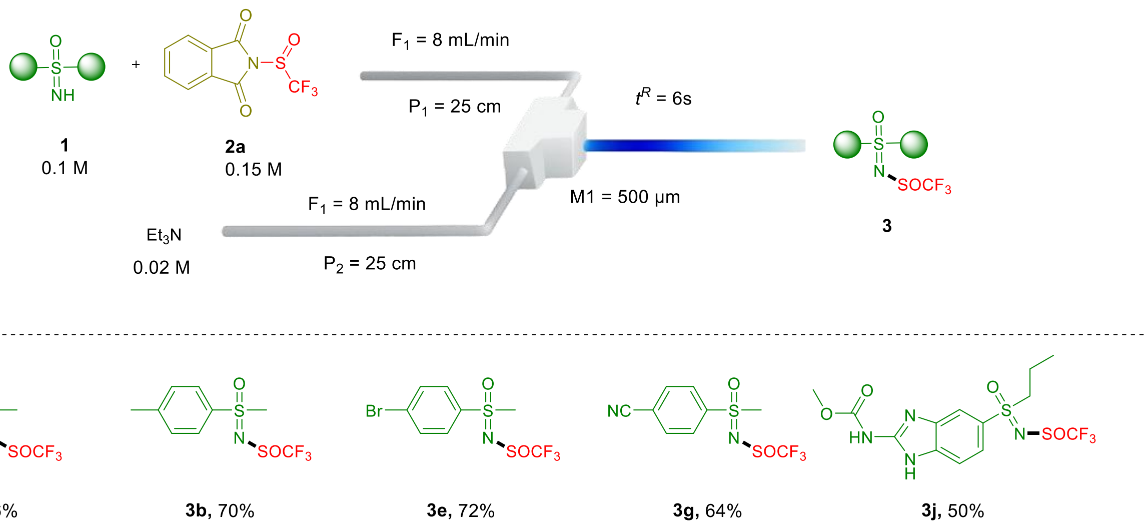

A microreactor system consists of one T-shaped micromixer (M1), one microtube reaction (R_1), two precooling units P_1 (inner diameter Φ = 800 µm, length L = 25 cm), P_2 (Φ = 800 µm, length L = 25 cm). A solution containing **1 and 2a** (0.1 M & 0.15 M in DMSO) (flow rate: F_1 = 8 mL/min) and a solution of triethylamine (0.02 M in DMSO) (flow rate: F_2 = 8 mL/min) were introduced into M1 (Φ = 500 µm) by syringe pumps. The resulting solution was passed through microtubing (R_1= 250 cm, Φ = 800 µm) with room temperature. After a steady state was reached, the solution was collected for 6 seconds in a vial. The resulting solution was extracted with ethyl acetate (10 mL x 3), and the organic layer was combined and washed with brine solution (5 mL). The organic layer was dried over anhydrous $MgSO_4$, filtered, and concentrated under a vacuum. The crude product was purified by column chromatography (EtOAc/Hexane) and obtained **3a, 3b, 3e, 3g** and **3j** in 50-86% yields.

5. Experimental procedure for gram scale synthesis

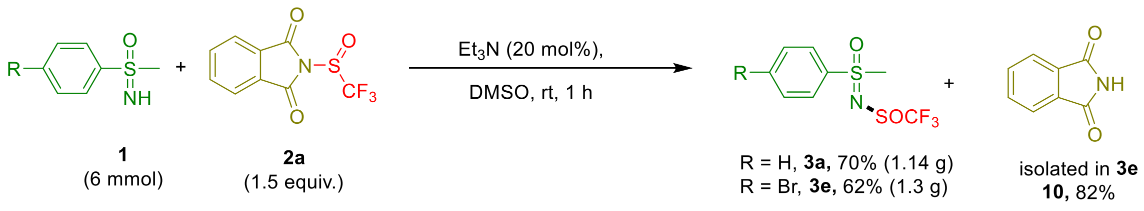

An oven-dried 250 mL round-bottom flask equipped with a magnetic stir bar was charged with **1** (6.0 mmol, 1.0 equiv.), **2a** (9.0 mmol, 1.5 equiv.), Et_3N (20 mol%), and DMSO (0.1 M, 60

mL). The resulting mixture was stirred at room temperature for approximately 1 h. After completion of the reaction, the reaction mixture was diluted with water (100 mL). The aqueous layer was extracted with ethyl acetate (3 × 30 mL), and the combined organic layers were washed with brine (15 mL). The organic layer was dried over $MgSO_4$ and concentrated under reduced pressure to afford the crude product. Diethyl ether was then added to the crude mixture, and the resulting white solid **10** was collected by filtration (1086 mg, 7.38 mmol, 82% yield). The filtrate was concentrated under reduced pressure, and the residue was purified by flash silica gel chromatography using ethyl acetate/hexane as the eluent to afford the desired products **3a** (1.14 g, 4.20 mmol, 70% yield) and **3e** (1.30 g, 3.72 mmol, 62% yield), respectively.

6. Control studies

6.1 Experimental procedure for radical trapping experiments

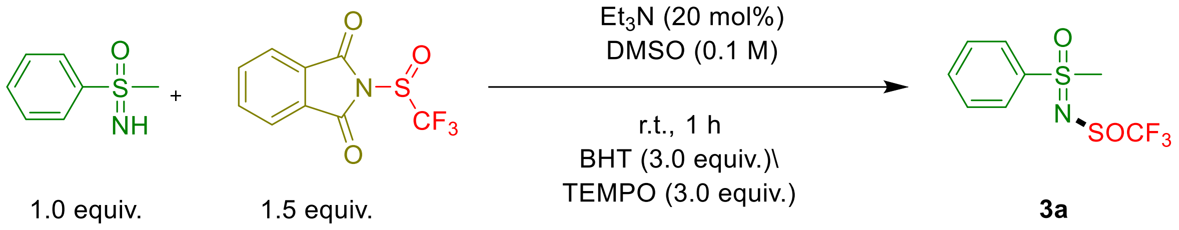

An oven dried 15 mL reaction tube was charged with (**1a**) (0.2 mmol, 1.0 equiv.), **2a** (0.3 mmol, 1.5 equiv.), Et_3N (20 mol%), BHT/TEMPO (3.0 equiv.) and DMSO (0.1 M, 2.0 mL). The resulting mixture was stirred at room temperature for about 1 h. After the completion of the reaction, reaction mixture was diluted with 10 mL of water. The aqueous layer was extracted with Ethyl acetate (3 × 10 mL), and the combined organic layer was washed with brine solution (1 × 5 mL). The final organic layer was then dried over $MgSO_4$ and concentrated under reduced pressure to get the crude product. The obtained crude product was purified using column chromatography by eluting with ethyl acetate/hexane to afford desired product (**3a**) in 76 & 79% yields respectively.

6.2 Experimental procedure for study of triethylamine role in the reaction

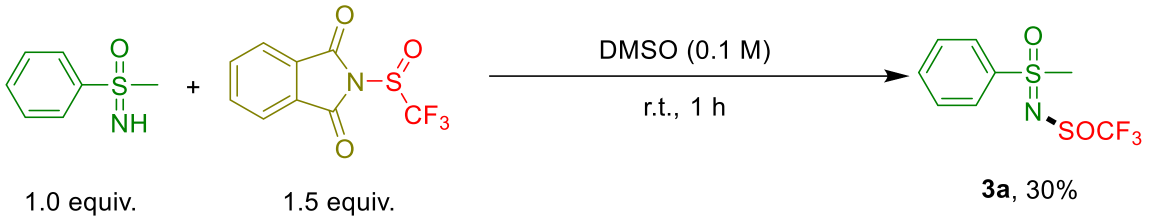

An oven dried 15 mL reaction tube was charged with (**1a**) (0.2 mmol, 1.0 equiv.), **2a** (0.3 mmol, 1.5 equiv.), and DMSO (0.1 M, 2.0 mL). The resulting mixture was stirred at room temperature for about 1 h. After the completion of the reaction, reaction mixture was diluted with 10 mL of water. The aqueous layer was extracted with Ethyl acetate (3 × 10 mL), and the combined organic layer was washed with brine solution (1 × 5 mL). The final organic layer was then dried over $MgSO_4$ and concentrated under reduced pressure to get the crude product. The obtained crude product was purified using column chromatography by eluting with ethyl acetate/hexane to afford desired product (**3a**) in 30% yields.

6.3 Result and discussion of control studies

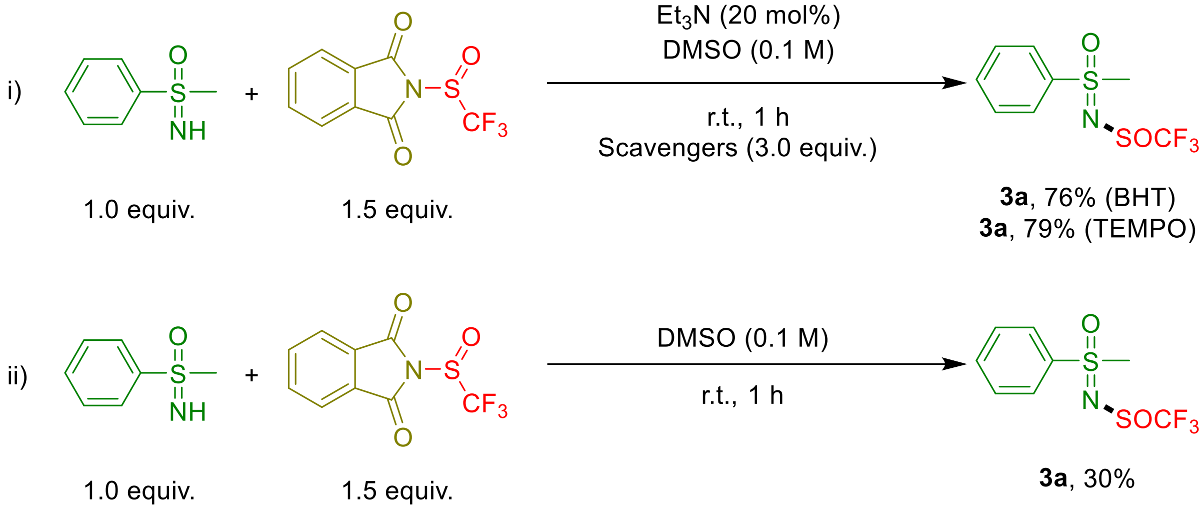

Scheme S1. Control studies

Mechanistic investigations were carried out to gain further insight into the reaction pathway. Initially, the radical trap-ping experiments were carried out to assess whether the reaction proceeds via a radical mechanism. The reaction of 1a with 2a was carried out in the presence of BHT or TEMPO, which are well known as radical scavengers. In both cases, the reaction proceeded smoothly, suggesting that a radical pathway is unlikely (Scheme S1 (i)). Next, the role of Et_3N was examined by conducting the reaction in the absence of base. Under these

conditions, the yield de-creased significantly to 30% (3a), indicating that sulfoximine 1a itself can act as a nucleophile to promote the reaction. However, due to its relatively weak nucleophilicity, the reaction proceeds inefficiently, leading to a lower yield (Scheme S1 (ii)).

7. Mechanistic studies

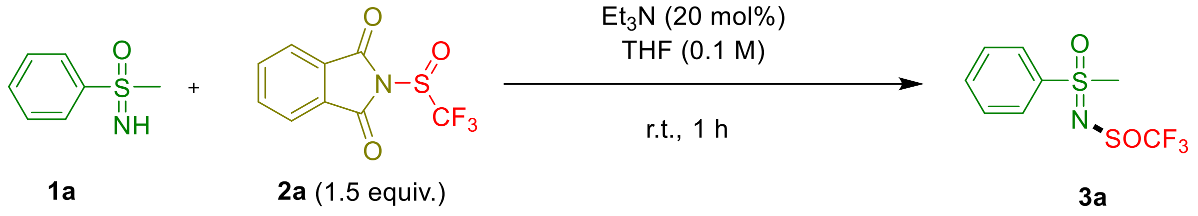

An oven dried 15 mL reaction tube was charged with (**1a**) (0.1 mmol, 1.0 equiv.), **2a** (0.15 mmol, 1.5 equiv.), Et_3N (20 mol%), THF (0.1 M, 1.0 mL). The resulting mixture was stirred at room temperature for about 1 h, the crude mixture was subjected to ^{19}F NMR spectroscopy in the presence of $PhCF_3$ (14.6 mg, 0.100 mmol).

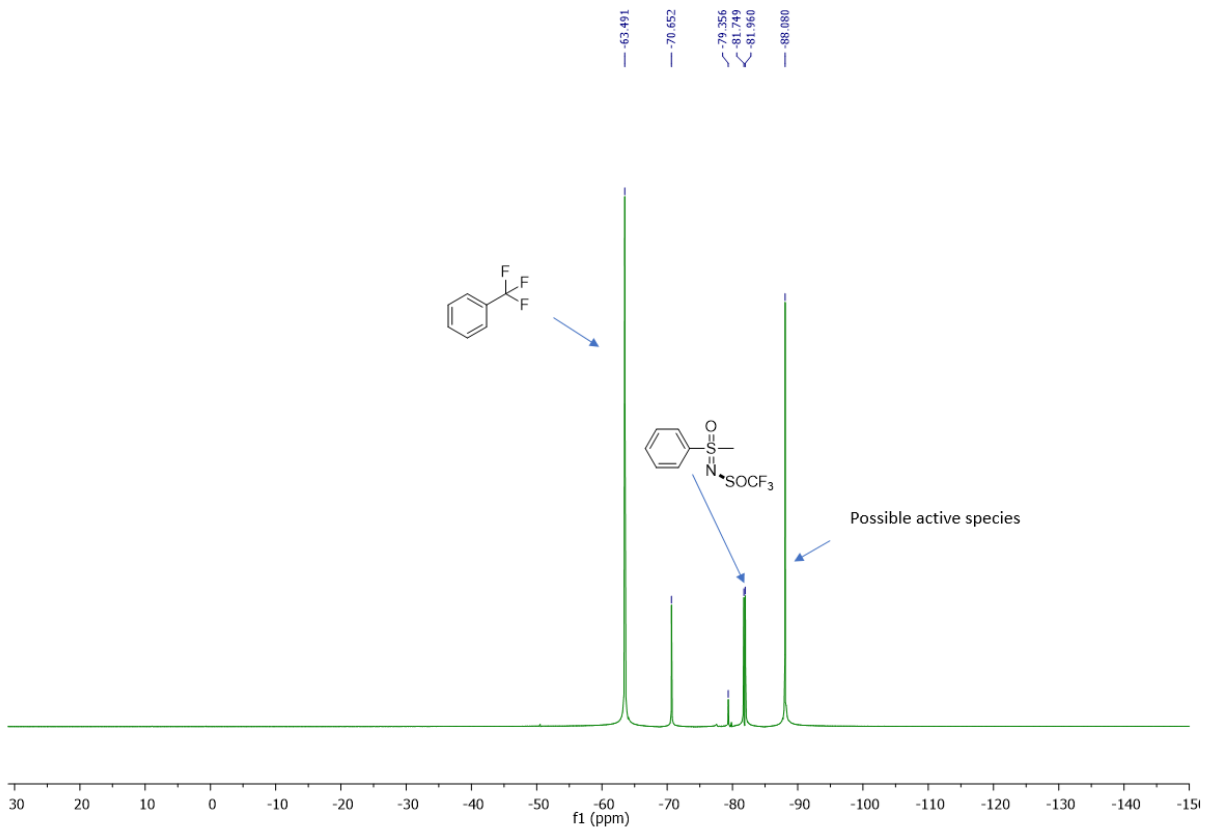

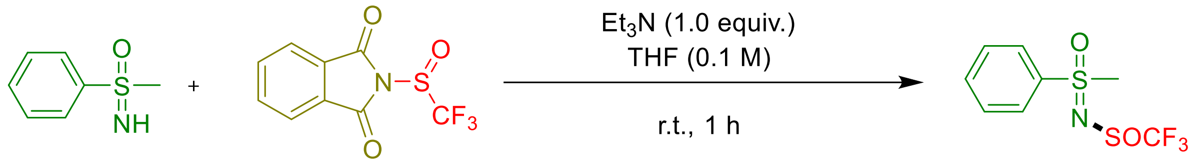

An oven dried 15 mL reaction tube was charged with (**1a**) (0.1 mmol, 1.0 equiv.), **2a** (0.1 mmol, 1.5 equiv.), Et_3N (1.0 equiv.), THF (0.1 M, 1.0 mL). The resulting mixture was stirred at room temperature for about 1 h, the crude mixture was subjected to ^{19}F NMR spectroscopy in the presence of $PhCF_3$ (14.6 mg, 0.100 mmol).

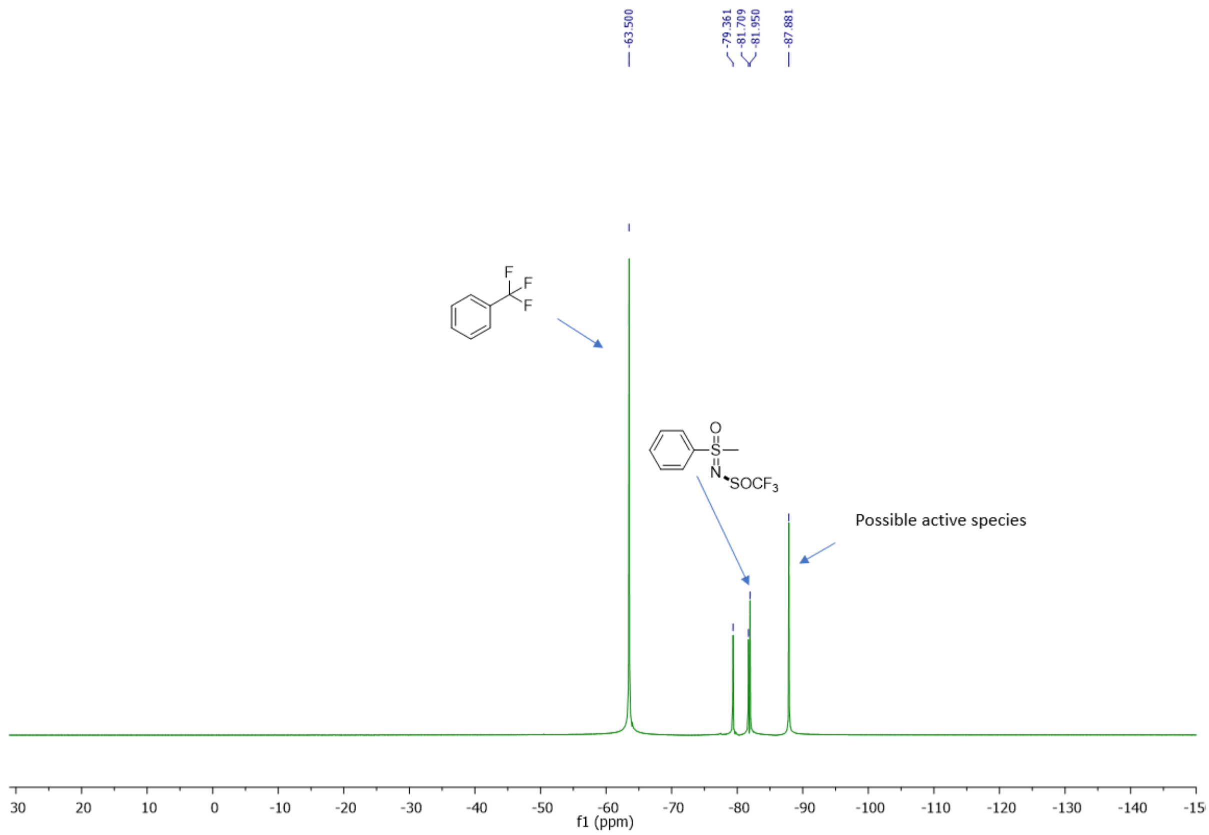

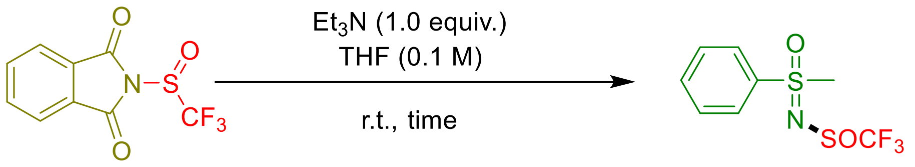

An oven dried 15 mL reaction tube was charged with **2a** (0.1 mmol, 1.0 equiv.), Et_3N (0.1 mmol, 1.0 equiv.), THF (0.1 M, 1.0 mL). The resulting mixture was stirred at room temperature

for t h, the crude mixture was subjected to ^{19}F NMR spectroscopy in the presence of $PhCF_3$ (14.6 mg, 0.1 mmol).

1. t = 0 min

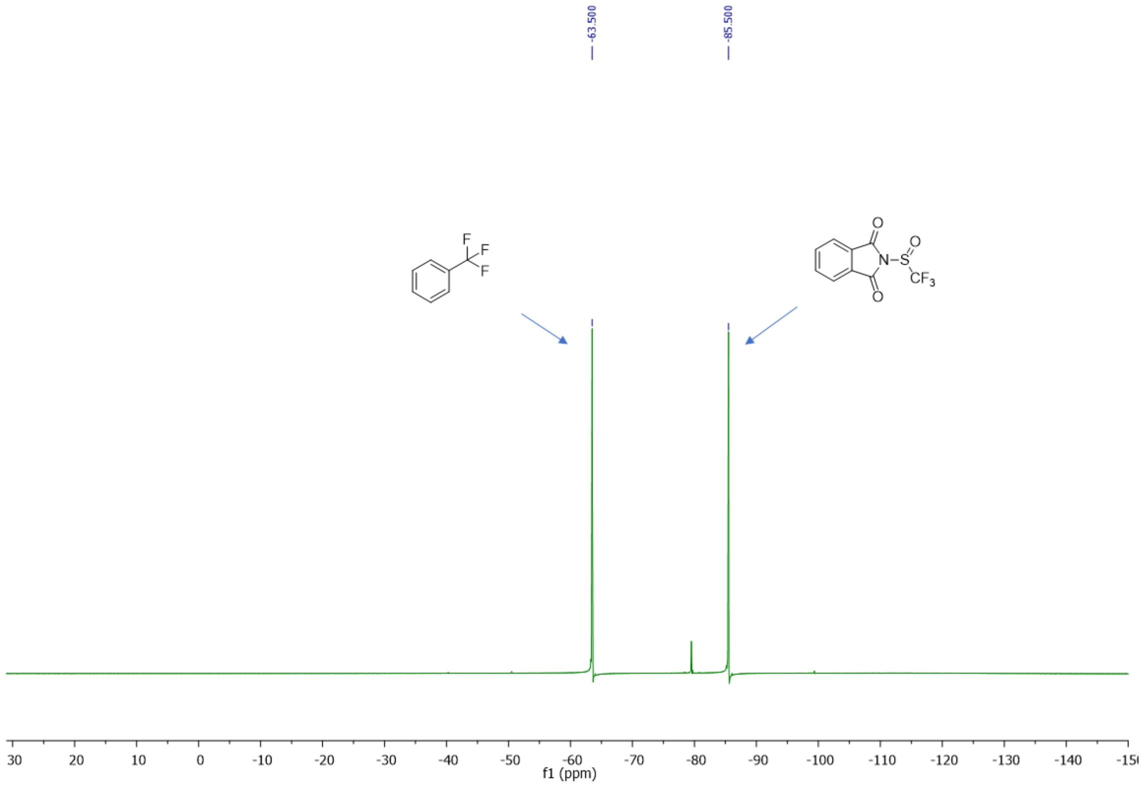

2. t = 30 min

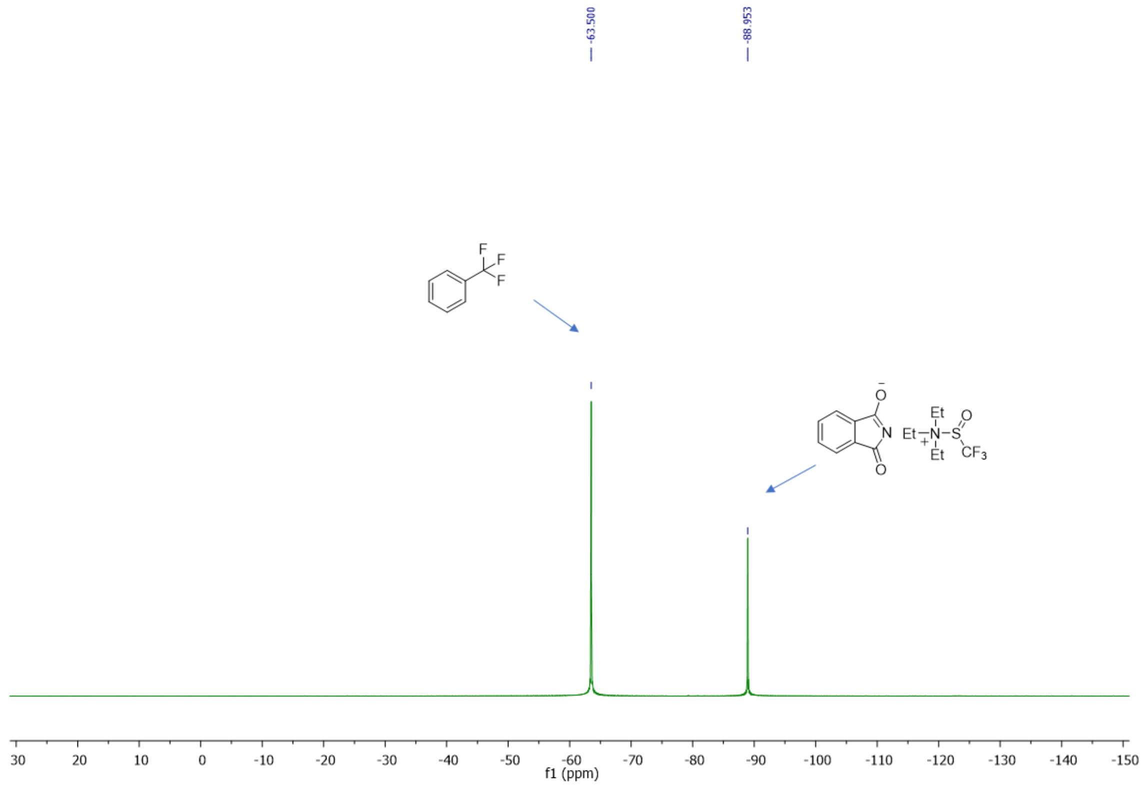

3. t = 1 h

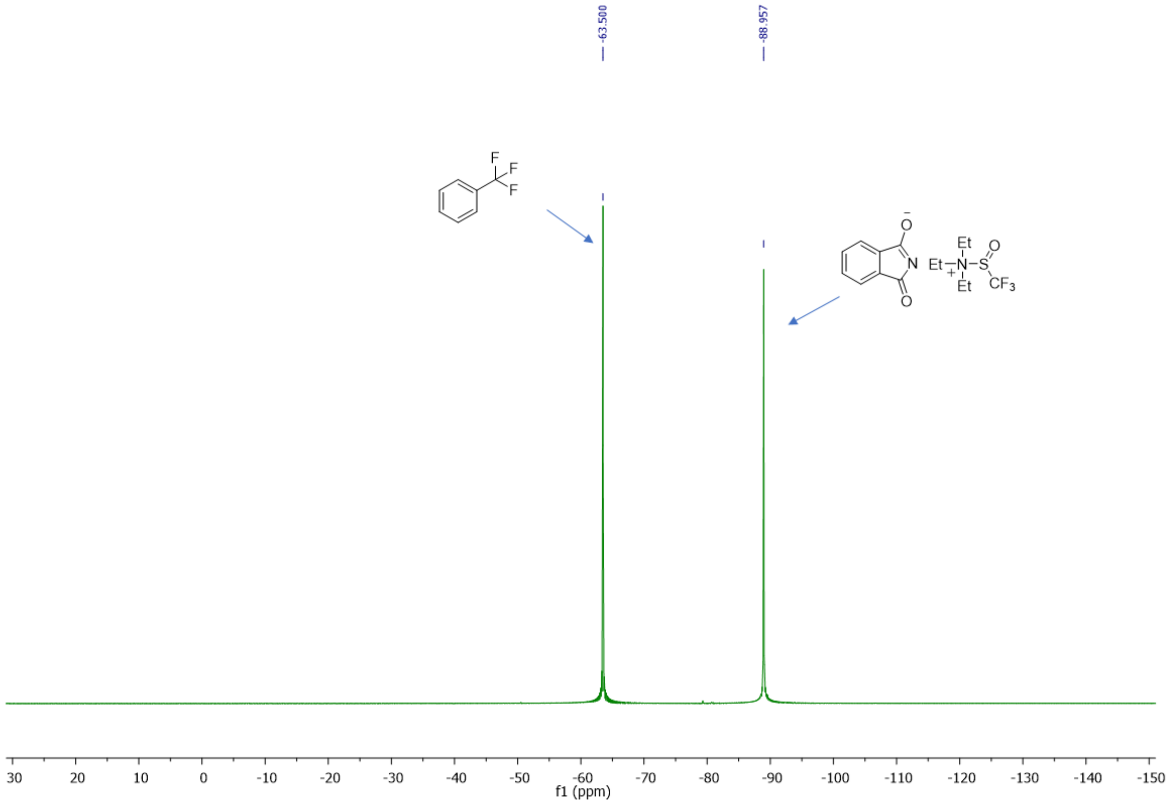

4. t = 12 h

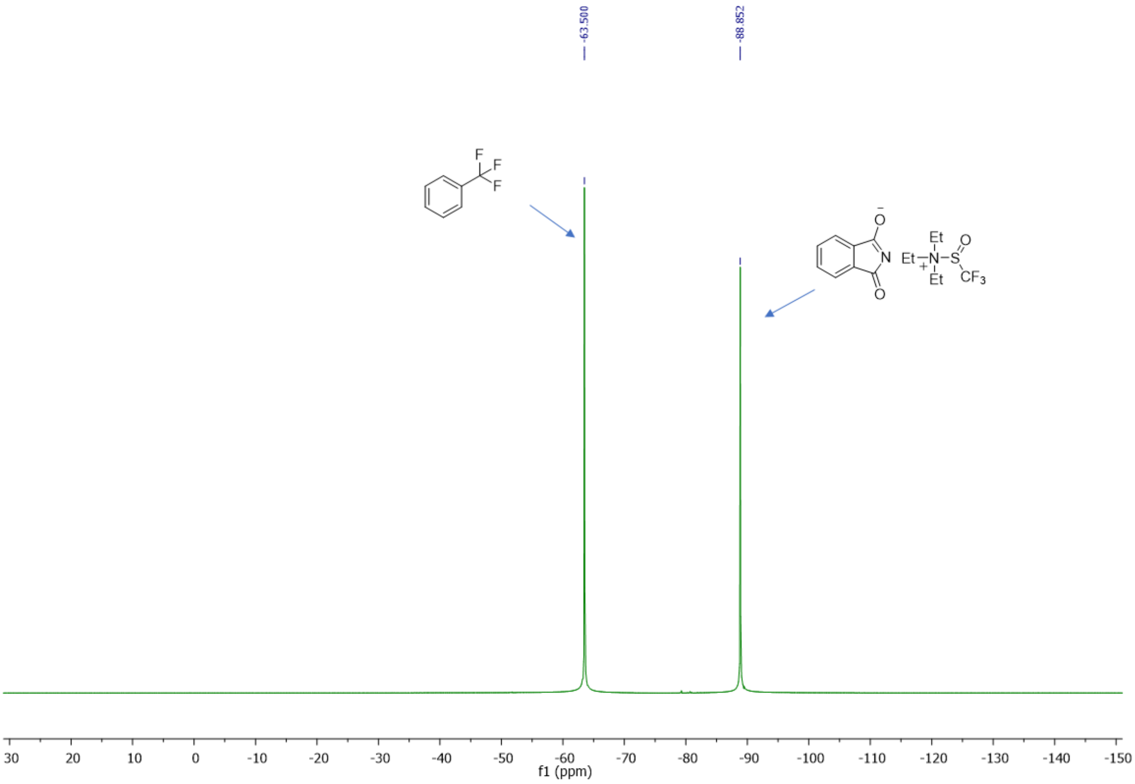

8. References

(1) Bertrand, G. R., V. D.; Raynier, B.; Derrieu, G. Environment friendly reagents and process for halogenoalkylsulfinylation of organic compounds. EP1331222A1, 2003.

(2) Kim, B.; Park, J.; Cho, C.-W. Synthesis of N-trifluoromethanesulfinyl ketimines by cascade trifluoromethylthiolation/rearrangement of ketoximes. *Org. Lett.* **2021**, *23* (12), 4603-4607.

(3) Smith, B. I.; Knight, N. M.; Knox, G. J.; Lindsay, D. M.; Paterson, L. C.; Bergare, J.; Elmore, C. S.; Bragg, R. A.; Kerr, W. J. Selective deuteration and tritiation of pharmaceutically relevant sulfoximines. *Angew. Chem. Int. Ed.* **2025**, *64* (5), e202417179.

(4) Mondal, S.; Debnath, S.; Lo, R.; Maity, S. Photoredox Activation of Donor-Acceptor Cyclopropanes: Distonic Radical Cation Reactivity in [3+ 2] Cycloaddition Reactions. *Angew. Chem. Int. Ed.* **2025**, *64* (7), e202419426.

(5) Alam, T.; Patel, B. K. Electrochemical N-Aroylation of Sulfoximines by Using Benzoyl Hydrazines with H2 Generation. *Chem. Eur. J.* **2024**, *30* (9), e202303444.

(6) Yang, D.; Xiao, Q.; Zhou, Y.; Zhang, H.; Chen, T.; Shi, M. Decarboxylative sulfinamidation of N-sulfinylamines with carboxylic acids via a photochemical iron-mediated ligand-to-metal charge transfer process. *Chem. Commun.* **2025**.

(7) Karpov, A. S.; Müller, T. J. Straightforward novel one-pot enaminone and pyrimidine syntheses by coupling-addition-cyclocondensation sequences. *Synthesis* **2003**, *2003* (18), 2815-2826.

9. Copies of ^{1}H, ^{13}C and ^{19}F Data

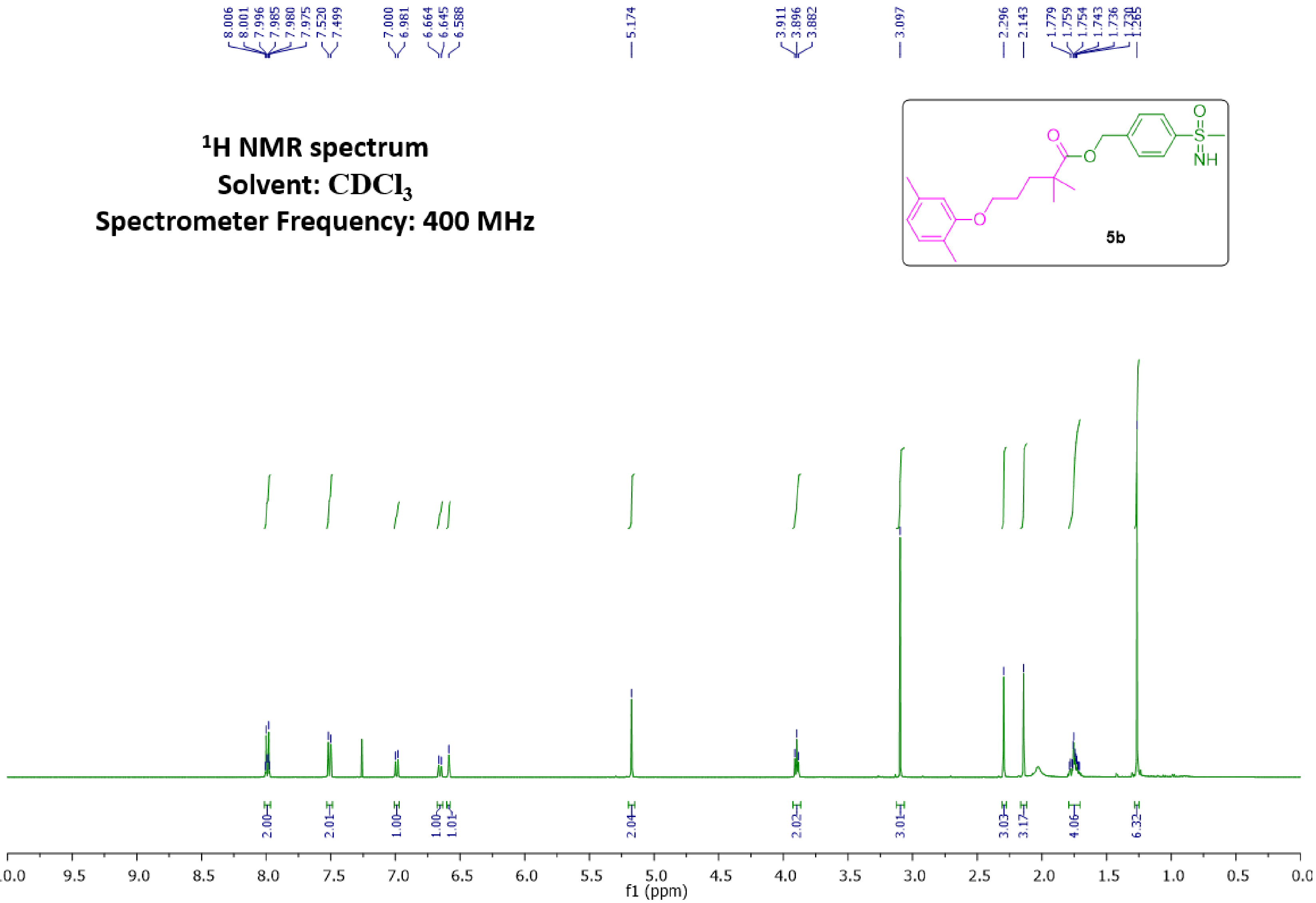

^{13}C {^{1}H} NMR spectrum
Solvent: $CDCl_3$
Spectrometer Frequency: 100 MHz

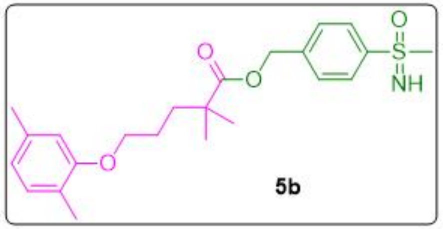

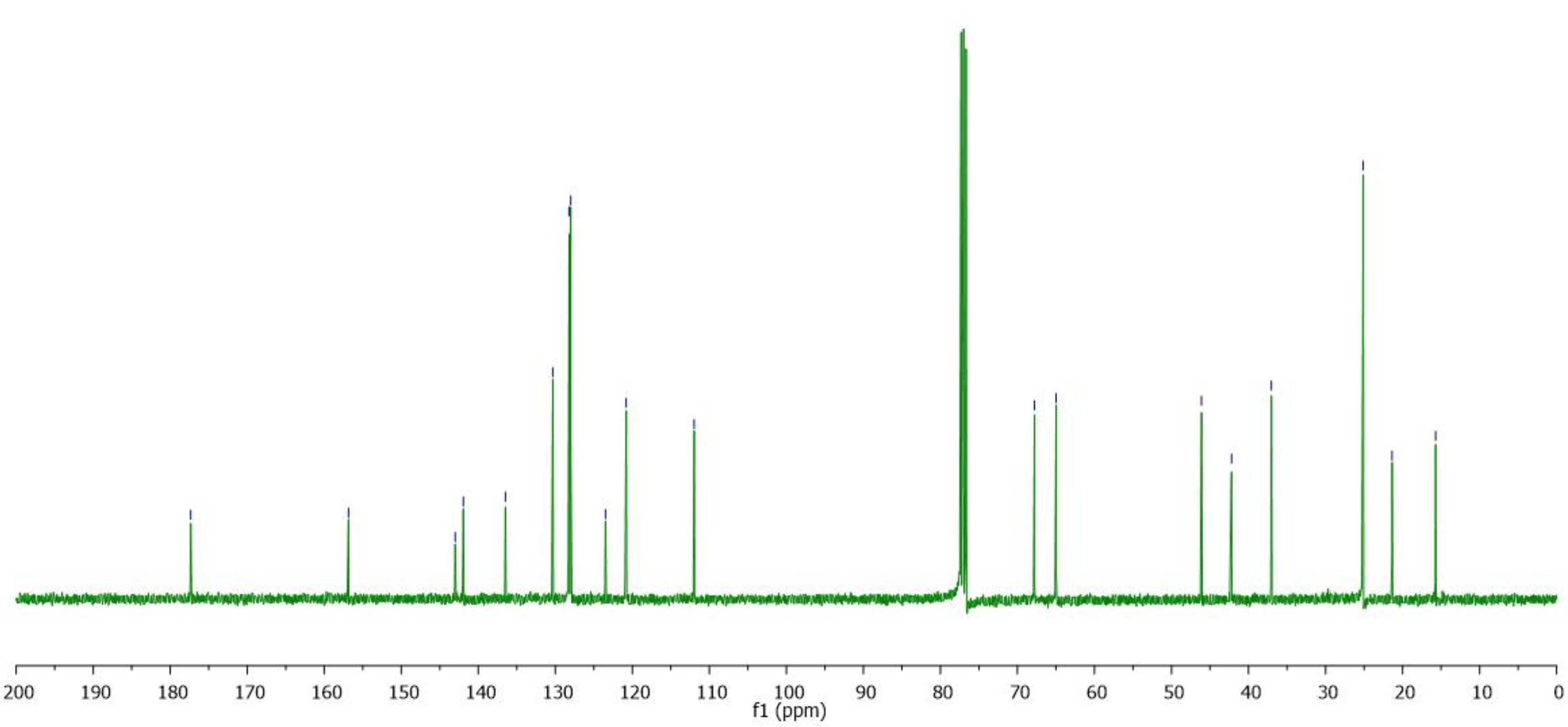

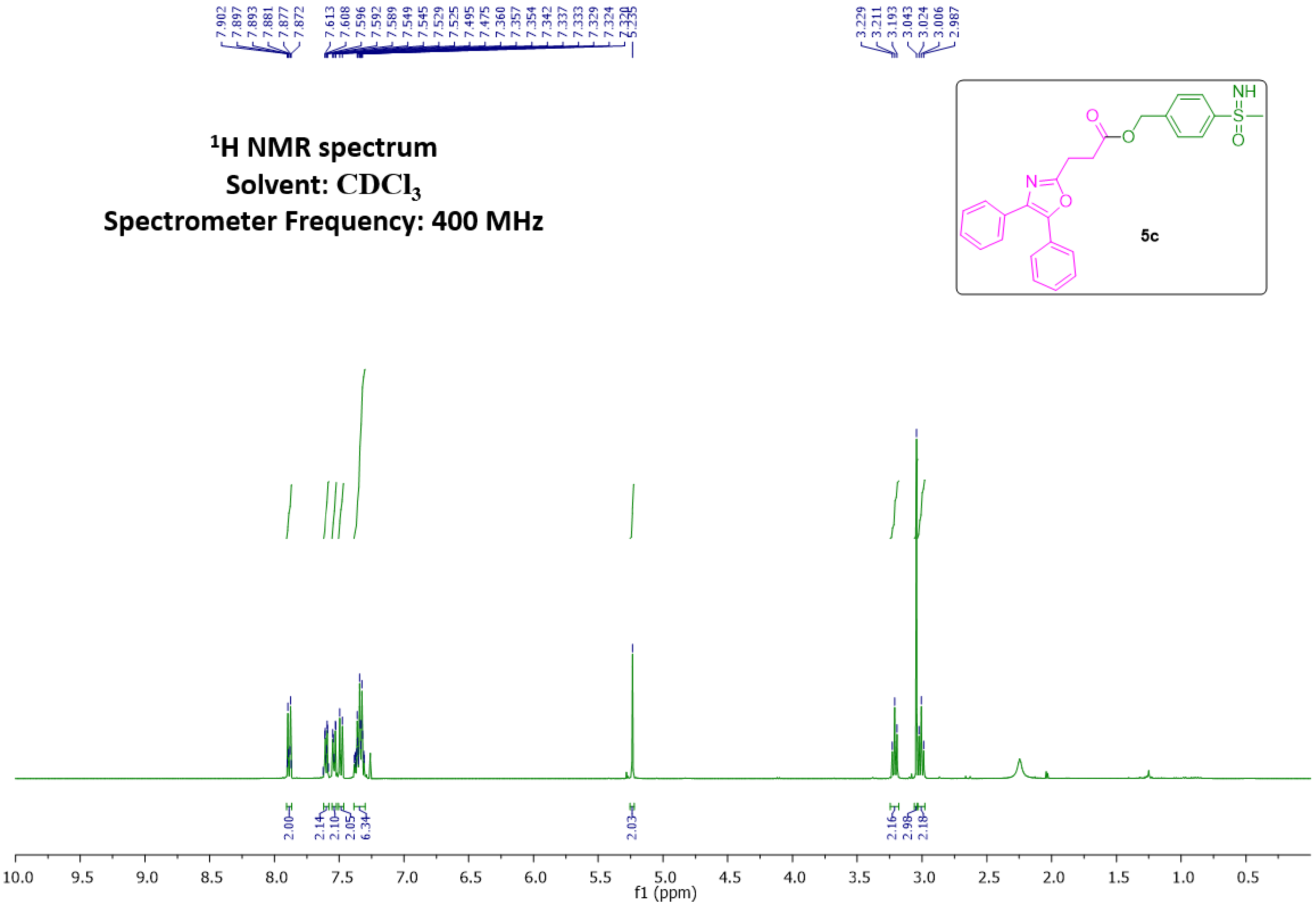

7.902
7.897
7.893
7.881
7.877
7.872
7.613
7.608
7.596
7.592
7.589
7.549
7.545
7.529
7.525
7.495
7.475
7.360
7.357
7.354
7.342
7.337
7.333
7.329
7.324
7.320
5.235
3.229
3.211
3.193
3.043
3.024
3.006
2.987
¹H NMR spectrum
Solvent: CDCl3
Spectrometer Frequency: 400 MHz
NH
S
O
O
O
N
O
5c
2.00
2.14
2.10
2.05
6.34
2.03
2.16
2.98
2.18
10.0 9.5 9.0 8.5 8.0 7.5 7.0 6.5 6.0 5.5 5.0 4.5 4.0 3.5 3.0 2.5 2.0 1.5 1.0 0.5
f1 (ppm)

^{13}C {^{1}H} NMR spectrum
Solvent: $CDCl_3$
Spectrometer Frequency: 100 MHz

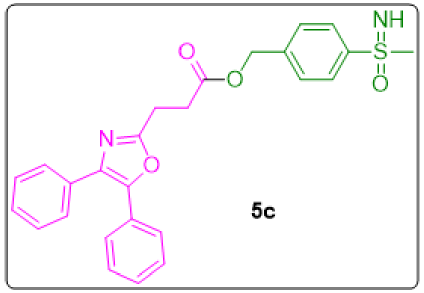

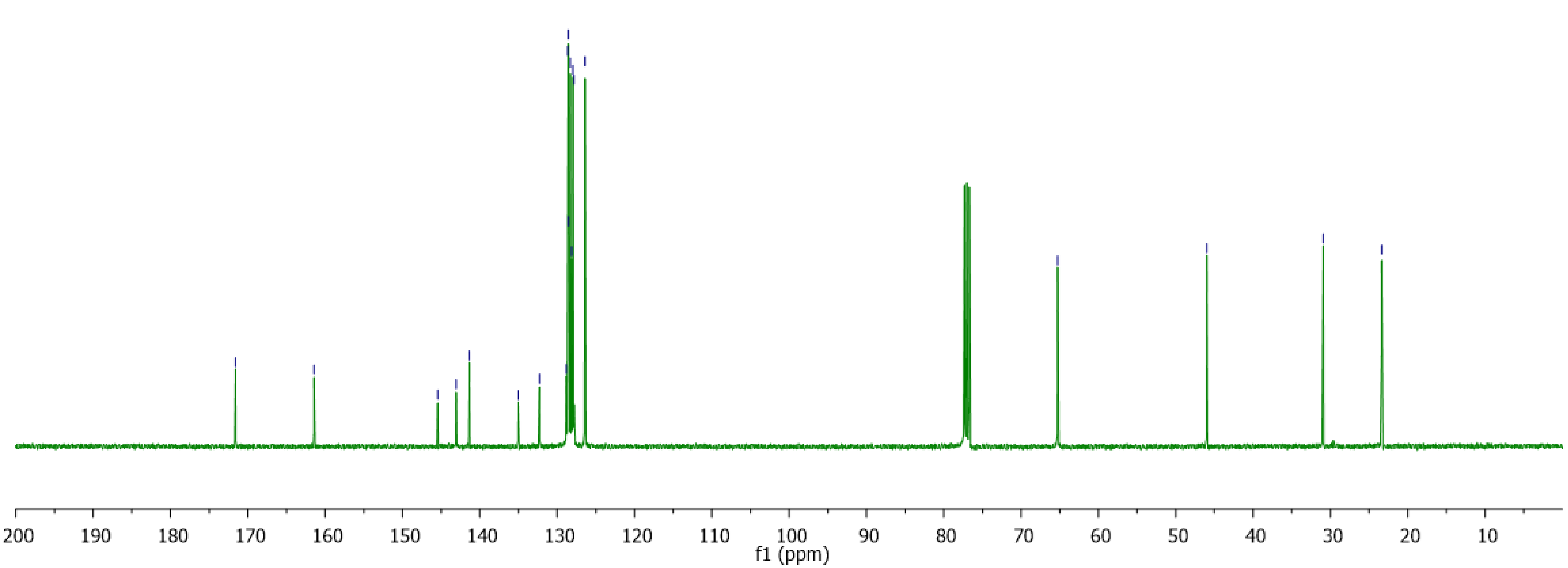

^{1}H NMR spectrum
Solvent: $CDCl_3$
Spectrometer Frequency: 400 MHz

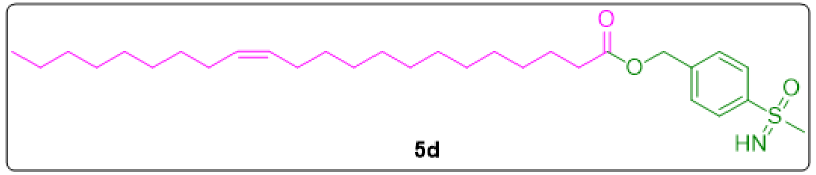

5d

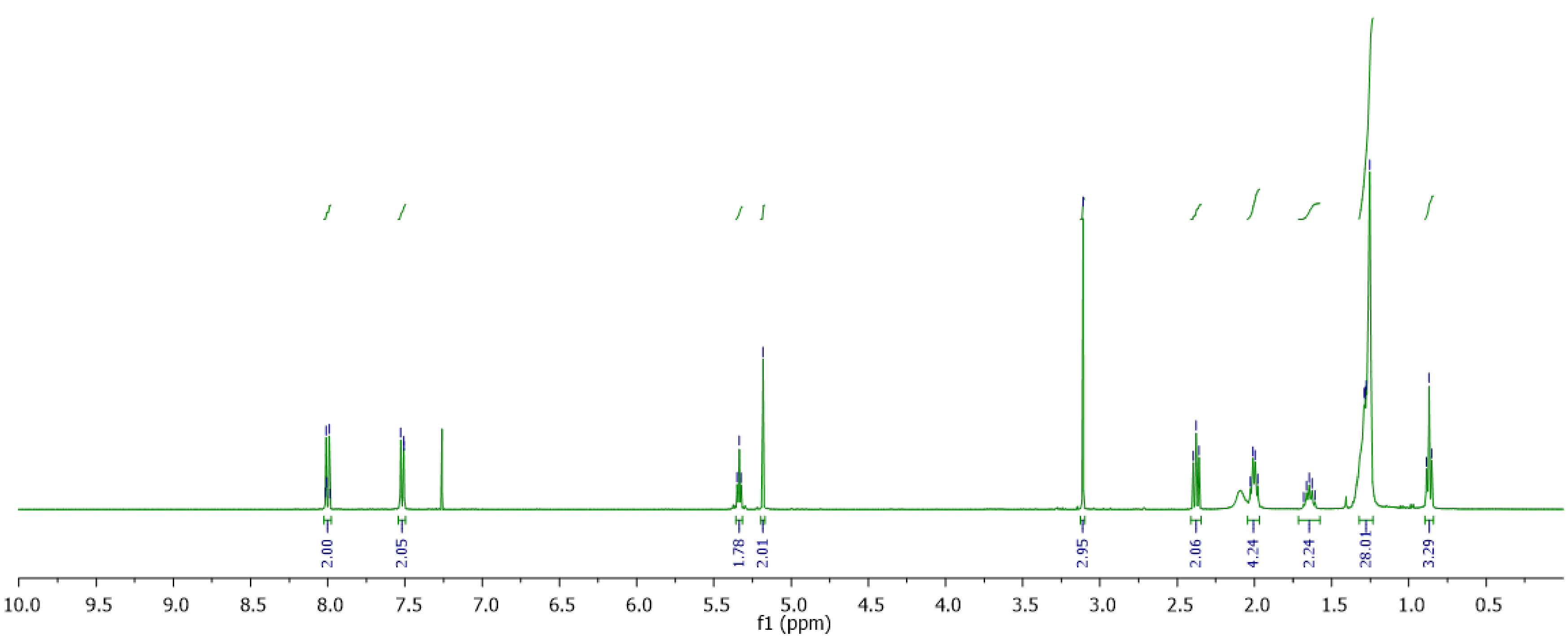

$^{13}C\ \{^{1}H\}$ NMR spectrum
Solvent: $CDCl_3$
Spectrometer Frequency: 100 MHz

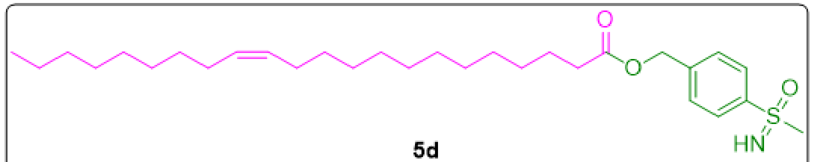
5d

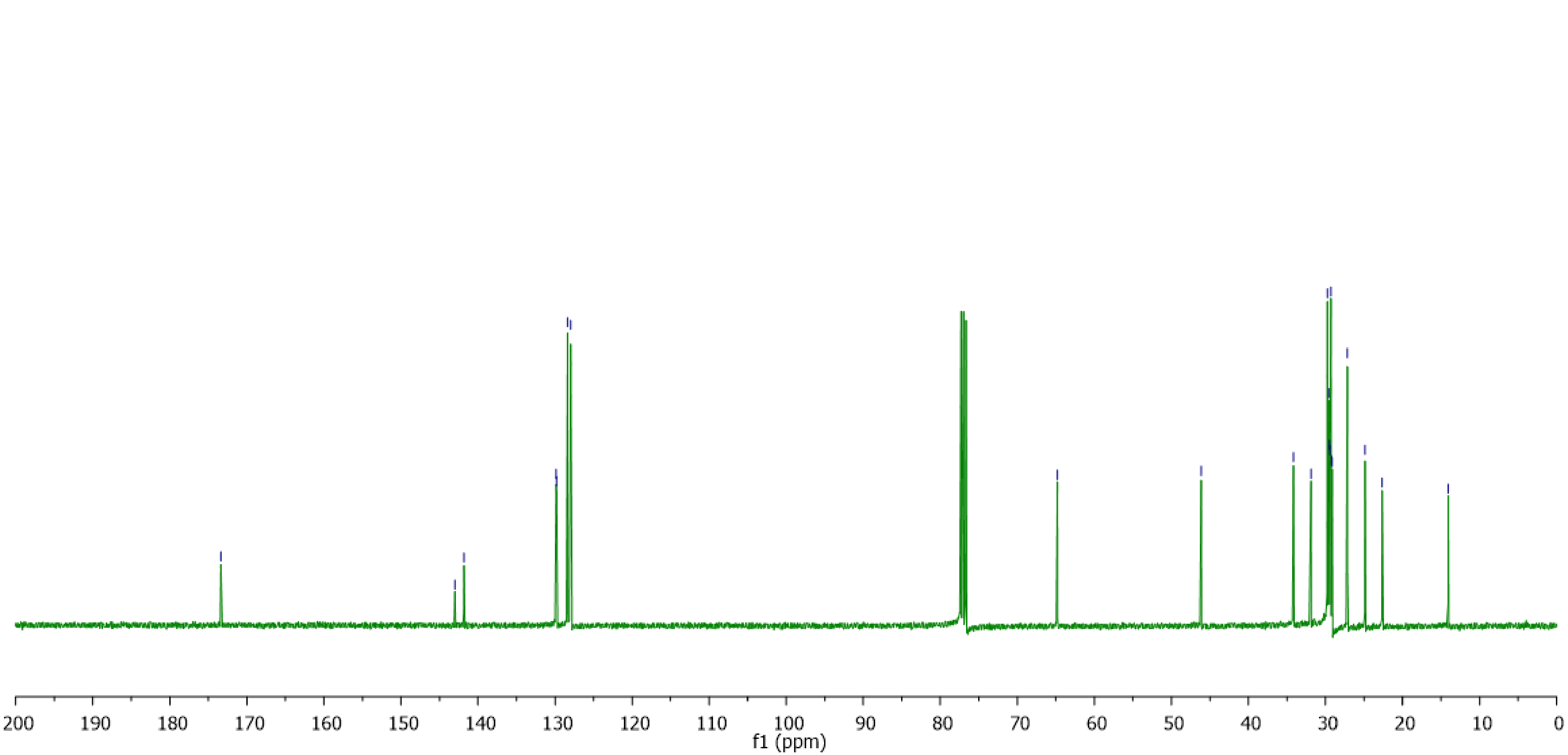

^{1}H NMR spectrum
Solvent: $CDCl_3$
Spectrometer Frequency: 400 MHz

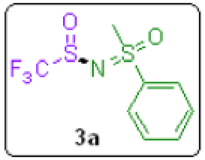

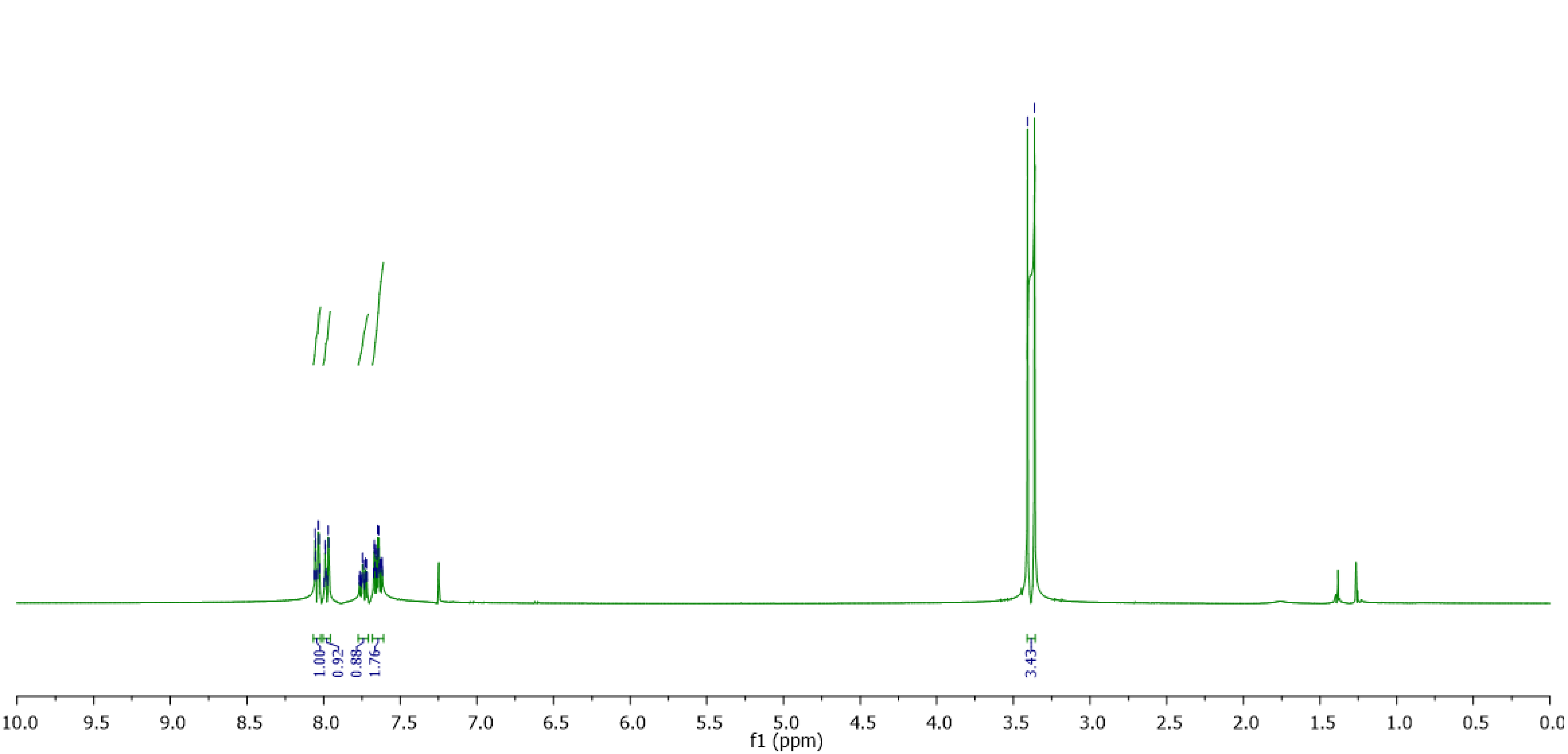

$^{13}C\ \{^{1}H\}$ NMR spectrum
Solvent: $CDCl_3$
Spectrometer Frequency: 100 MHz

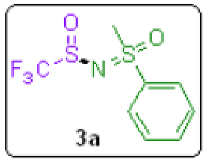

3a

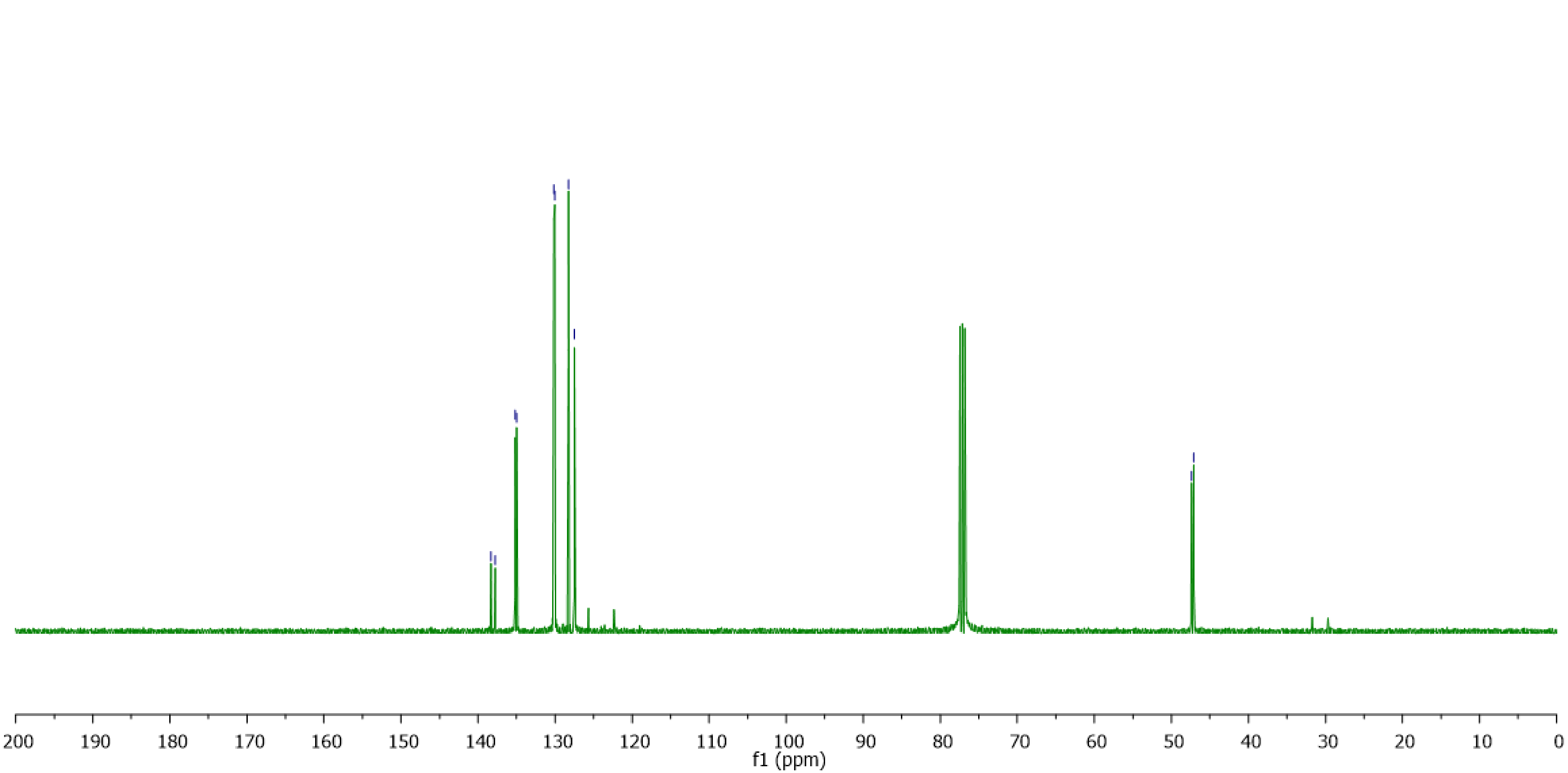

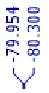

^{19}F {^{1}H} NMR spectrum
Solvent: $CDCl_3$
Spectrometer Frequency: 376 MHz

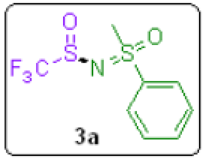

3a

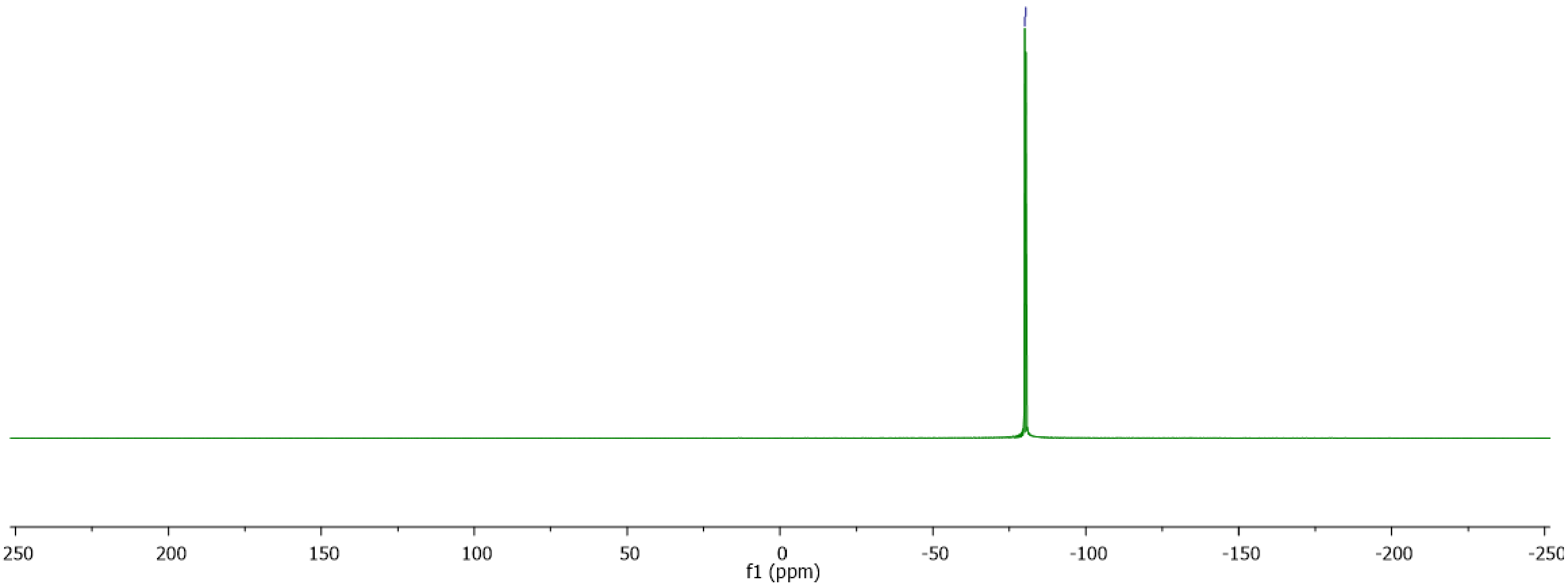

^{1}H NMR spectrum
Solvent: $CDCl_3$
Spectrometer Frequency: 400 MHz

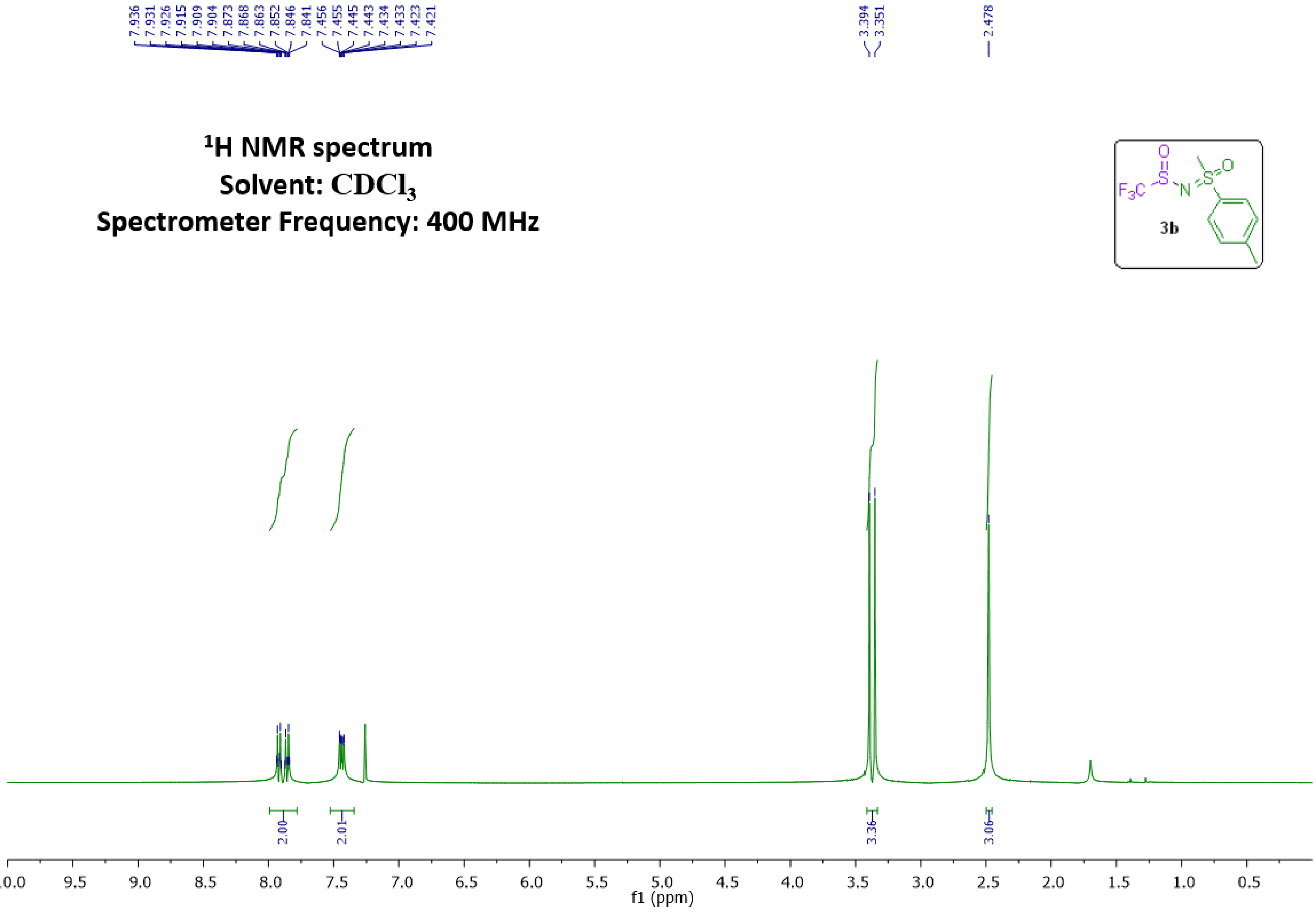

$^{13}C\ \{^{1}H\}$ NMR spectrum

Solvent: $CDCl_3$

Spectrometer Frequency: 100 MHz

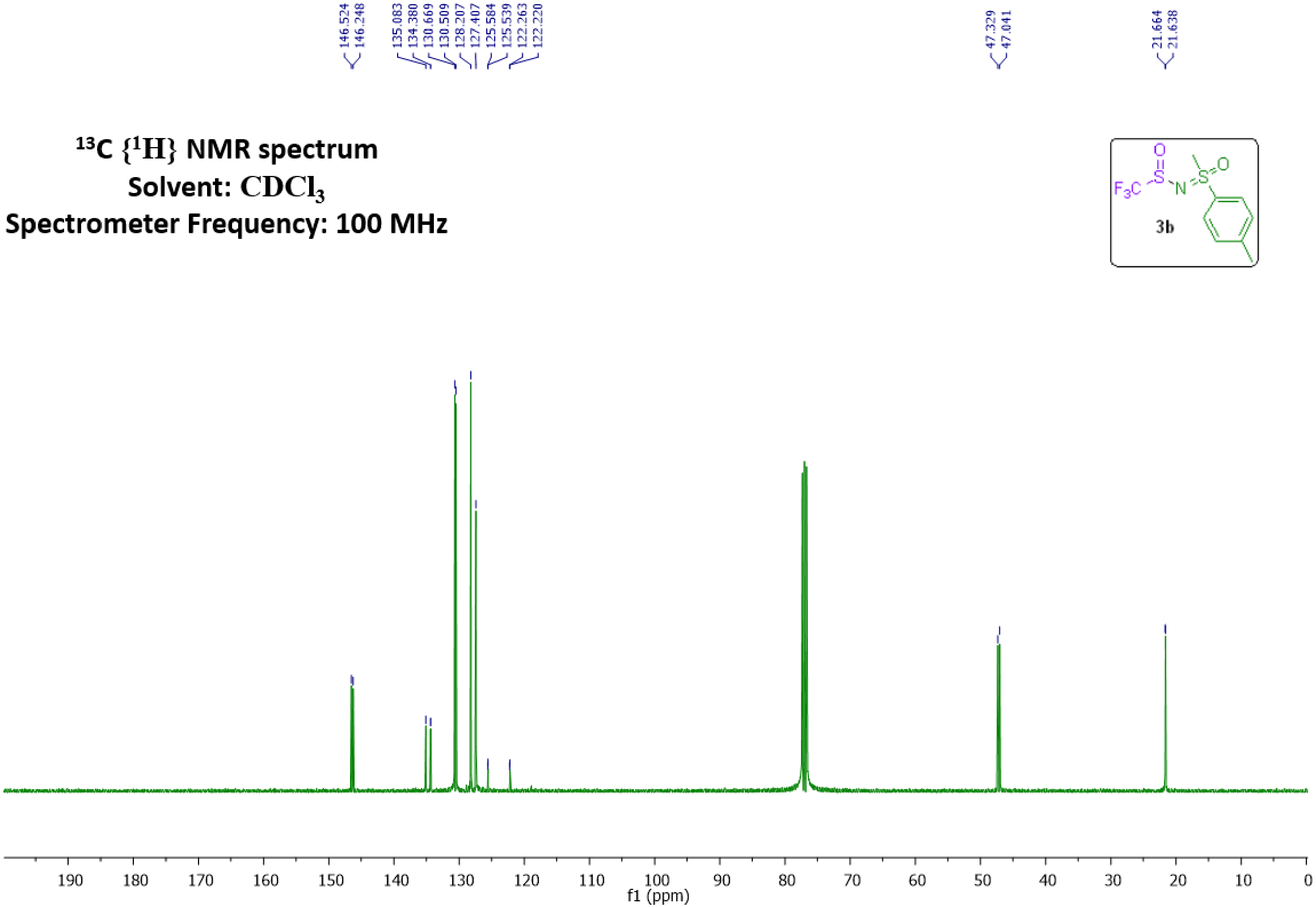

^{19}F {^{1}H} NMR spectrum
Solvent: $CDCl_3$
Spectrometer Frequency: 376 MHz

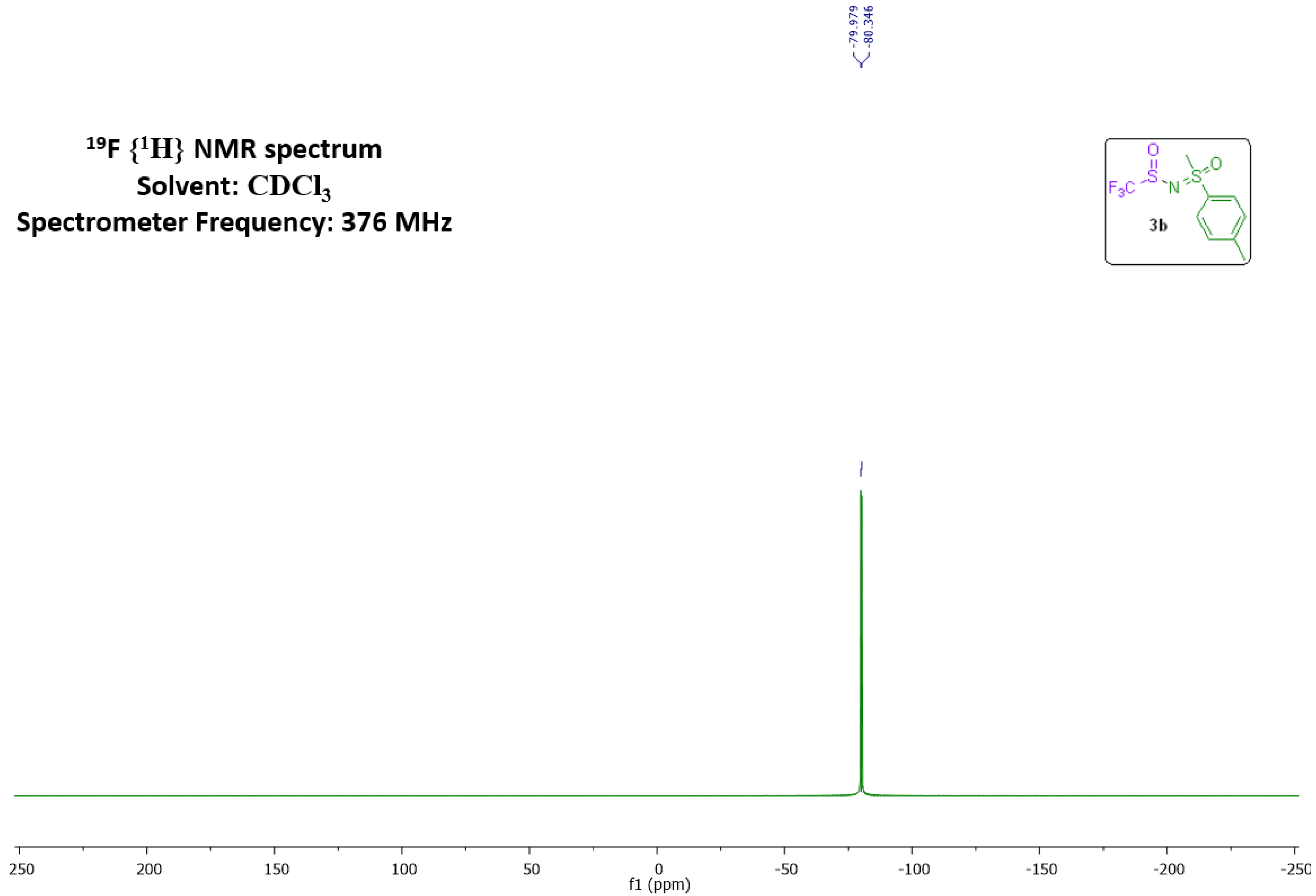

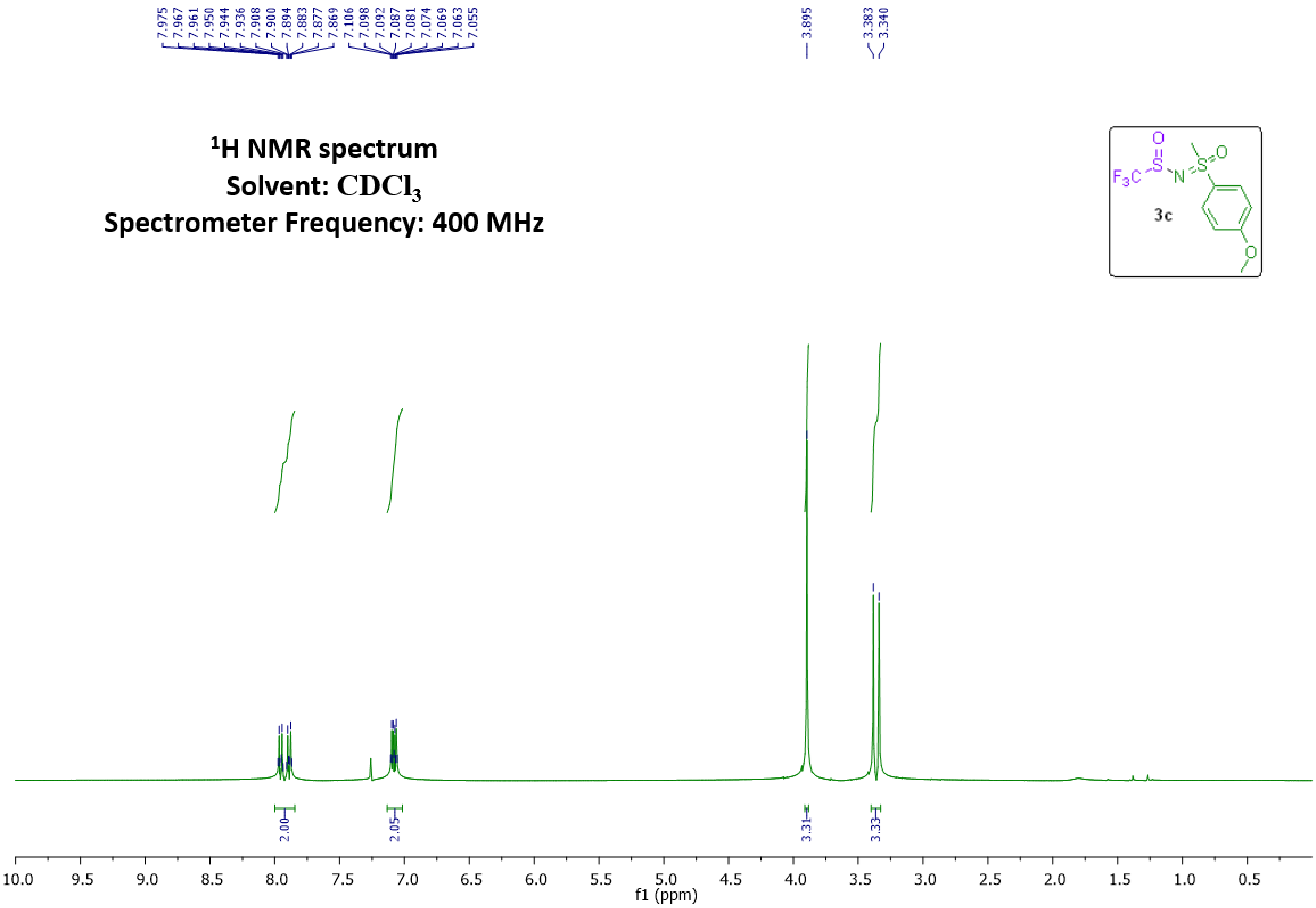

7.975
7.967
7.961
7.950
7.944
7.936
7.908
7.900
7.894
7.883
7.877
7.869
7.106
7.098
7.092
7.087
7.081
7.074
7.069
7.063
7.055
3.895
3.383
3.340
^{1}H NMR spectrum
Solvent: $CDCl_3$
Spectrometer Frequency: 400 MHz
2.00
2.05
3.31
3.33
10.0
9.5
9.0
8.5
8.0
7.5
7.0
6.5
6.0
5.5
5.0
4.5
4.0
3.5
3.0
2.5
2.0
1.5
1.0
0.5
f1 (ppm)
O
F3C
S
N
S
O
O

3c

^{13}C {^{1}H} NMR spectrum
Solvent: $CDCl_3$
Spectrometer Frequency: 100 MHz

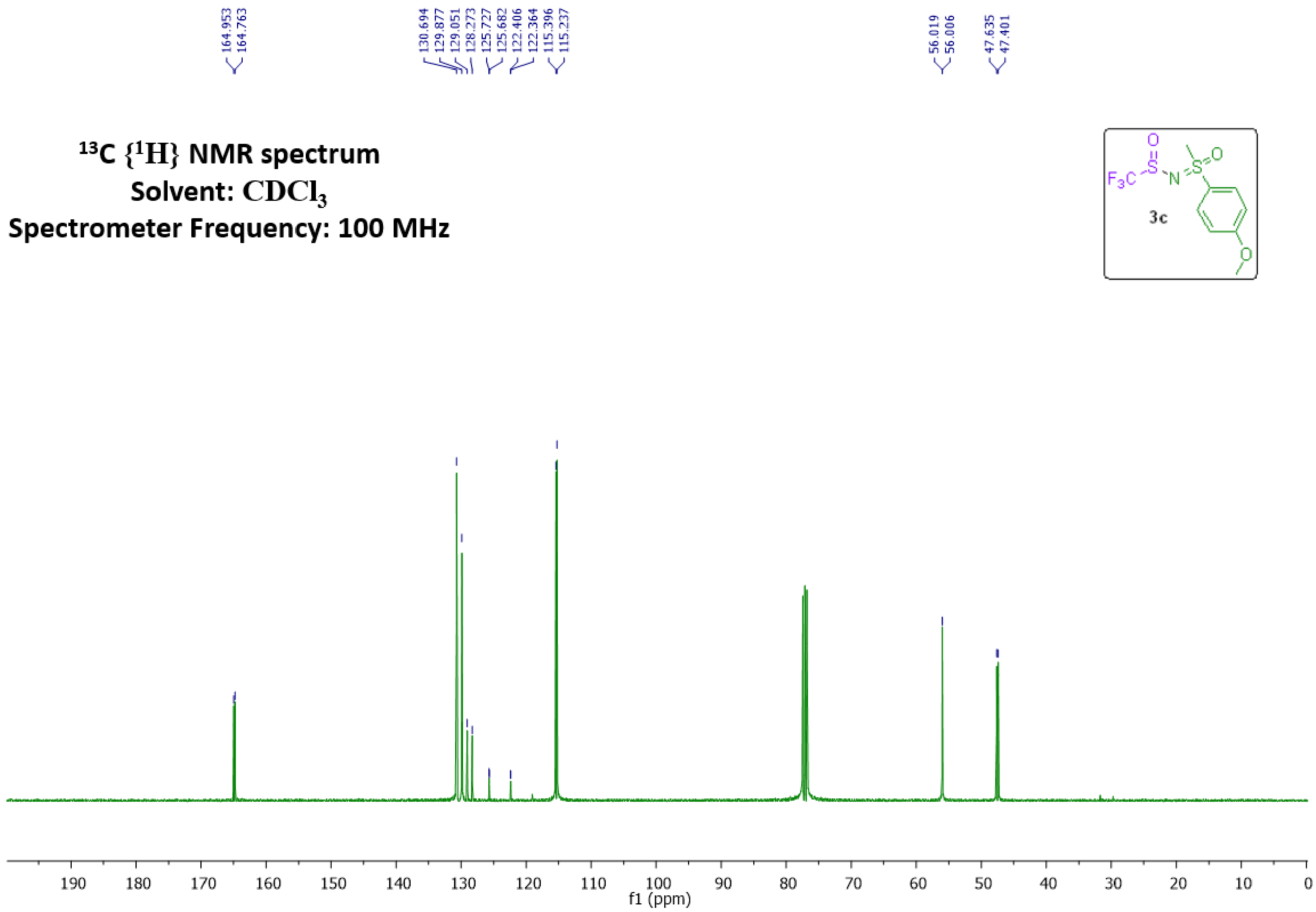

^{19}F {^{1}H} NMR spectrum
Solvent: $CDCl_3$
Spectrometer Frequency: 376 MHz

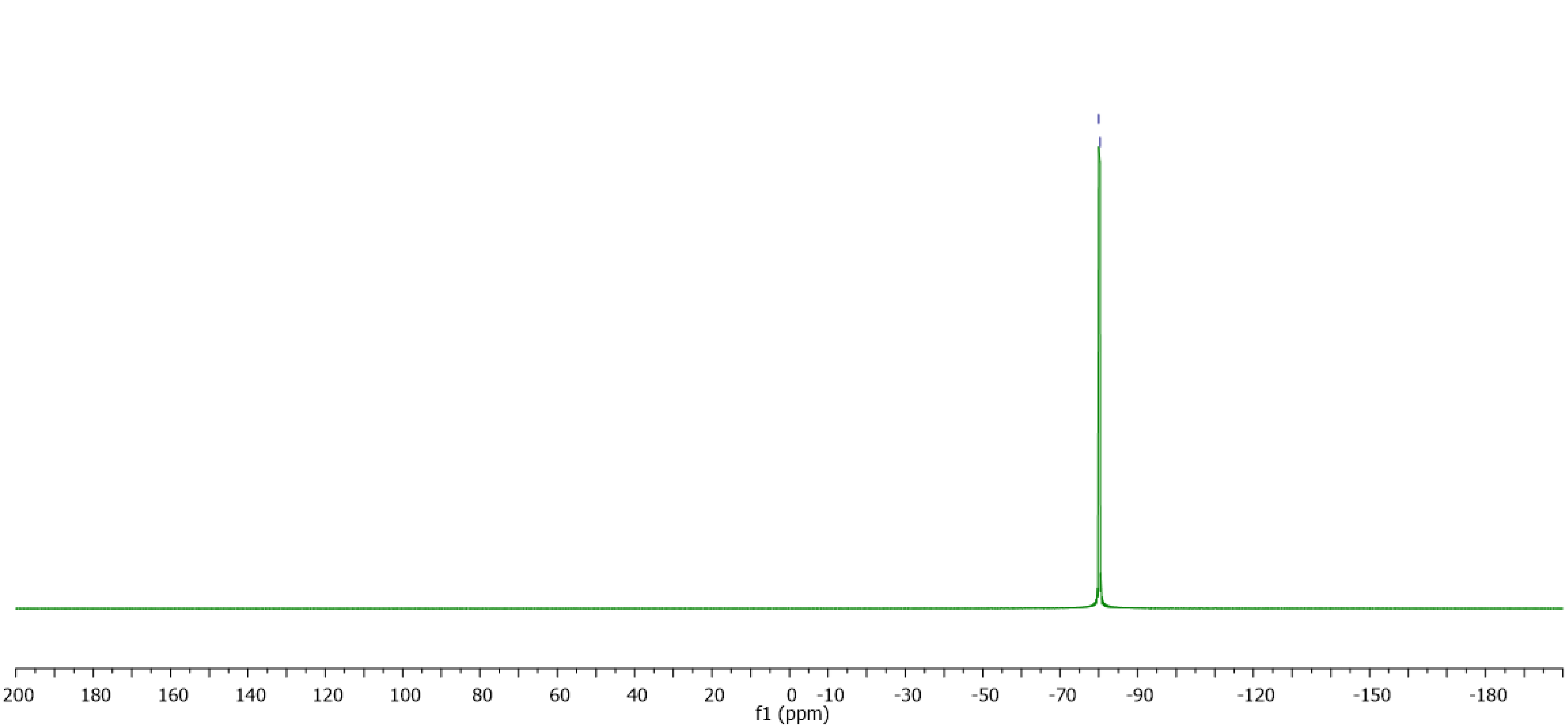

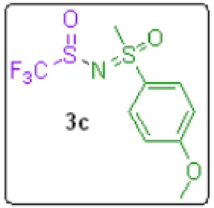

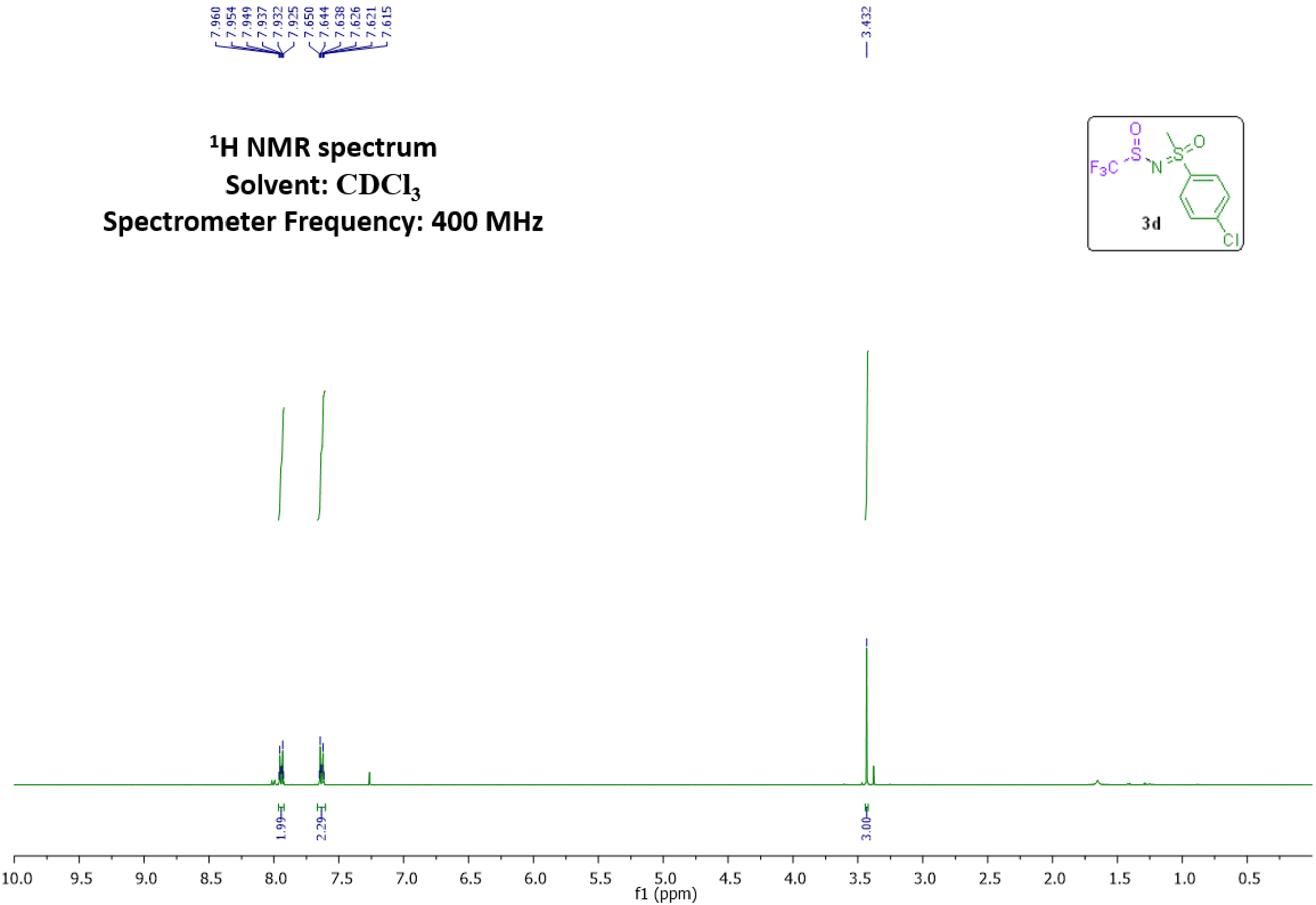
7.960
7.954
7.949
7.937
7.932
7.925
7.650
7.644
7.638
7.626
7.621
7.615
3.432
1H NMR spectrum
Solvent: CDCl3
Spectrometer Frequency: 400 MHz
F3C
O
S
N
S
O
Cl
3d
1.99
2.29
3.00
10.0
9.5
9.0
8.5
8.0
7.5
7.0
6.5
6.0
5.5
5.0
4.5
4.0
3.5
3.0
2.5
2.0
1.5
1.0
0.5
f1 (ppm)

^{13}C {^{1}H} NMR spectrum
Solvent: $CDCl_3$
Spectrometer Frequency: 100 MHz

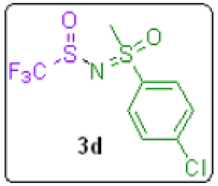

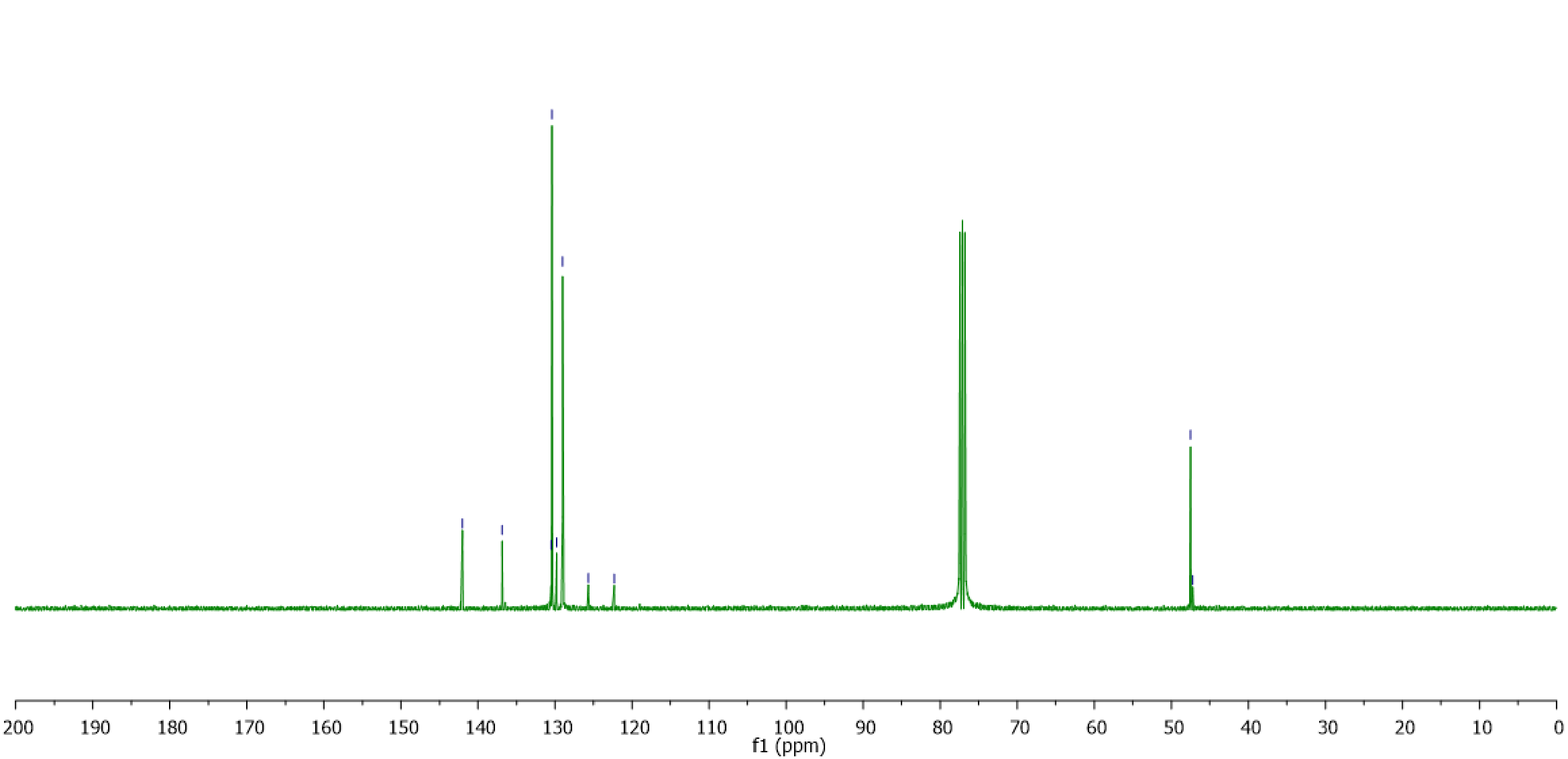

^{19}F {^1H} NMR spectrum
Solvent: $CDCl_3$
Spectrometer Frequency: 376 MHz

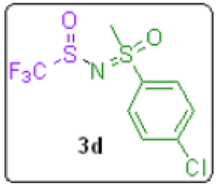

3d

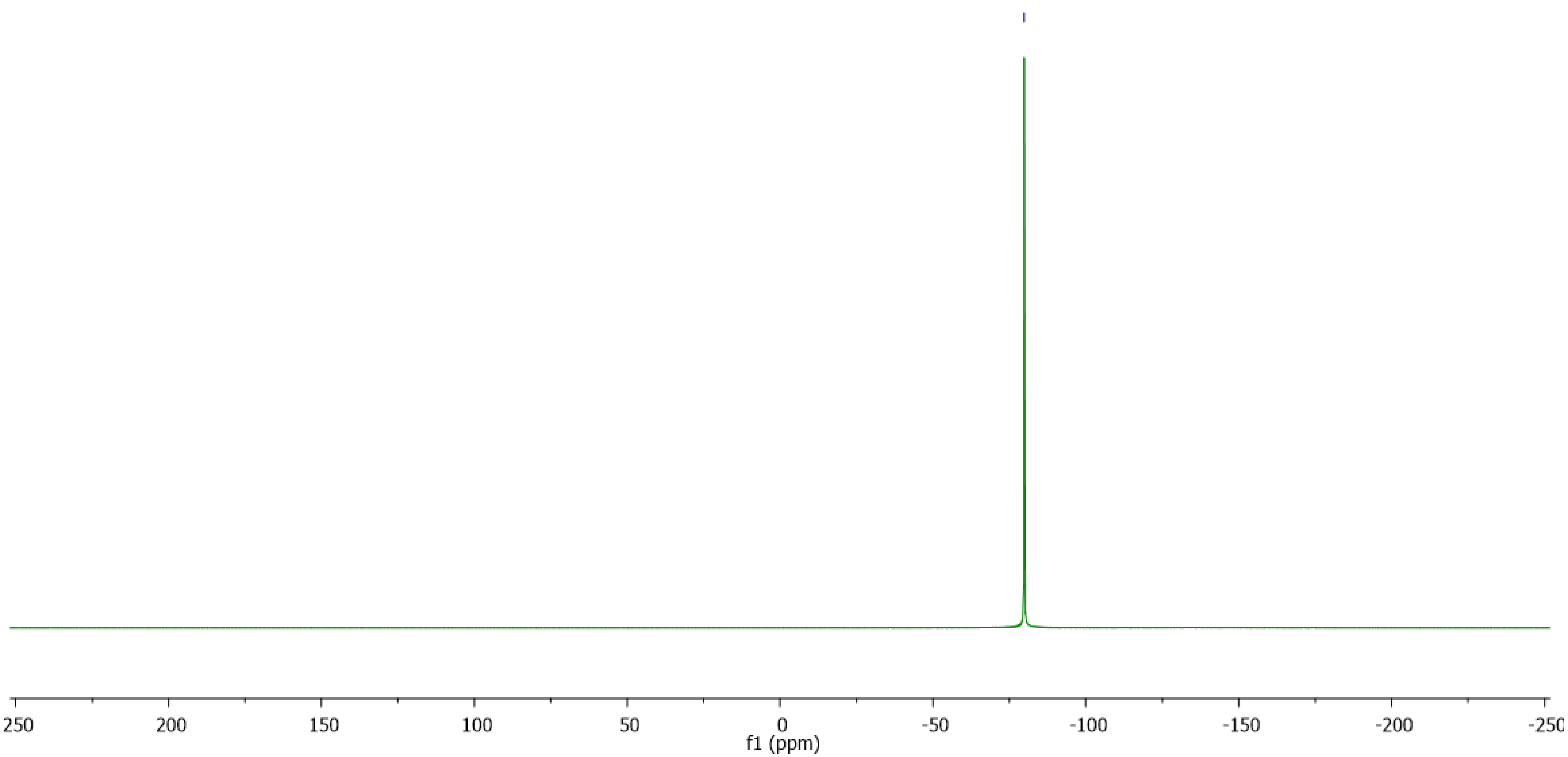

^{1}H NMR spectrum
Solvent: $CDCl_3$
Spectrometer Frequency: 400 MHz

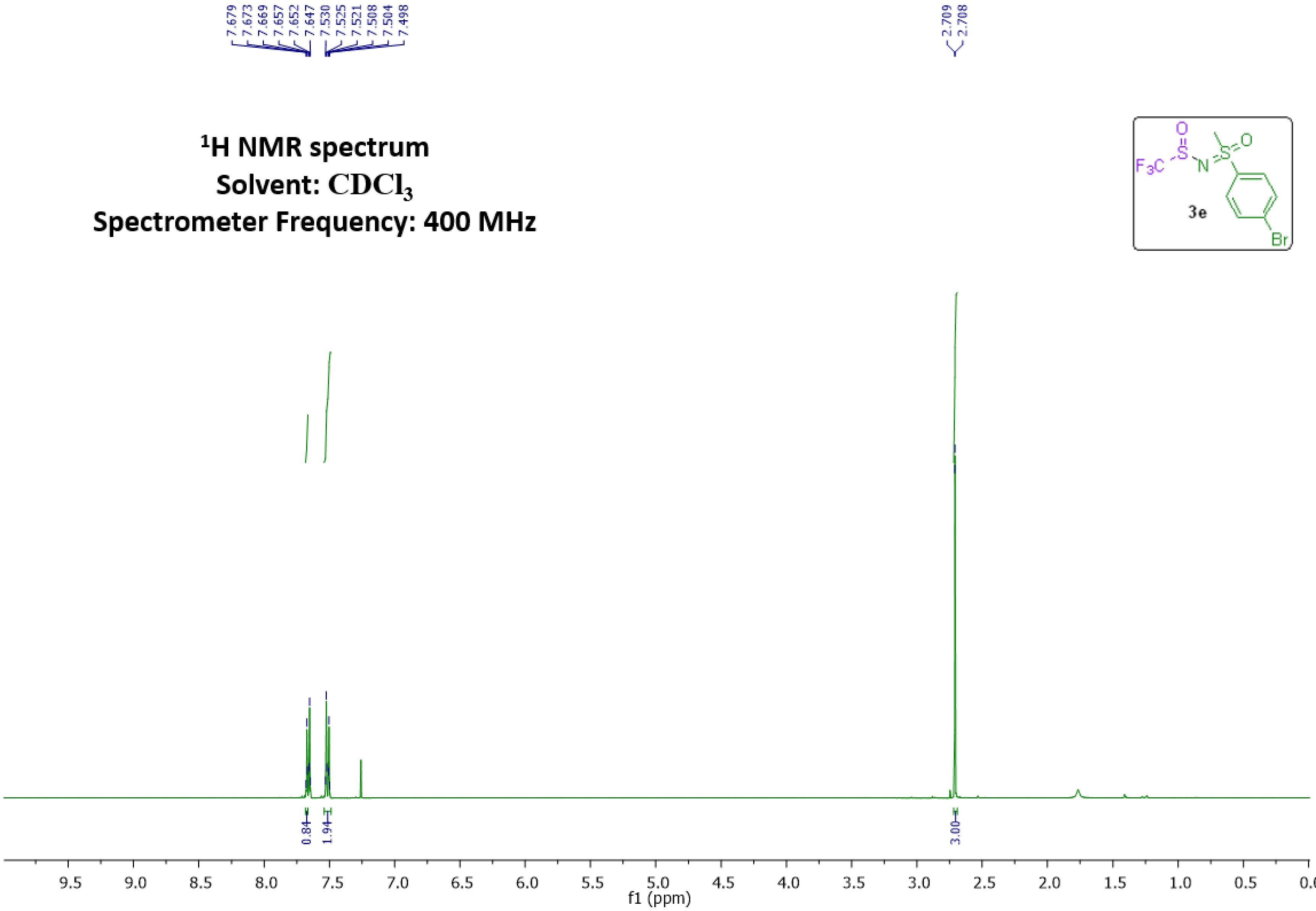

^{13}C {^{1}H} NMR spectrum
Solvent: $CDCl_3$
Spectrometer Frequency: 100 MHz

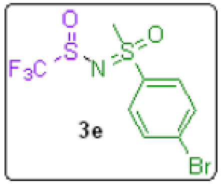

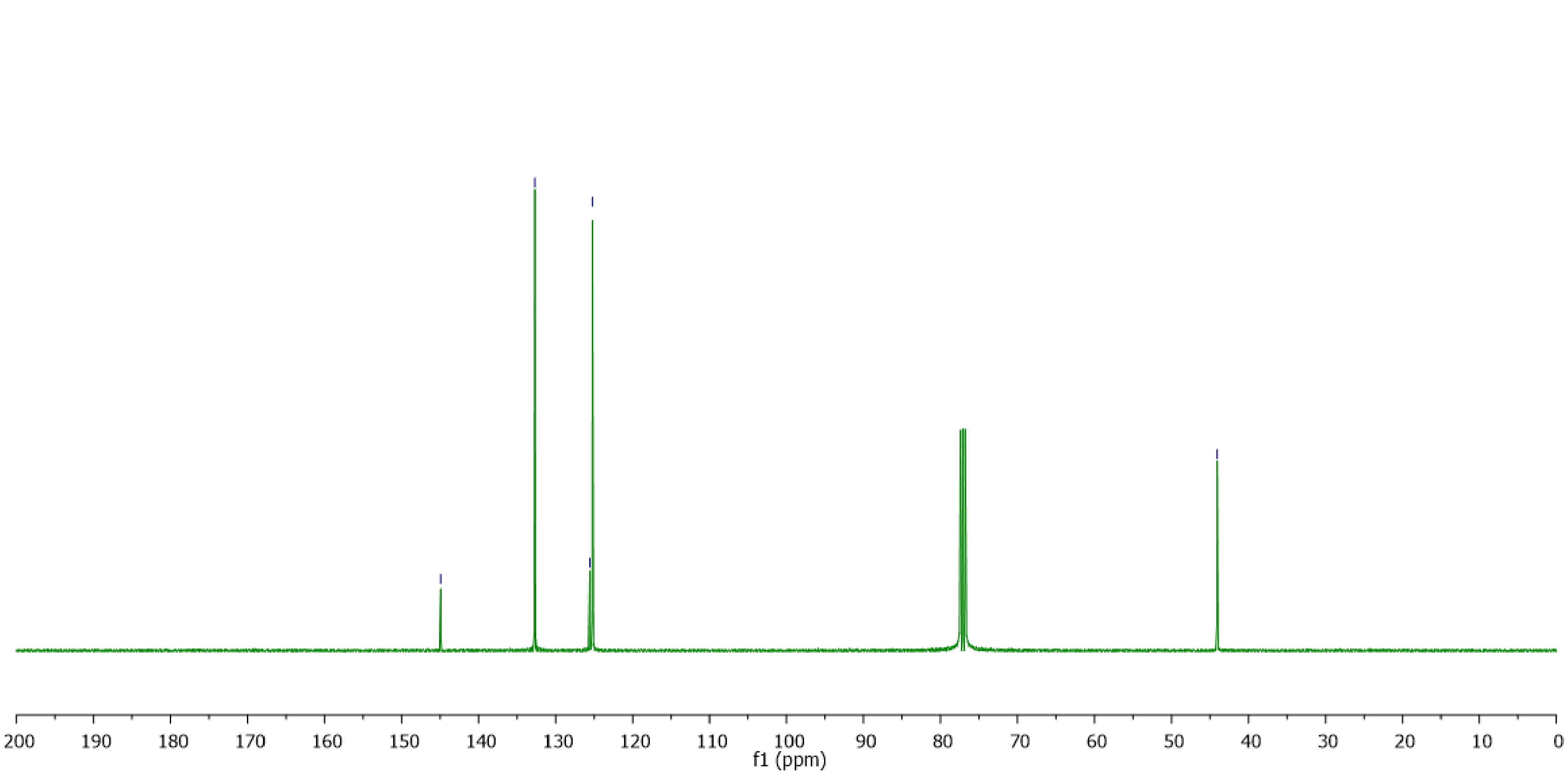

^{19}F {^{1}H} NMR spectrum
Solvent: $CDCl_3$
Spectrometer Frequency: 376 MHz

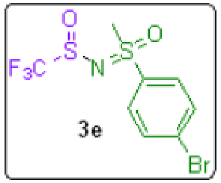

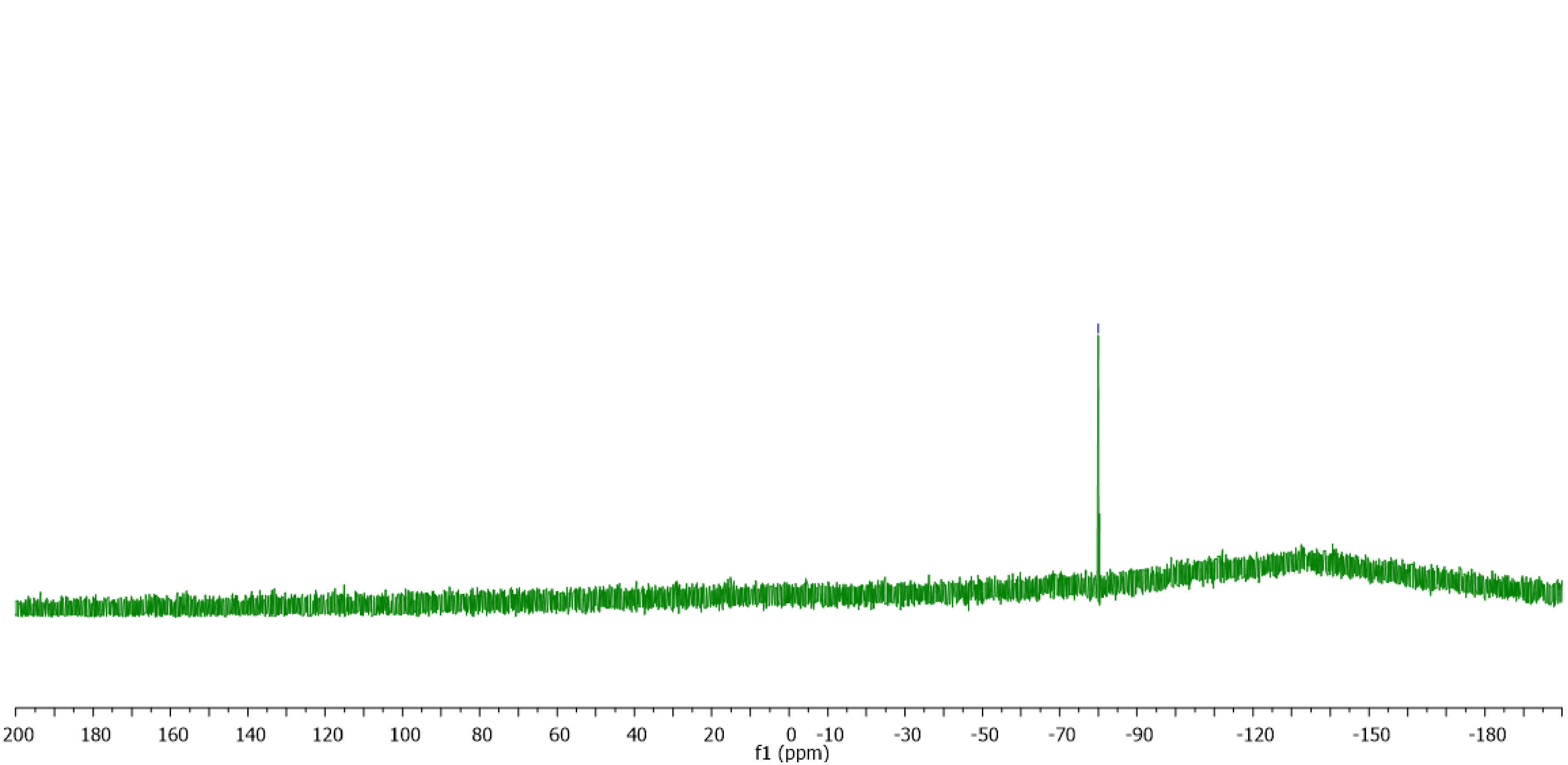

^{1}H NMR spectrum
Solvent: $CDCl_3$
Spectrometer Frequency: 400 MHz

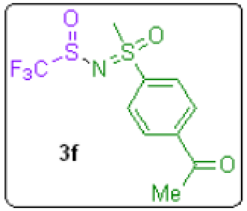

3f

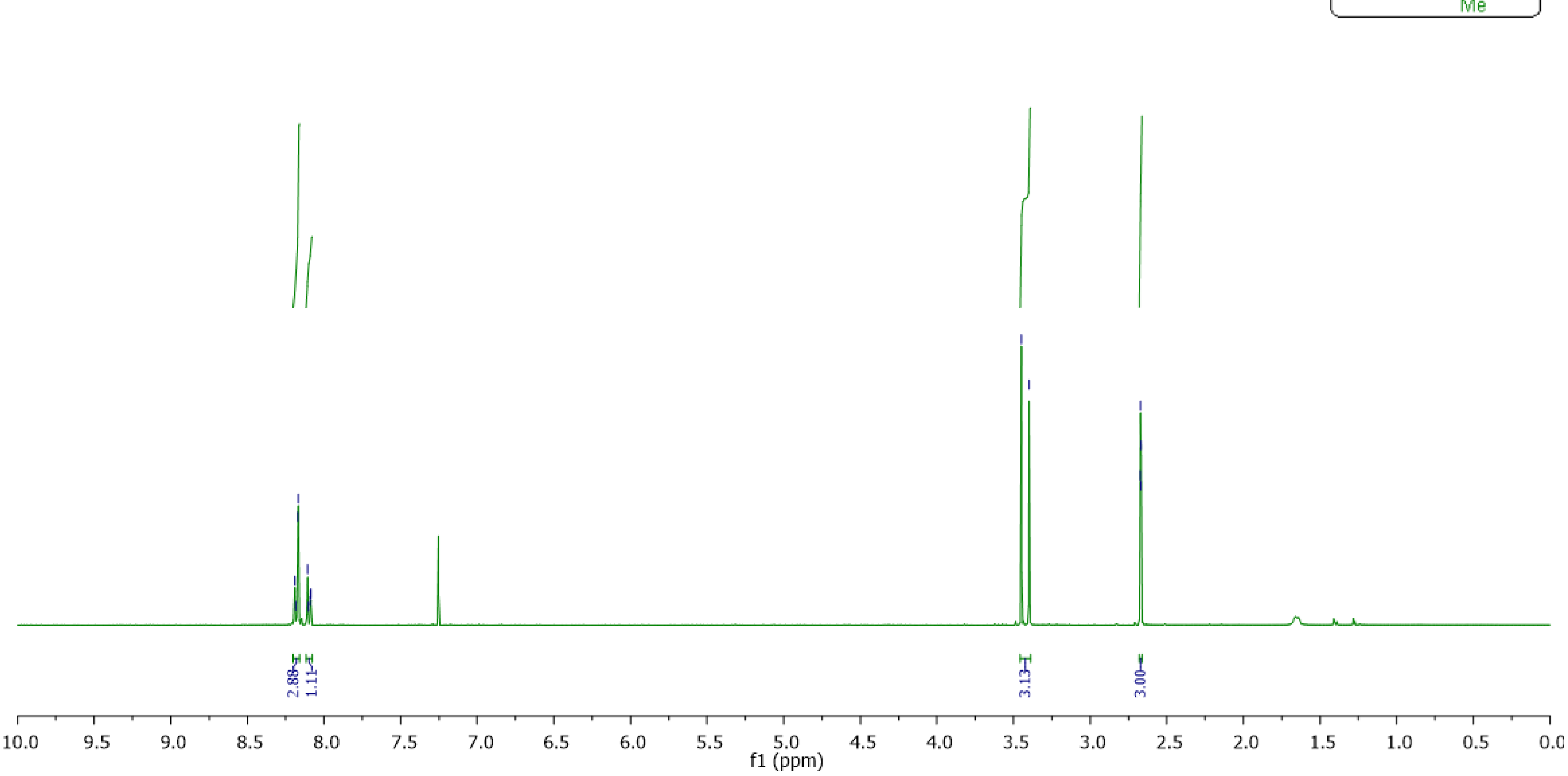

^{13}C {^{1}H} NMR spectrum
Solvent: $CDCl_3$
Spectrometer Frequency: 100 MHz

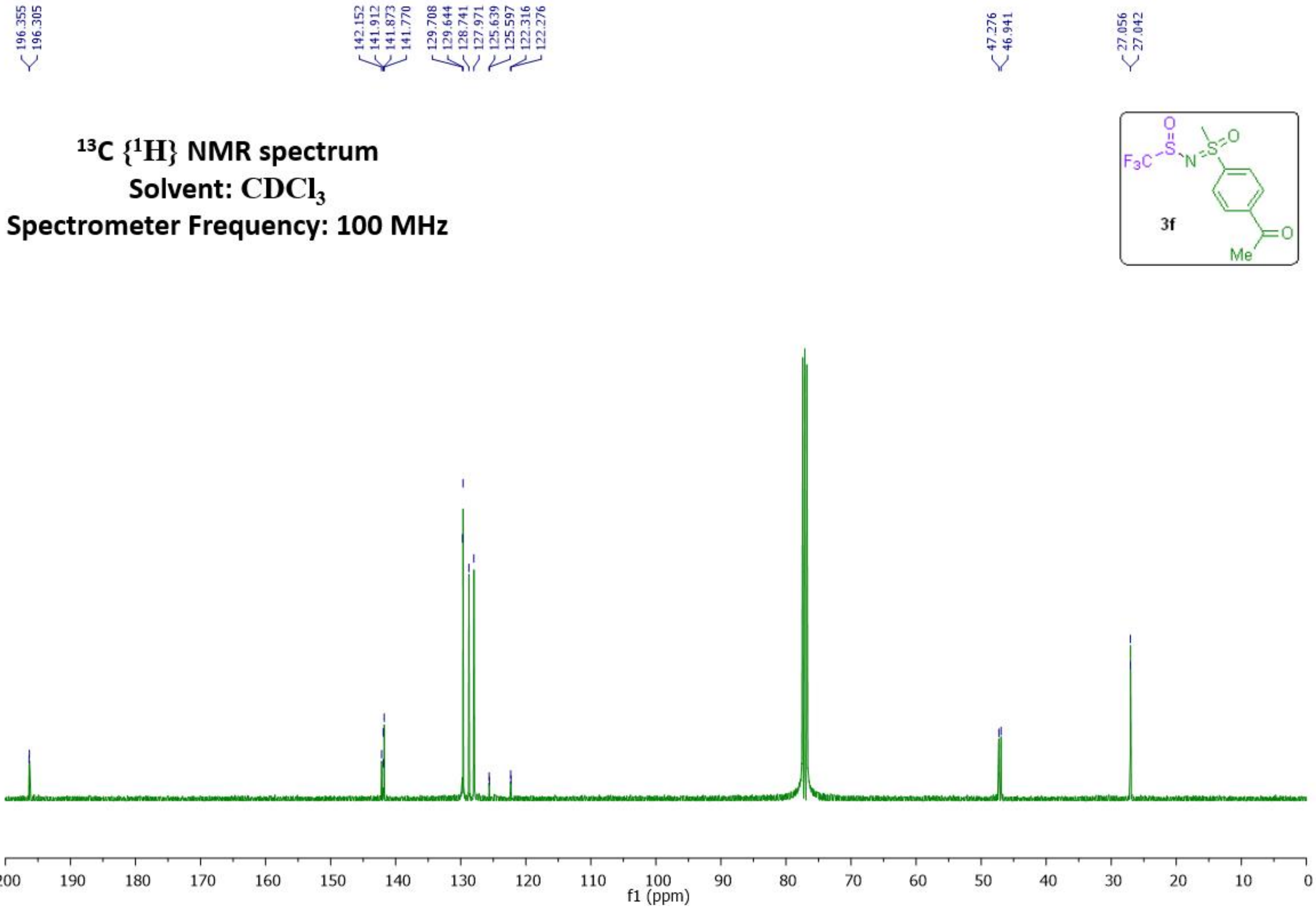

^{19}F {^{1}H} NMR spectrum
Solvent: $CDCl_3$
Spectrometer Frequency: 376 MHz

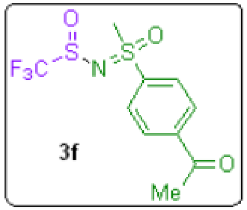

3f

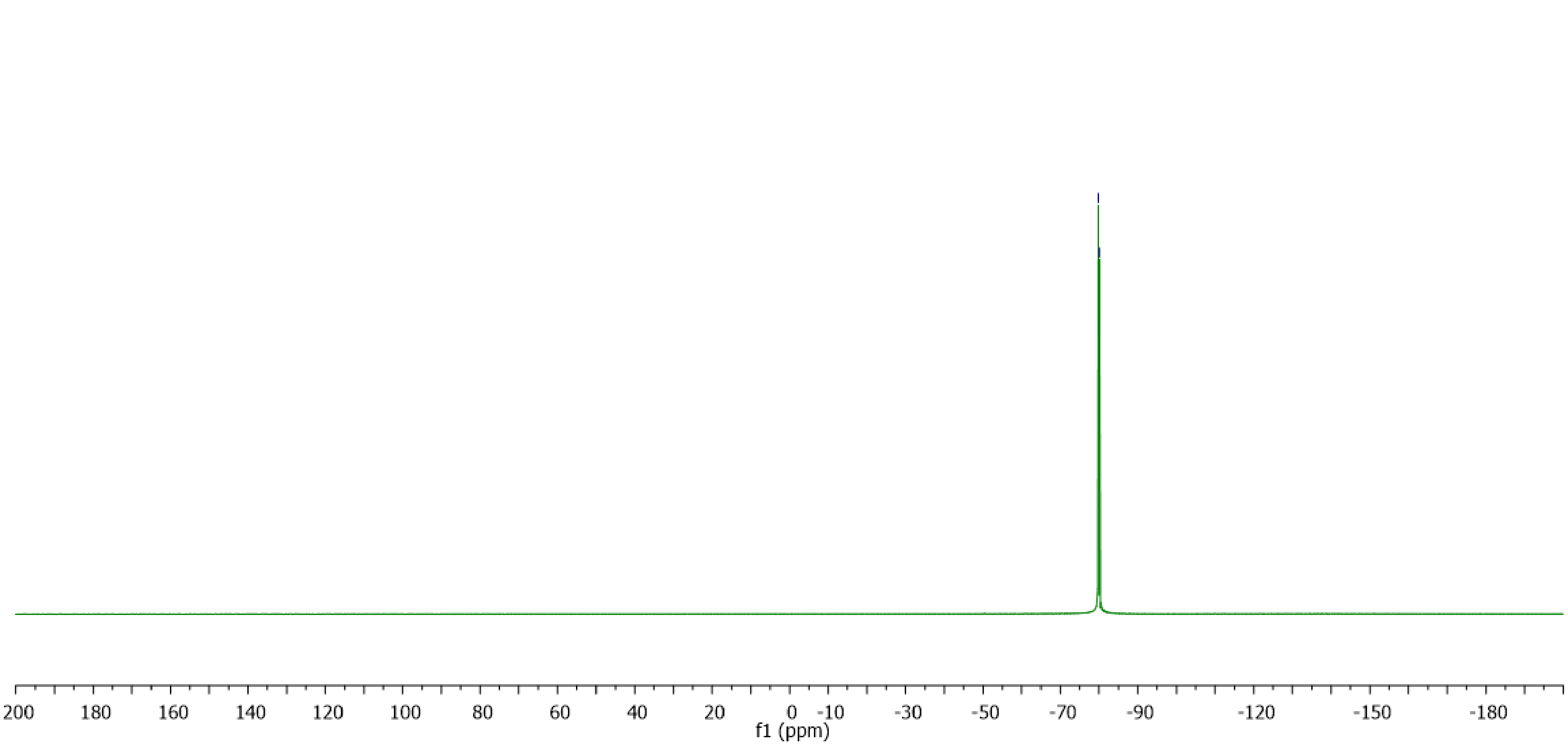

^{1}H NMR spectrum
Solvent: $CDCl_3$
Spectrometer Frequency: 400 MHz

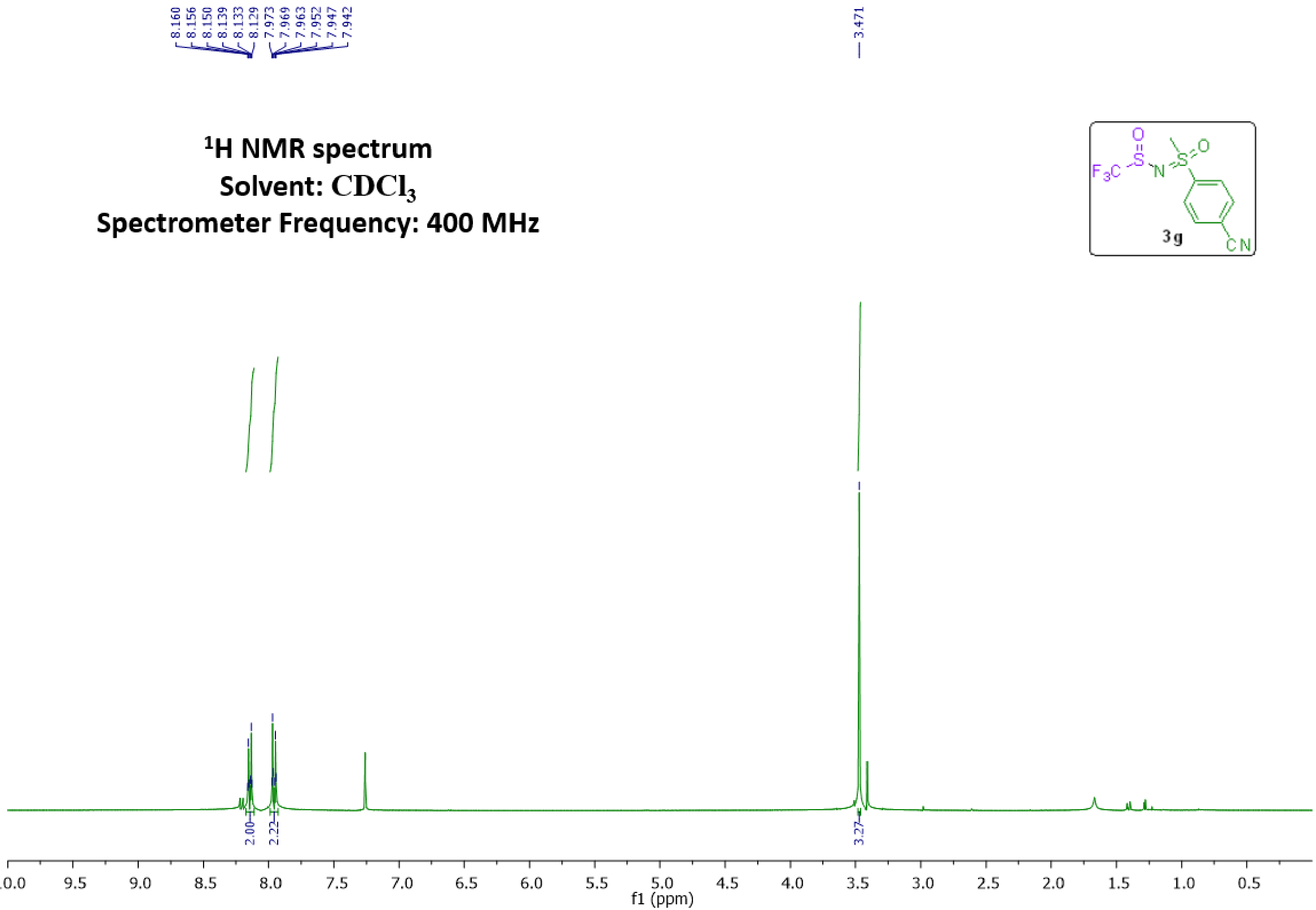

^{13}C {^{1}H} NMR spectrum
Solvent: $CDCl_3$
Spectrometer Frequency: 100 MHz

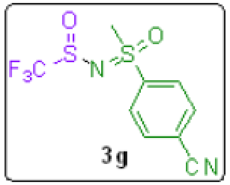

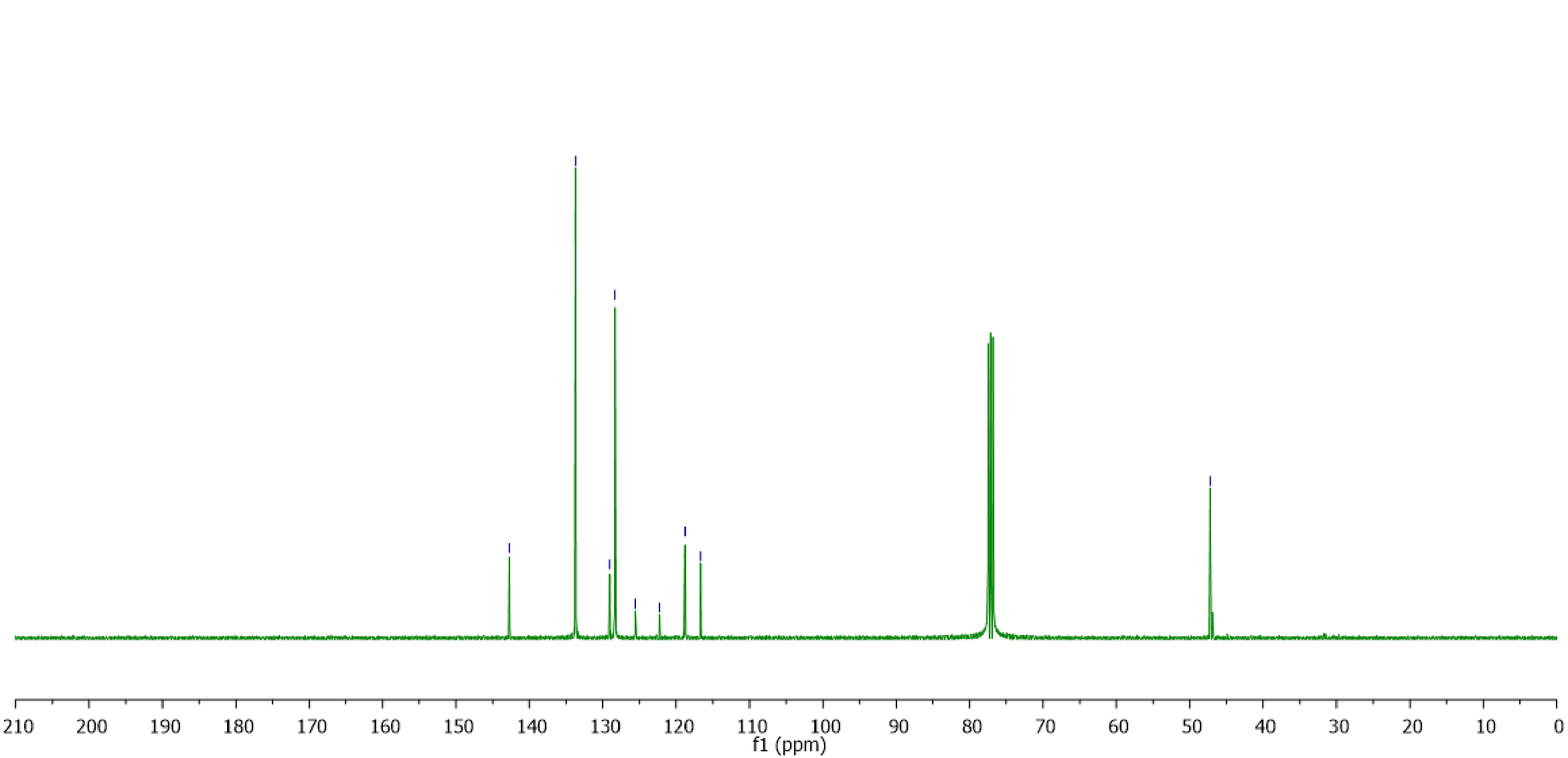

^{19}F {^1H} NMR spectrum
Solvent: $CDCl_3$
Spectrometer Frequency: 376 MHz

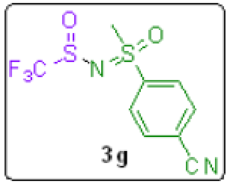

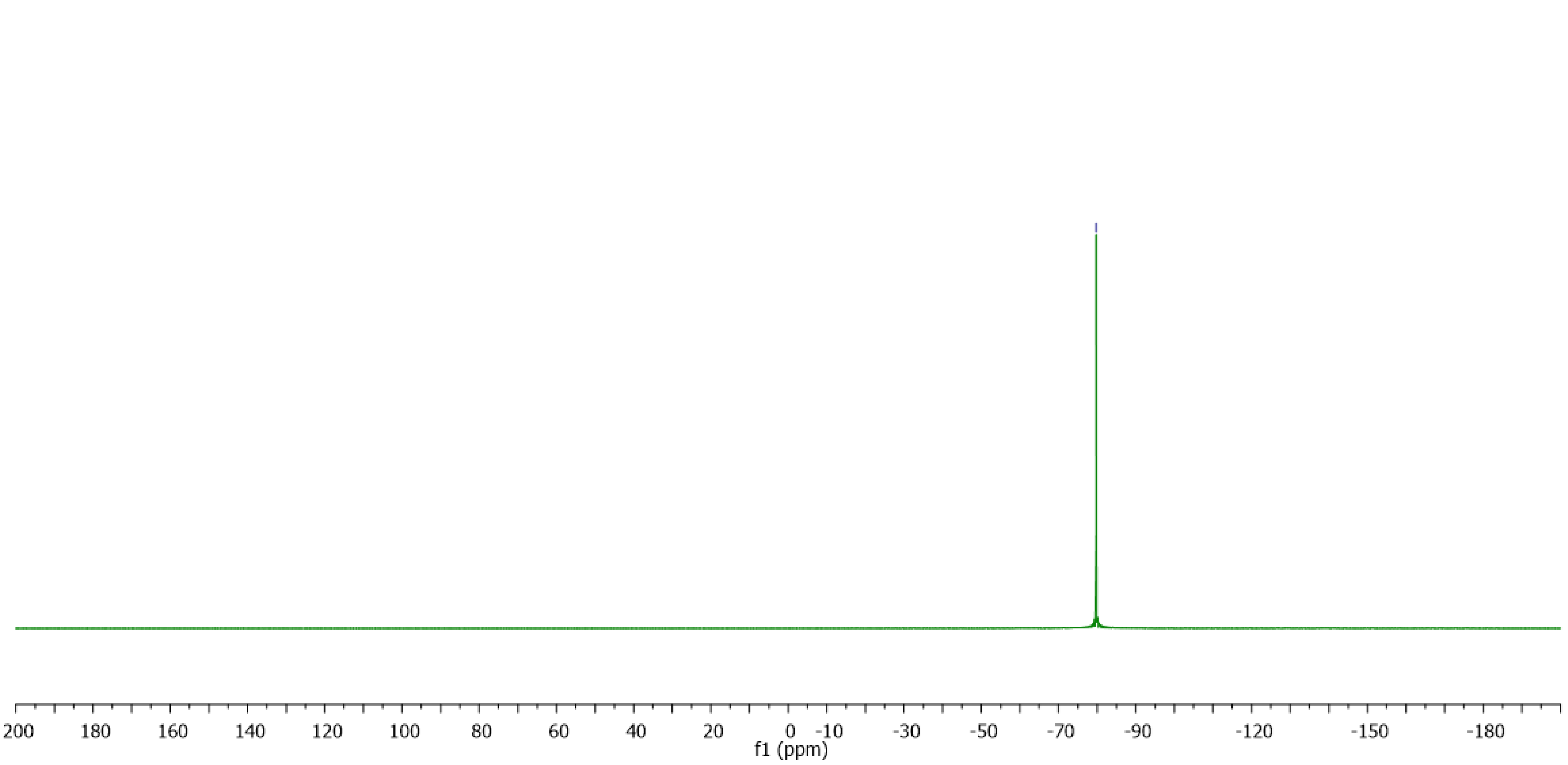

^{1}H NMR spectrum
Solvent: $CDCl_3$
Spectrometer Frequency: 400 MHz

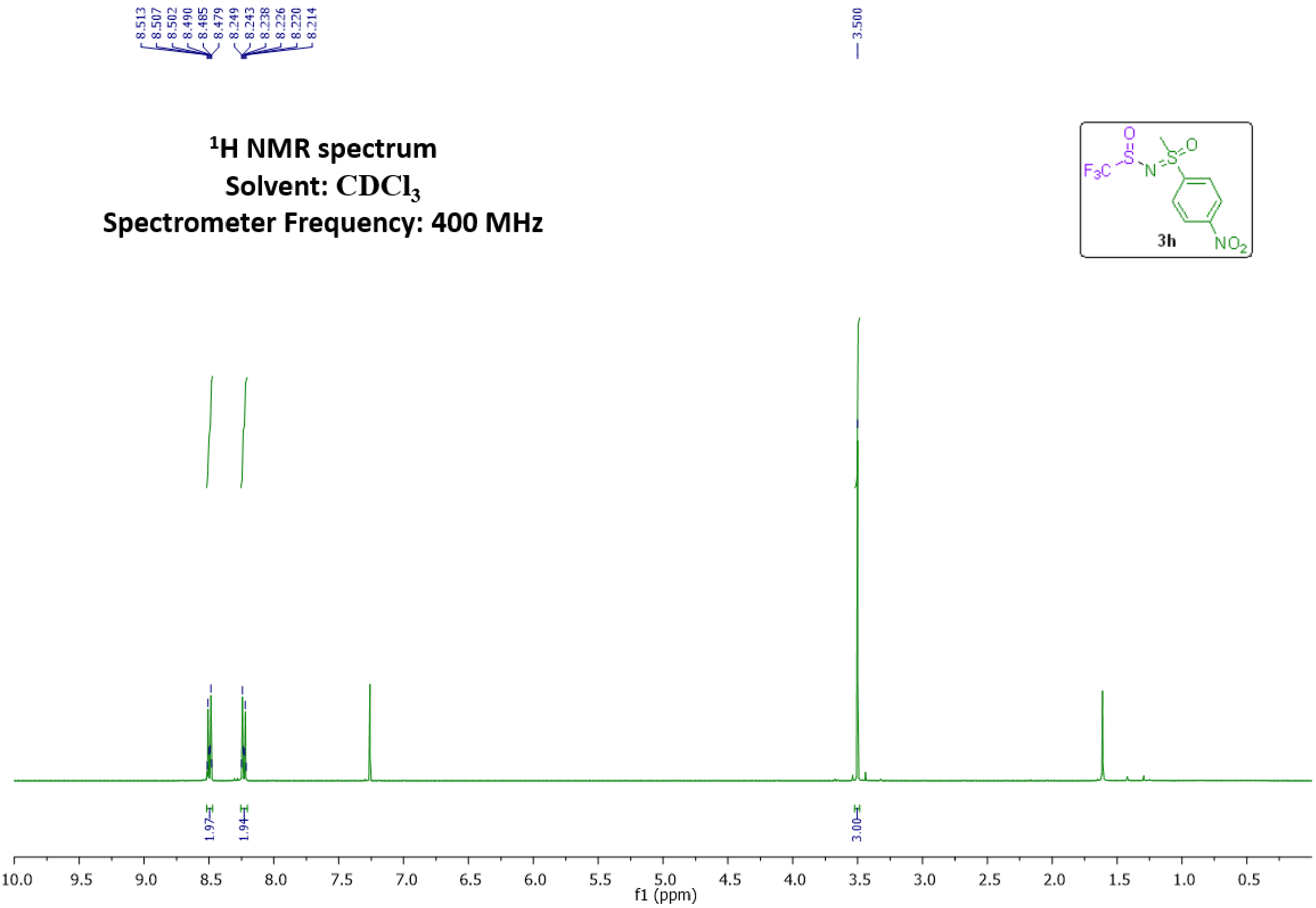

^{13}C {^{1}H} NMR spectrum
Solvent: $CDCl_3$
Spectrometer Frequency: 100 MHz

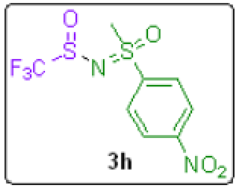

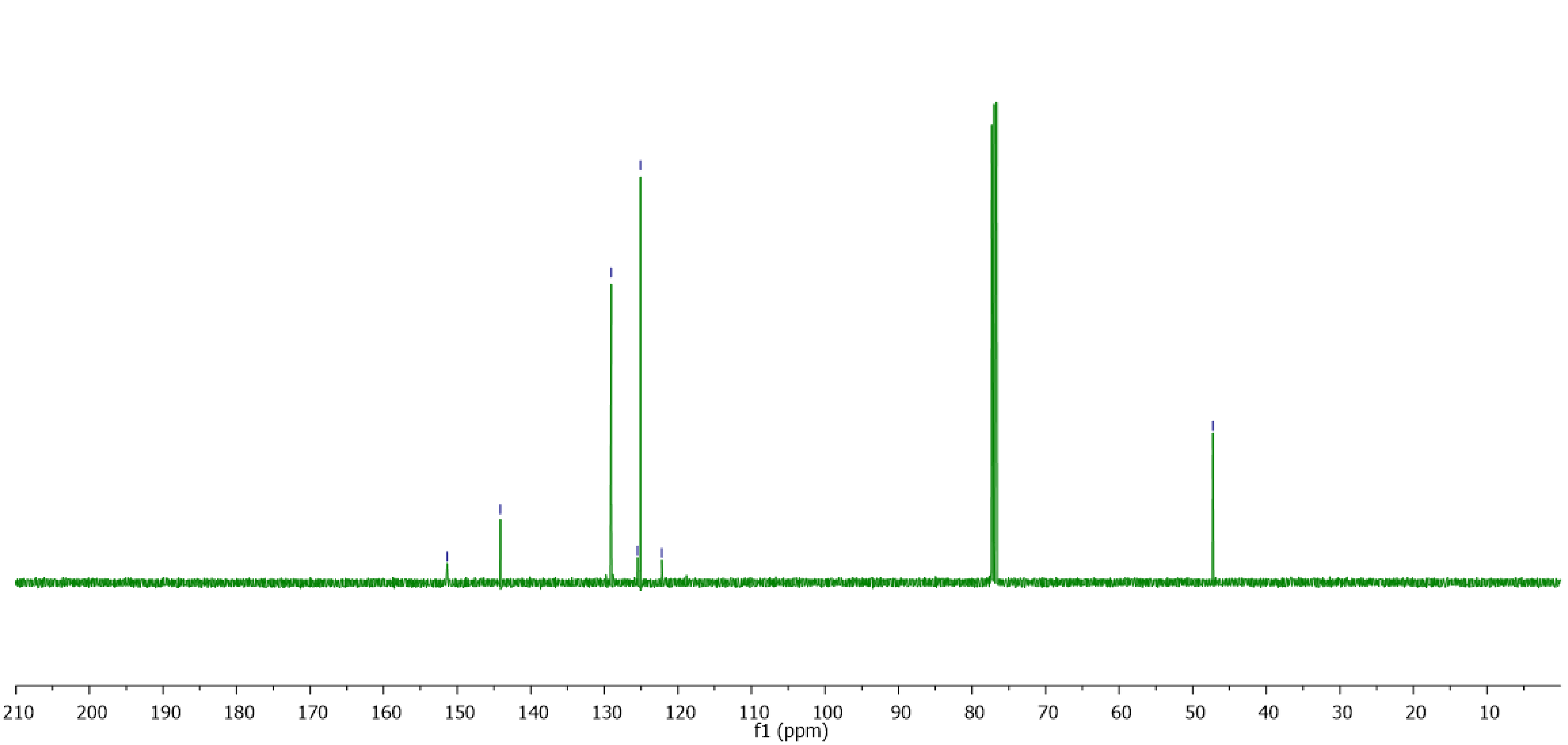

$^{19}F\ \{^1H\}$ NMR spectrum
Solvent: $CDCl_3$
Spectrometer Frequency: 376 MHz

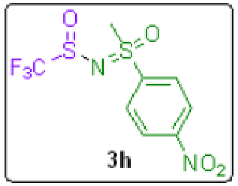

3h

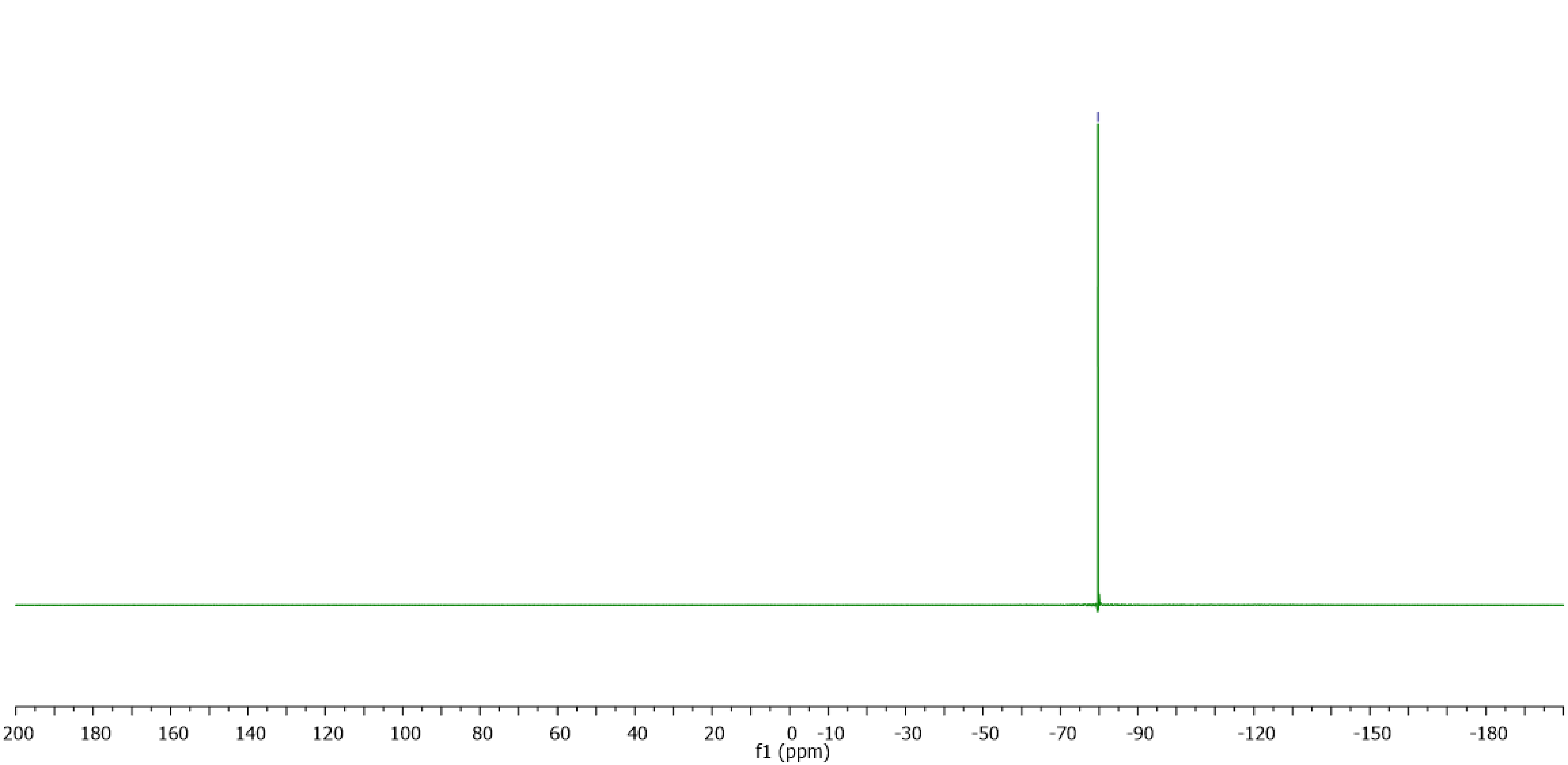

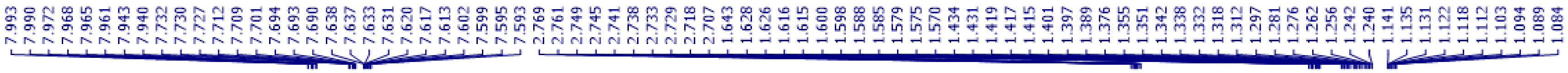

^{1}H NMR spectrum
Solvent: $CDCl_3$
Spectrometer Frequency: 400 MHz

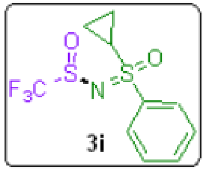

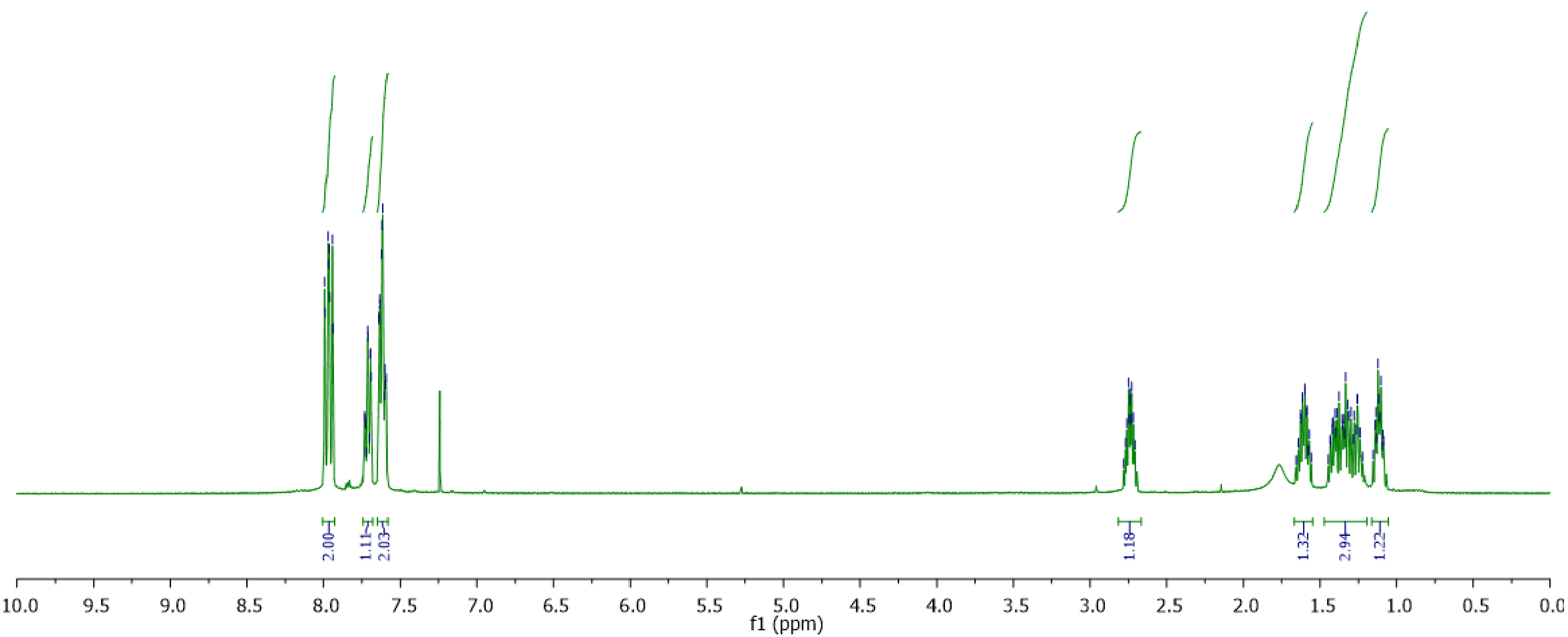

^{13}C {^{1}H} NMR spectrum
Solvent: $CDCl_3$
Spectrometer Frequency: 100 MHz

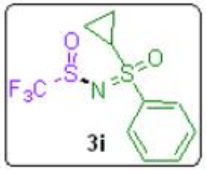

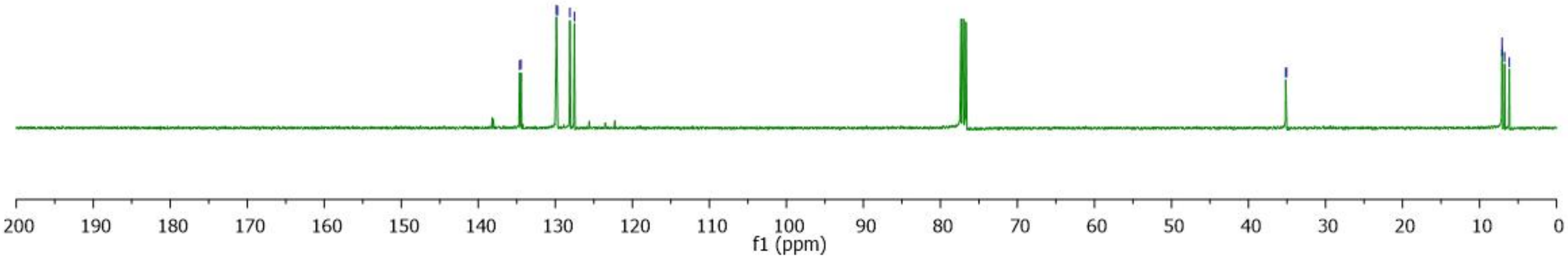

-80.289
-80.502

^{19}F {^{1}H} NMR spectrum

Solvent: $CDCl_3$

Spectrometer Frequency: 376 MHz

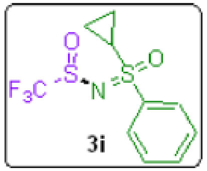

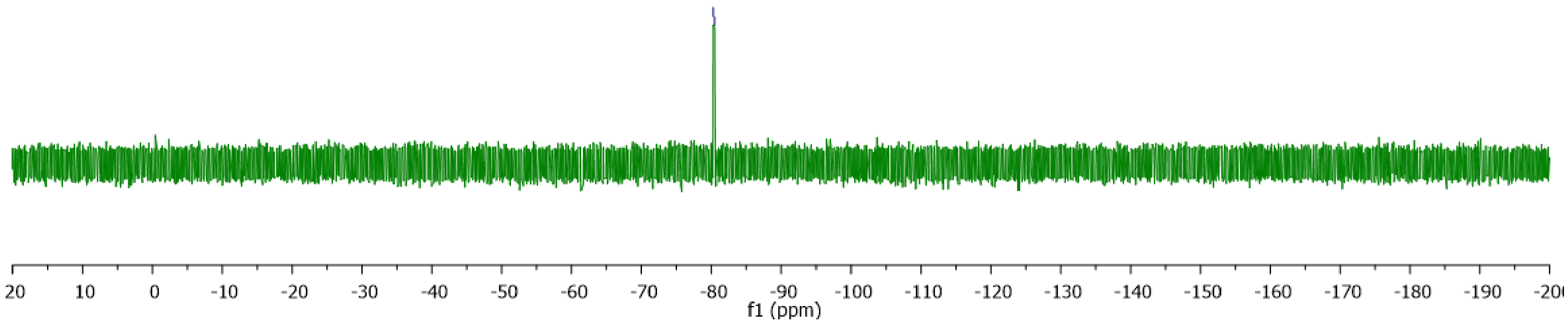

^{1}H NMR spectrum
Solvent: DMSO-d_6
Spectrometer Frequency: 400 MHz

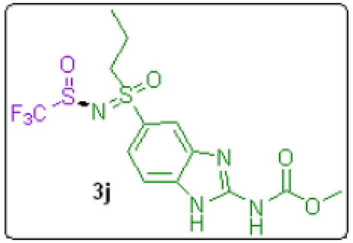

3j

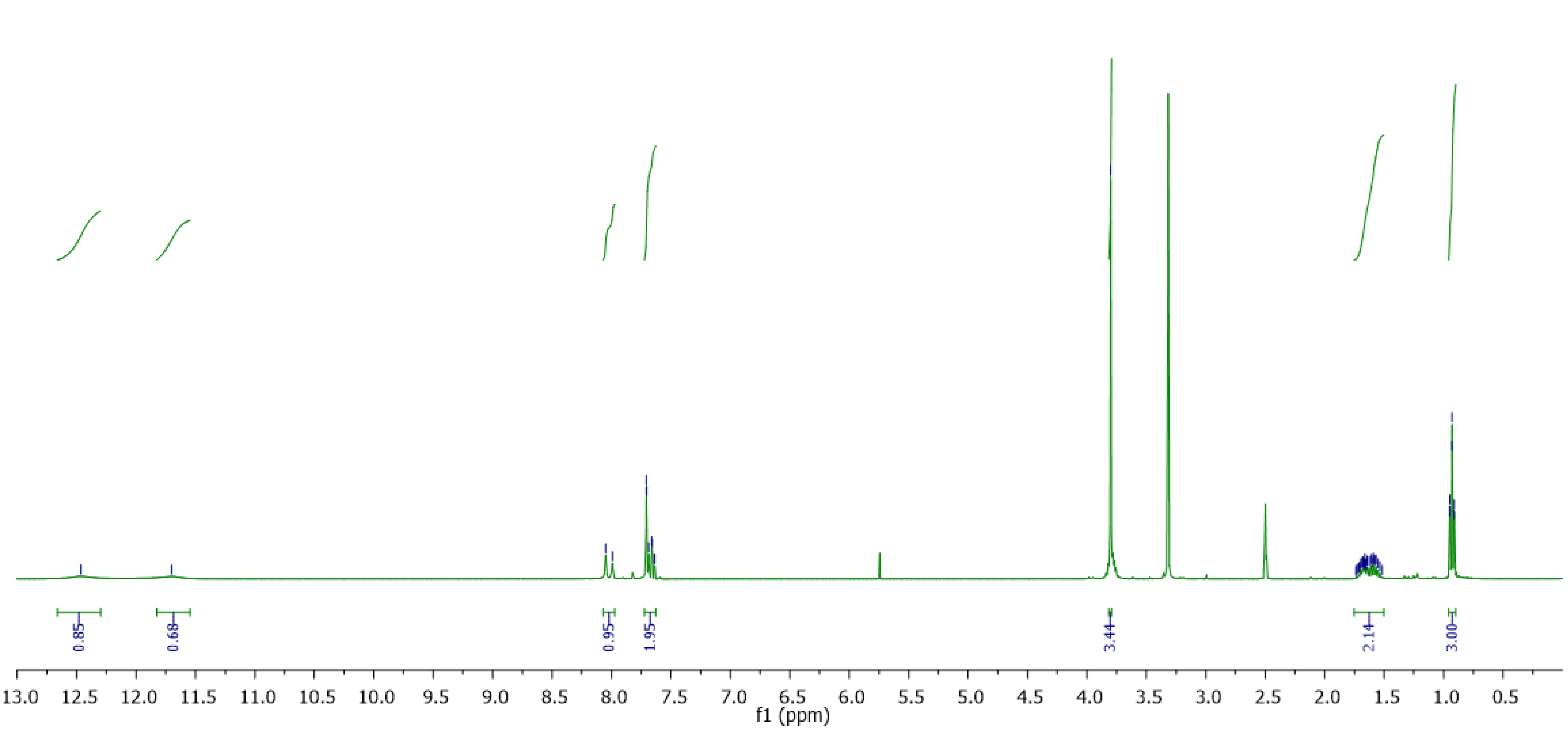

$^{13}C\ \{^{1}H\}$ NMR spectrum
Solvent: DMSO-d_6
Spectrometer Frequency: 100 MHz

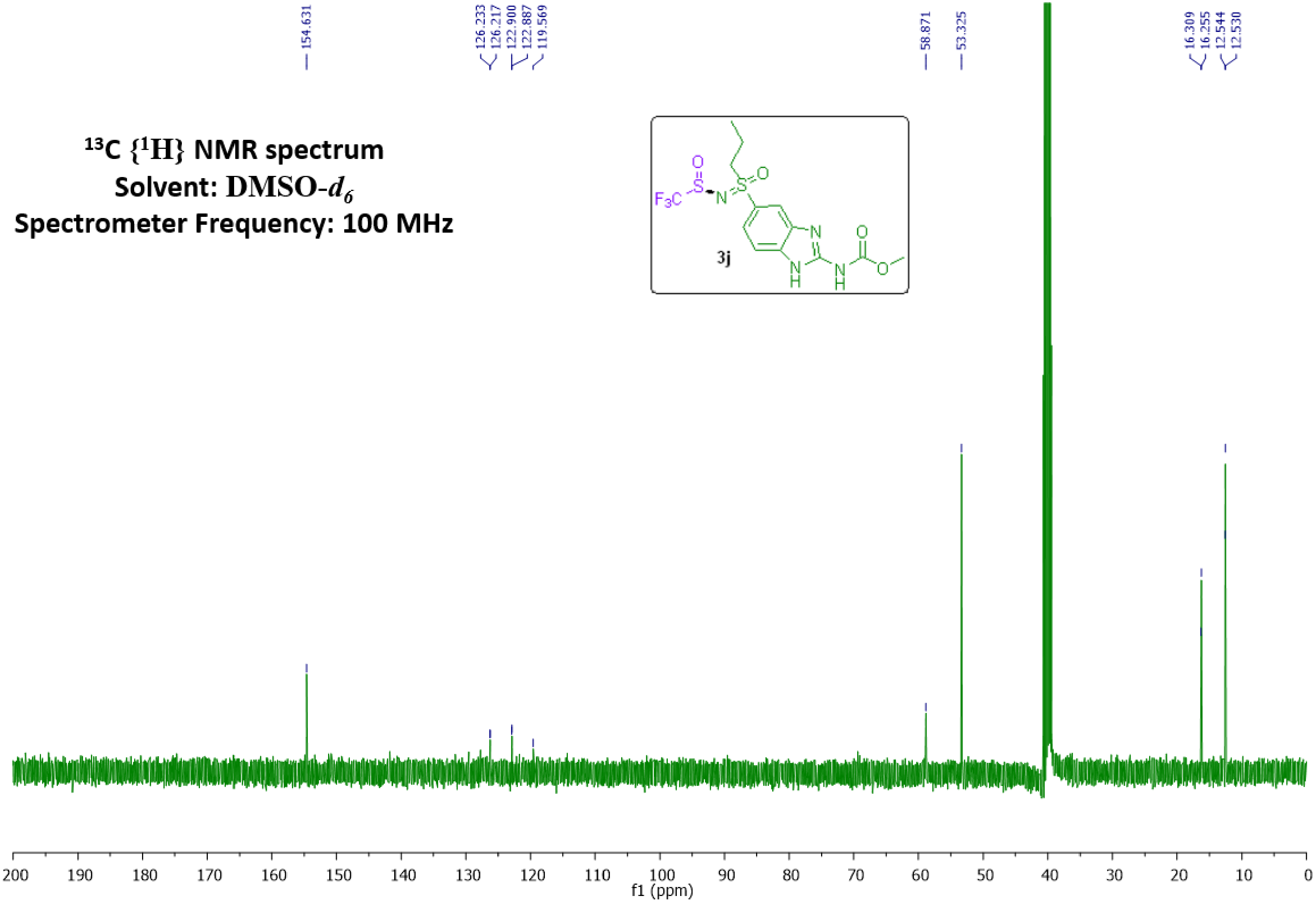

^{19}F {^{1}H} NMR spectrum
Solvent: DMSO-d_6
Spectrometer Frequency: 376 MHz

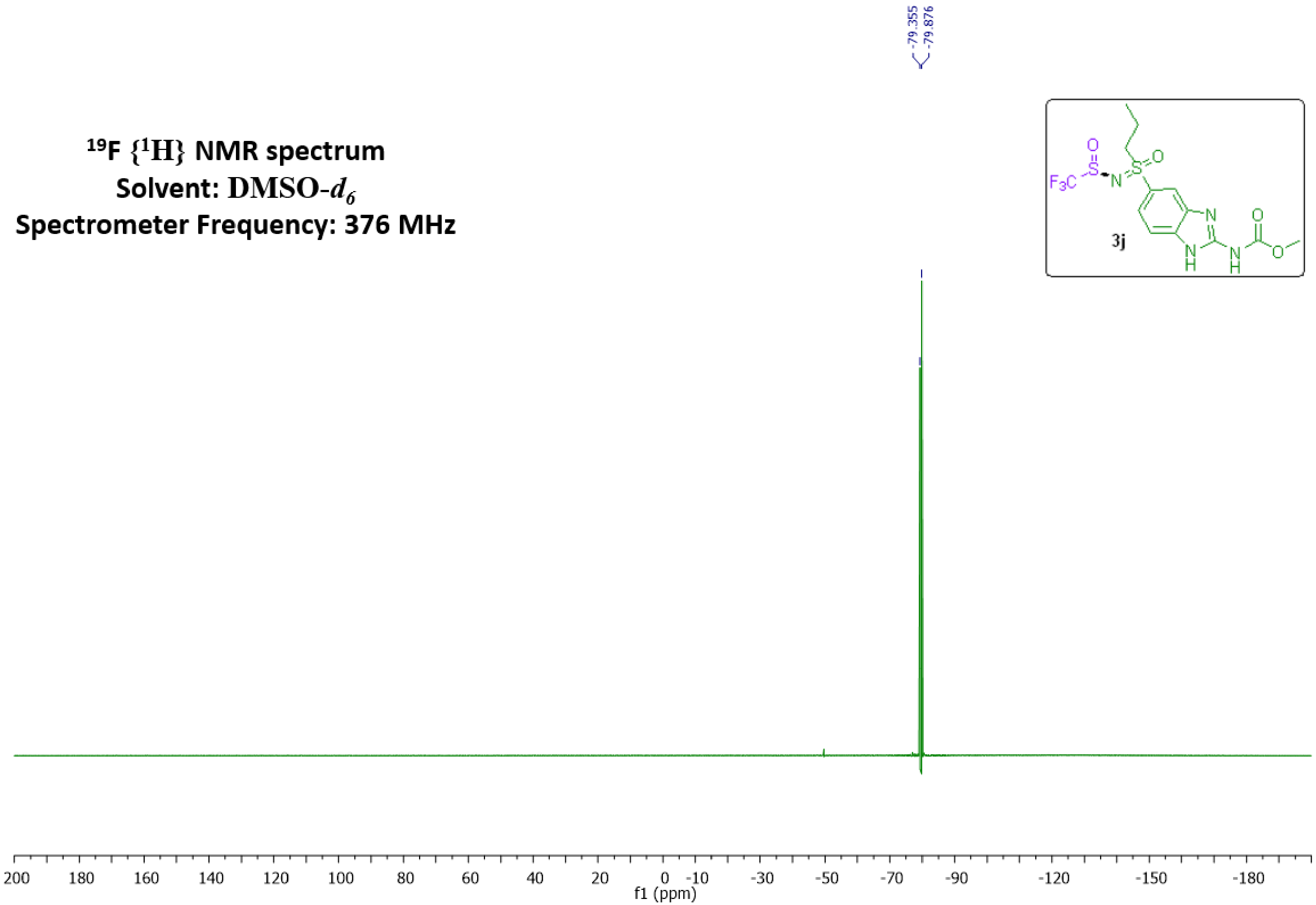

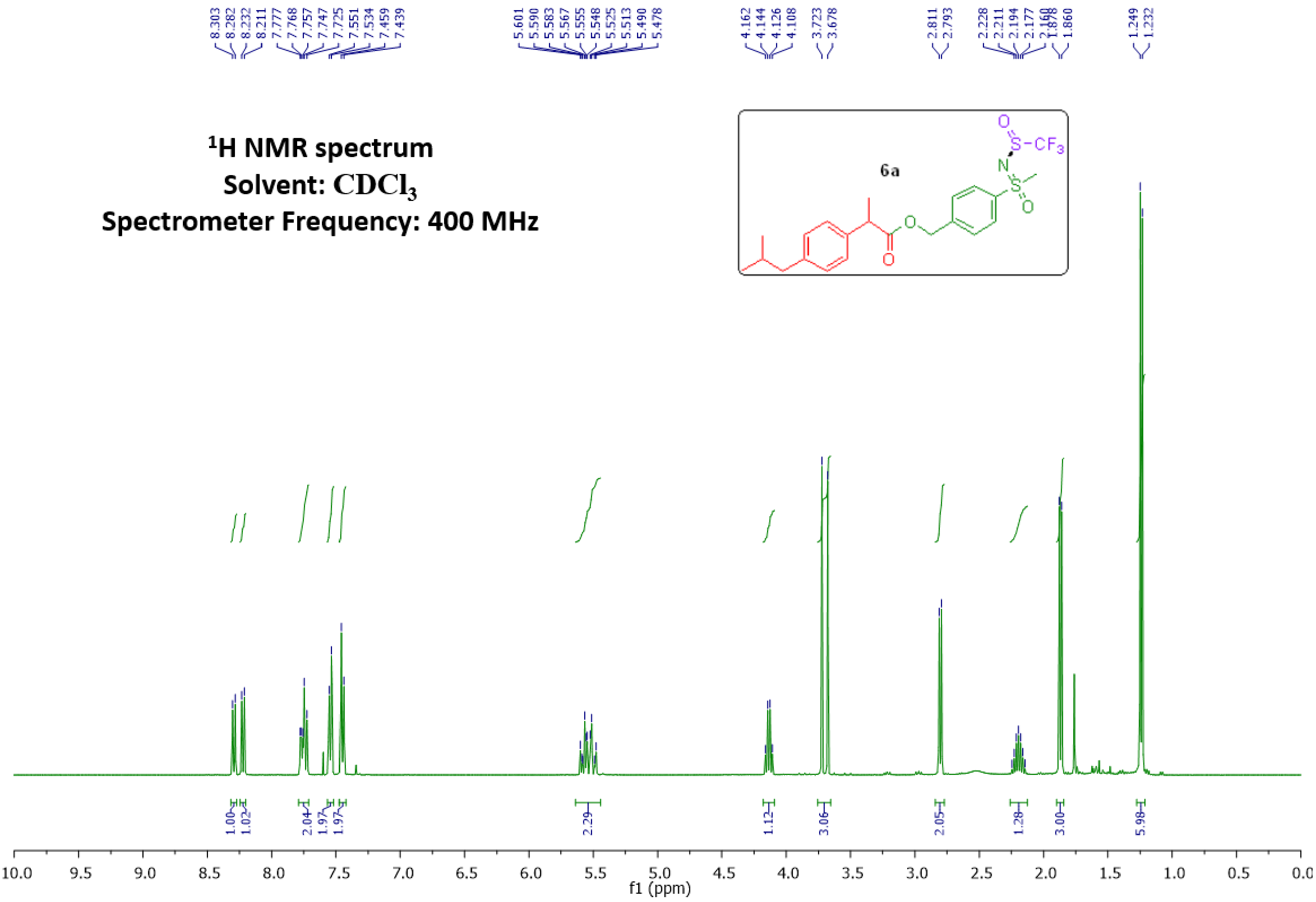

1H NMR spectrum
Solvent: CDCl3
Spectrometer Frequency: 400 MHz
6a
8.303
8.282
8.232
8.211
7.777
7.768
7.757
7.747
7.725
7.551
7.534
7.459
7.439
5.601
5.590
5.583
5.567
5.555
5.548
5.525
5.513
5.490
5.478
4.162
4.144
4.126
4.108
3.723
3.678
2.811
2.793
2.228
2.211
2.194
2.177
1.878
1.860
1.249
1.232
1.00
1.02
2.04
1.97
1.97
2.29
1.12
3.06
2.05
1.28
3.00
5.98
f1 (ppm)

^{13}C {^{1}H} NMR spectrum
Solvent: $CDCl_3$
Spectrometer Frequency: 100 MHz

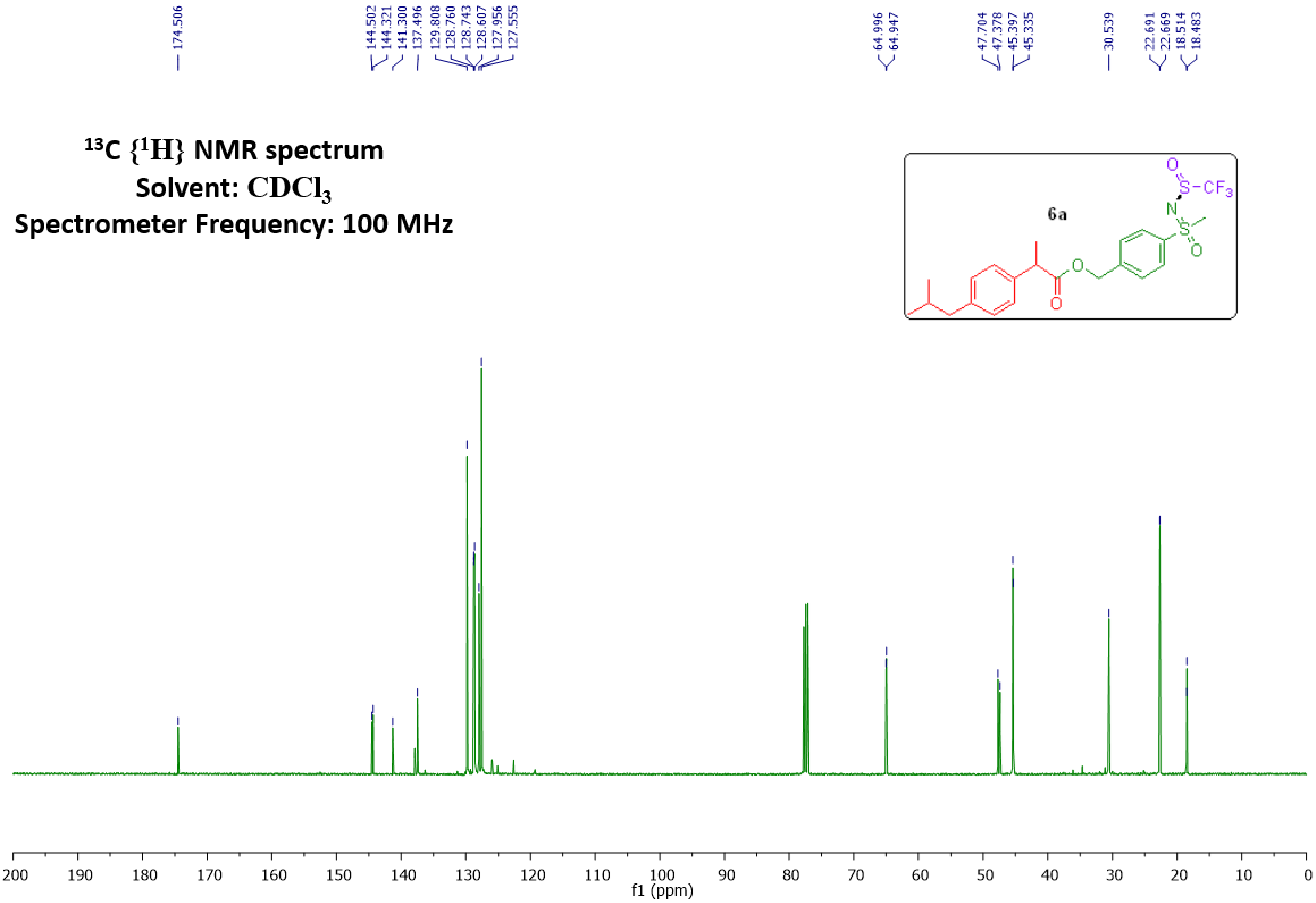

$^{19}F\ \{^{1}H\}$ NMR spectrum

Solvent: $CDCl_3$

Spectrometer Frequency: 376 MHz

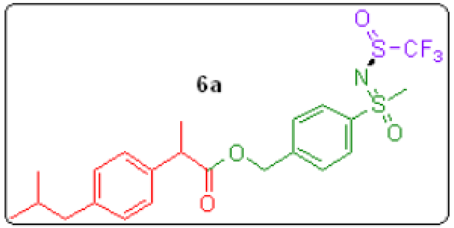

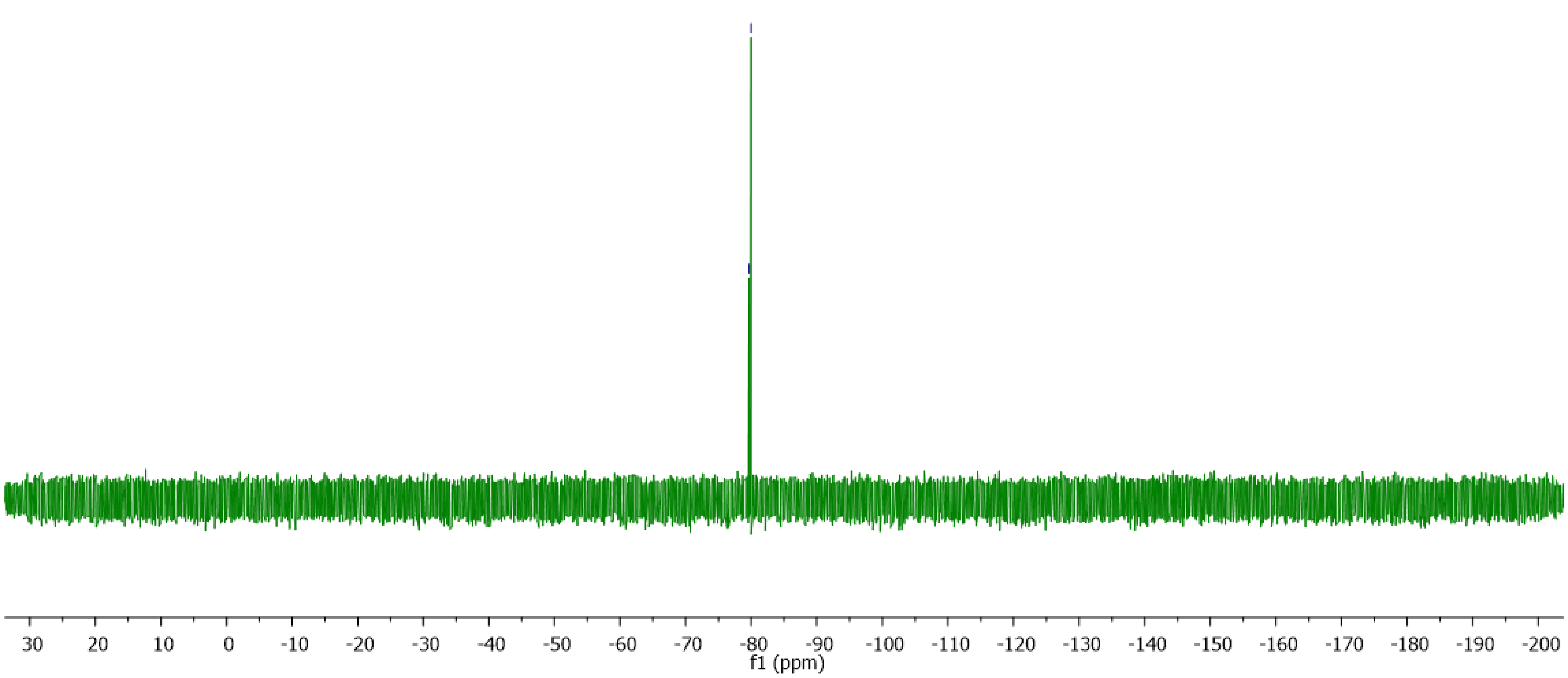

^{1}H NMR spectrum
Solvent: $CDCl_3$
Spectrometer Frequency: 400 MHz

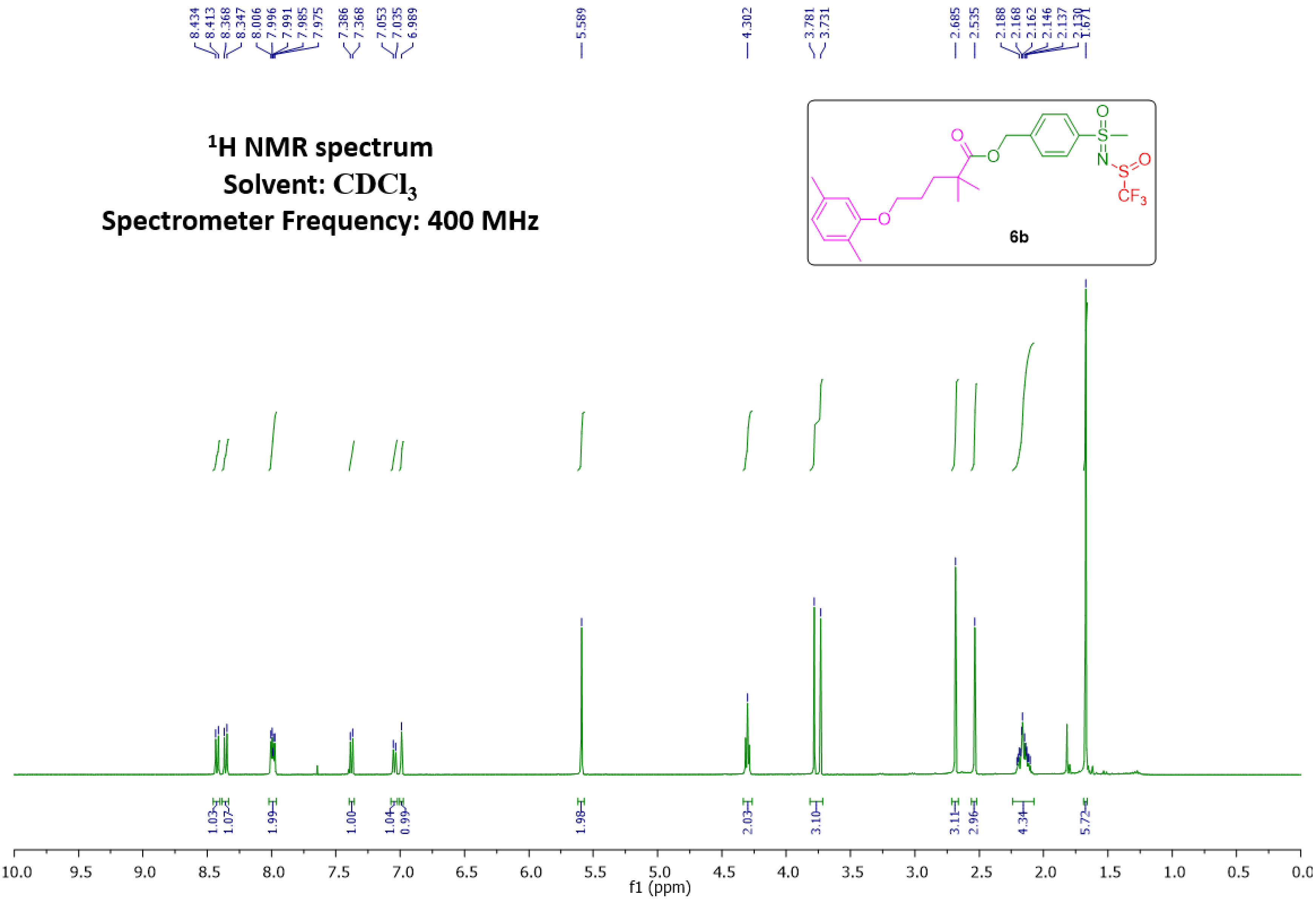

^{13}C {^{1}H} NMR spectrum
Solvent: $CDCl_3$
Spectrometer Frequency: 100 MHz

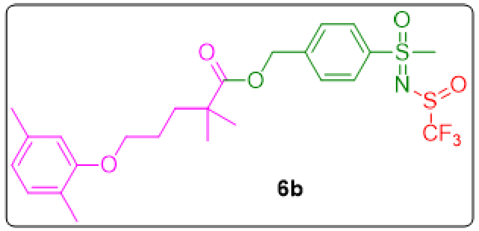

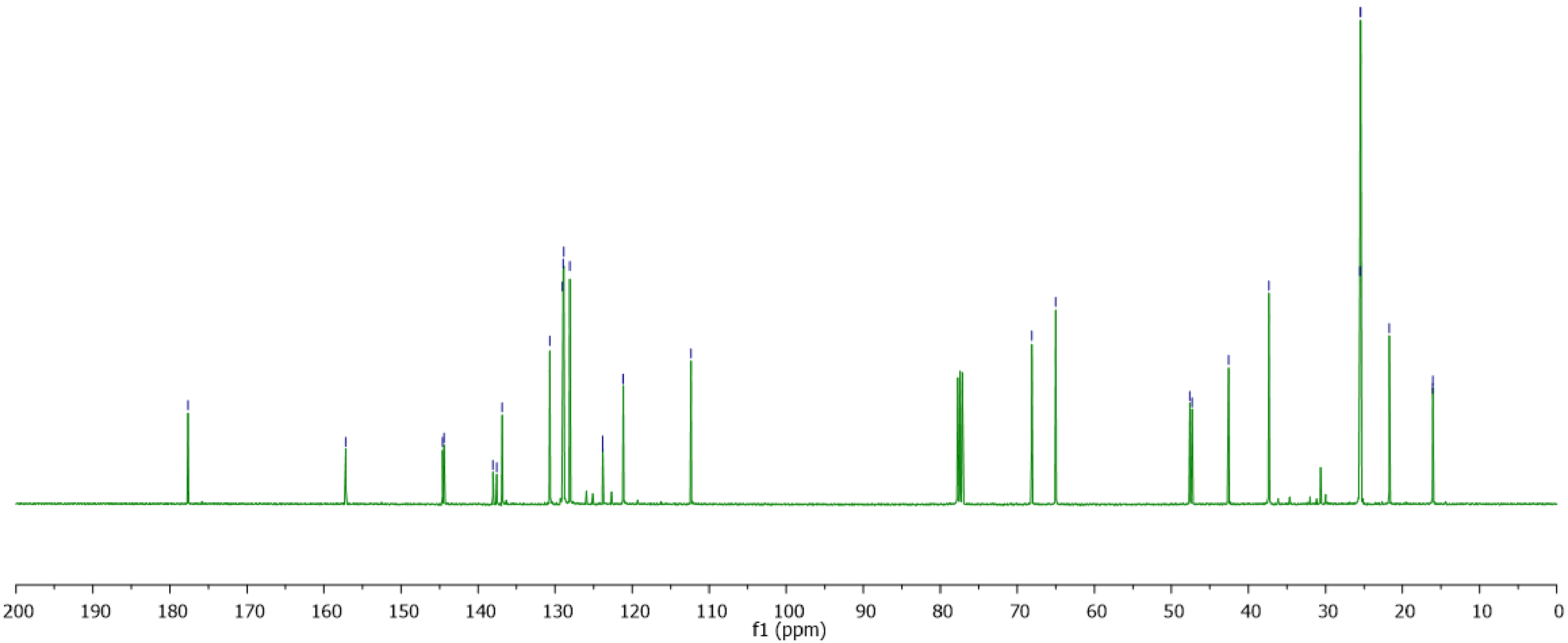

^{19}F {^{1}H} NMR spectrum
Solvent: $CDCl_3$
Spectrometer Frequency: 376 MHz

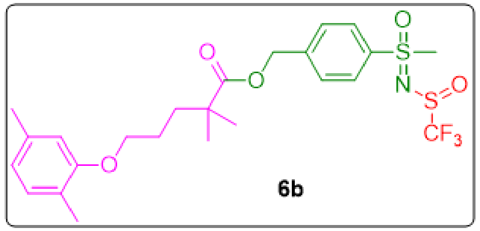

6b

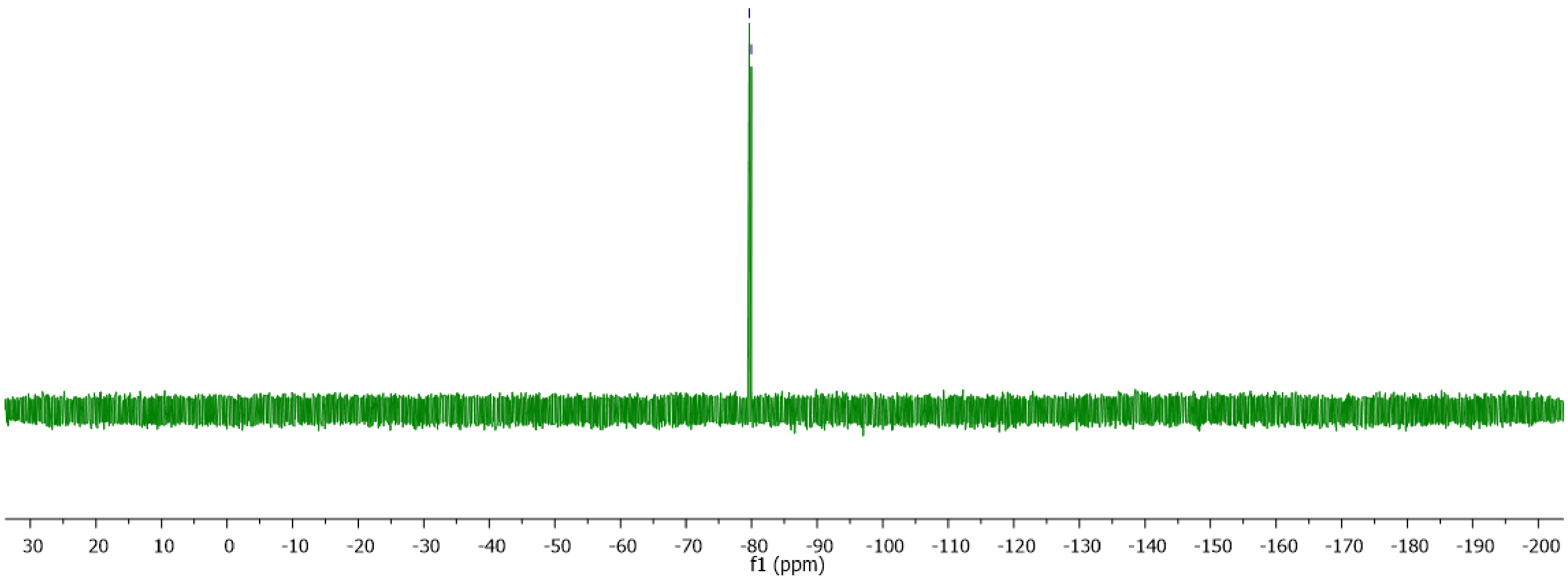

^{1}H NMR spectrum
Solvent: $CDCl_3$
Spectrometer Frequency: 400 MHz

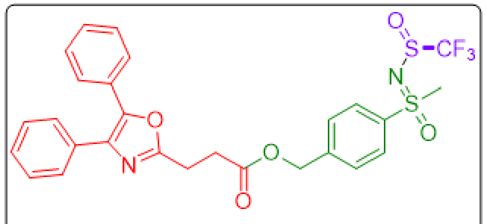

6c

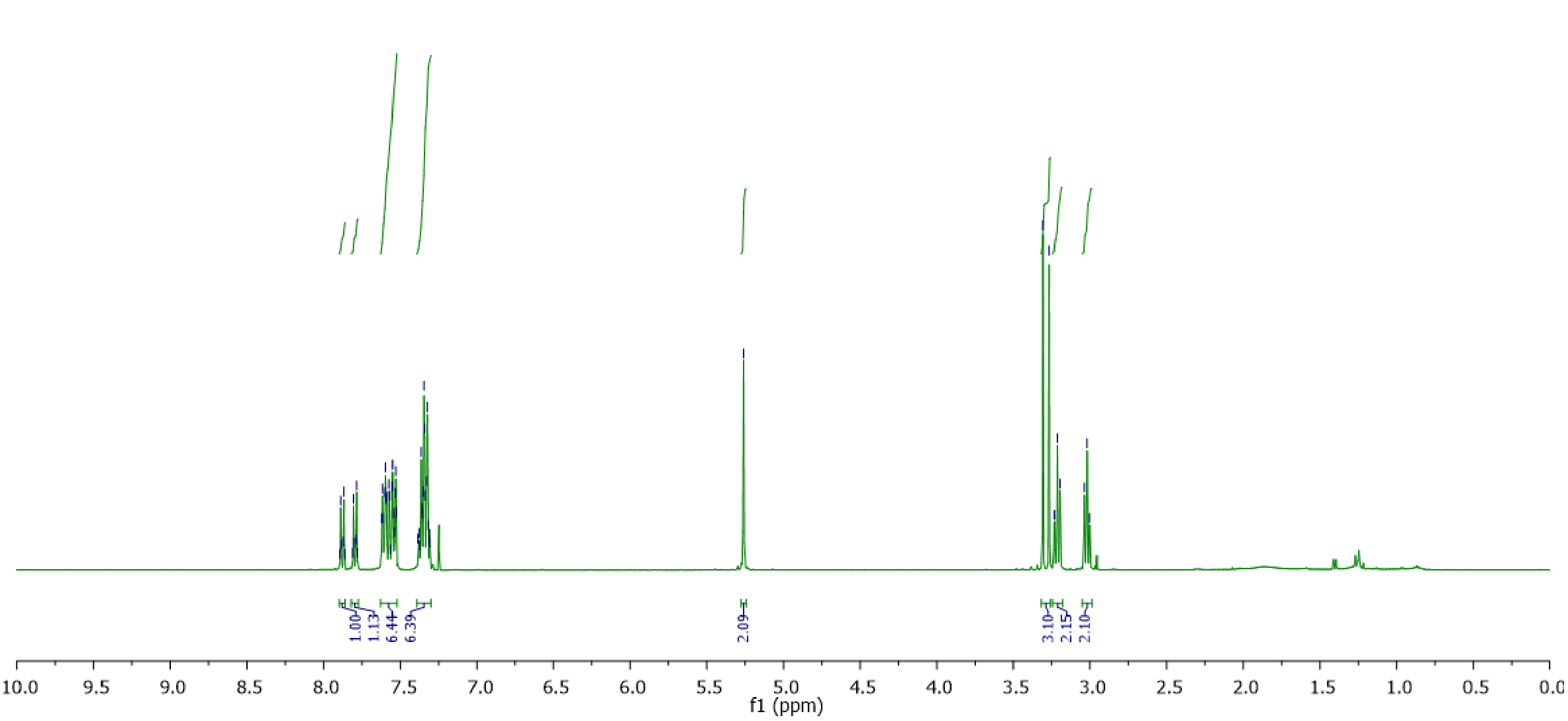

^{13}C {^{1}H} NMR spectrum
Solvent: $CDCl_3$
Spectrometer Frequency: 100 MHz

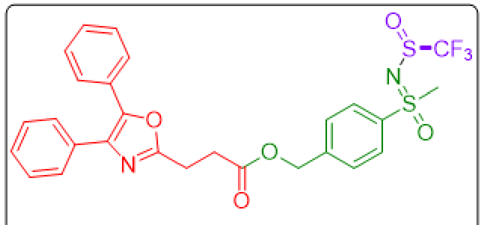

6c

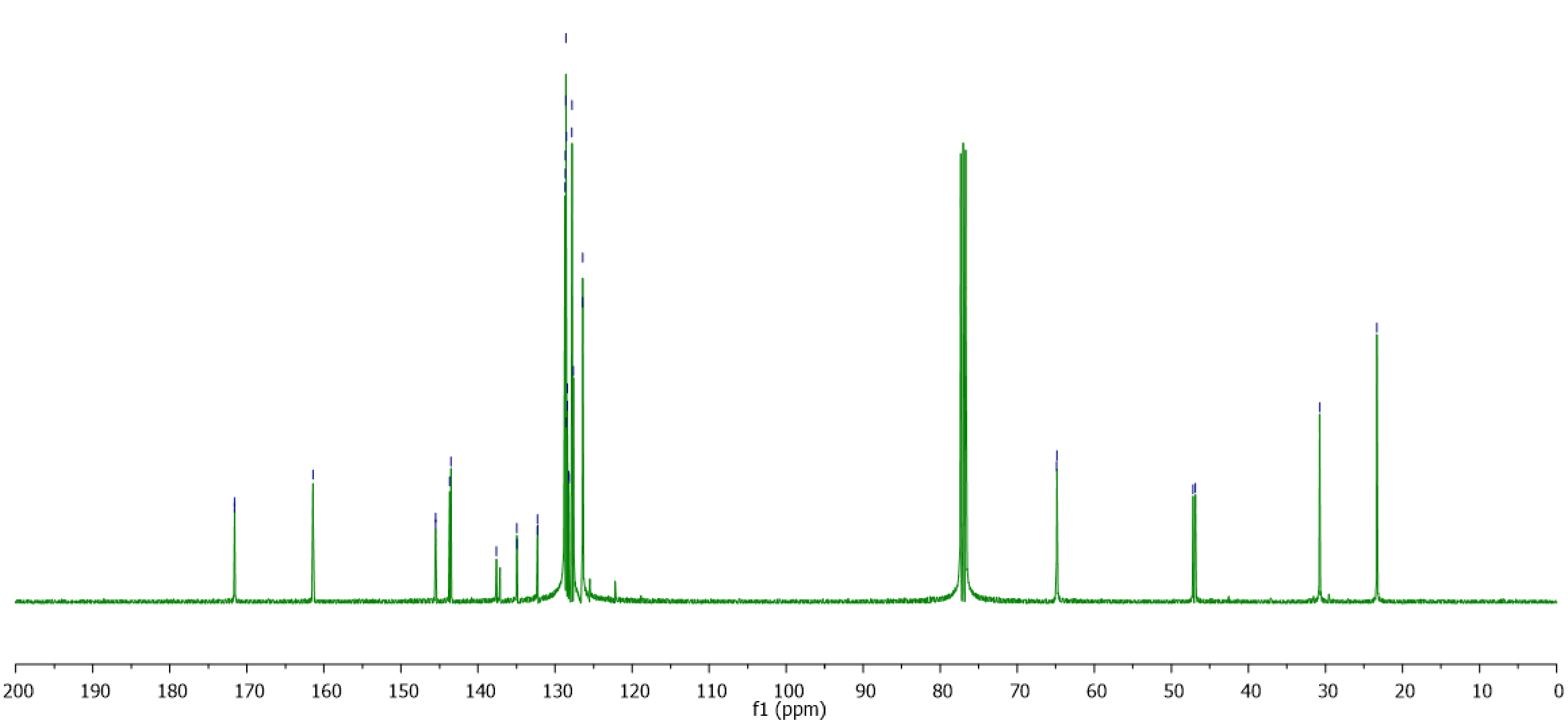

^{19}F {^{1}H} NMR spectrum
Solvent: $CDCl_3$
Spectrometer Frequency: 376 MHz

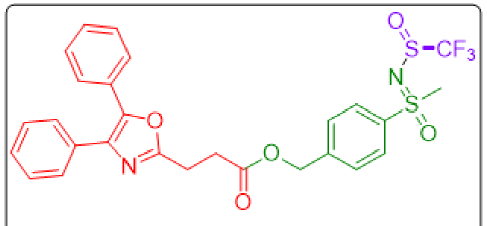

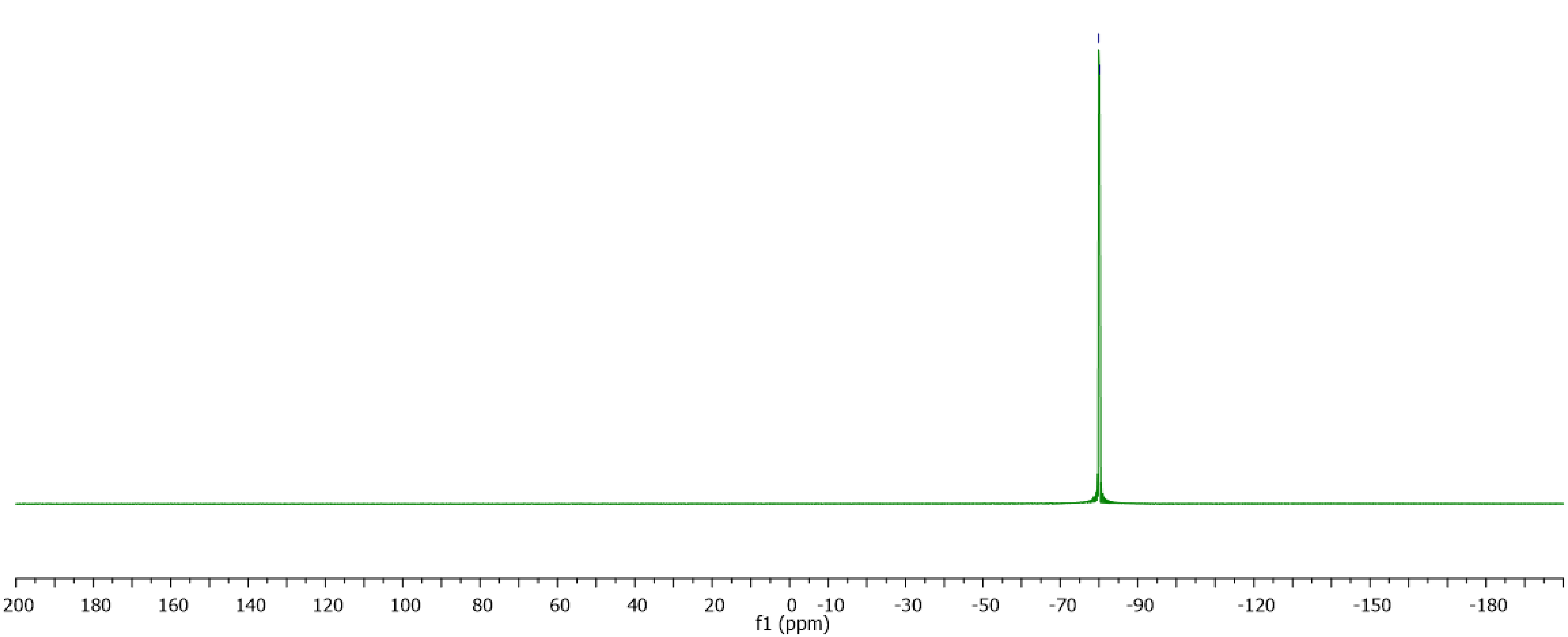

^{1}H NMR spectrum
Solvent: $CDCl_3$
Spectrometer Frequency: 400 MHz

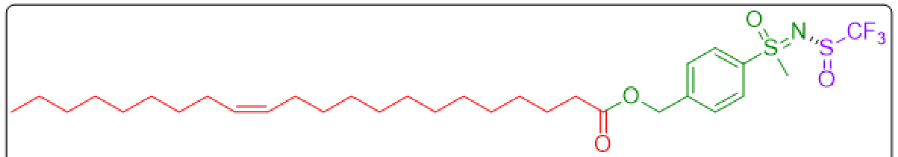
6d

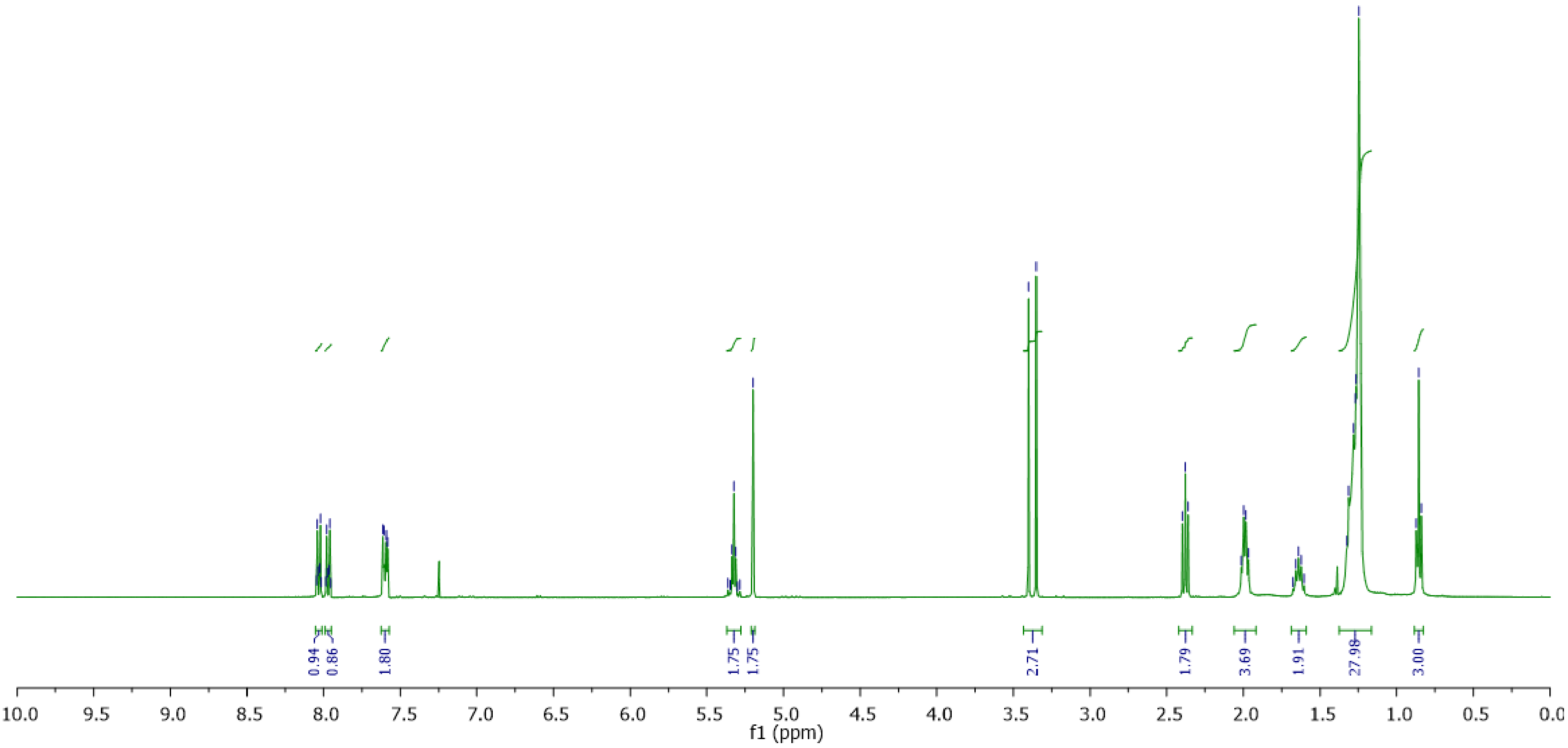

^{13}C {^{1}H} NMR spectrum
Solvent: $CDCl_3$
Spectrometer Frequency: 100 MHz

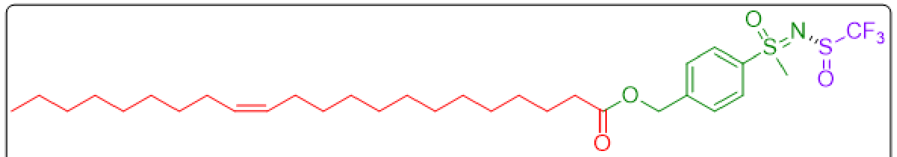

6d

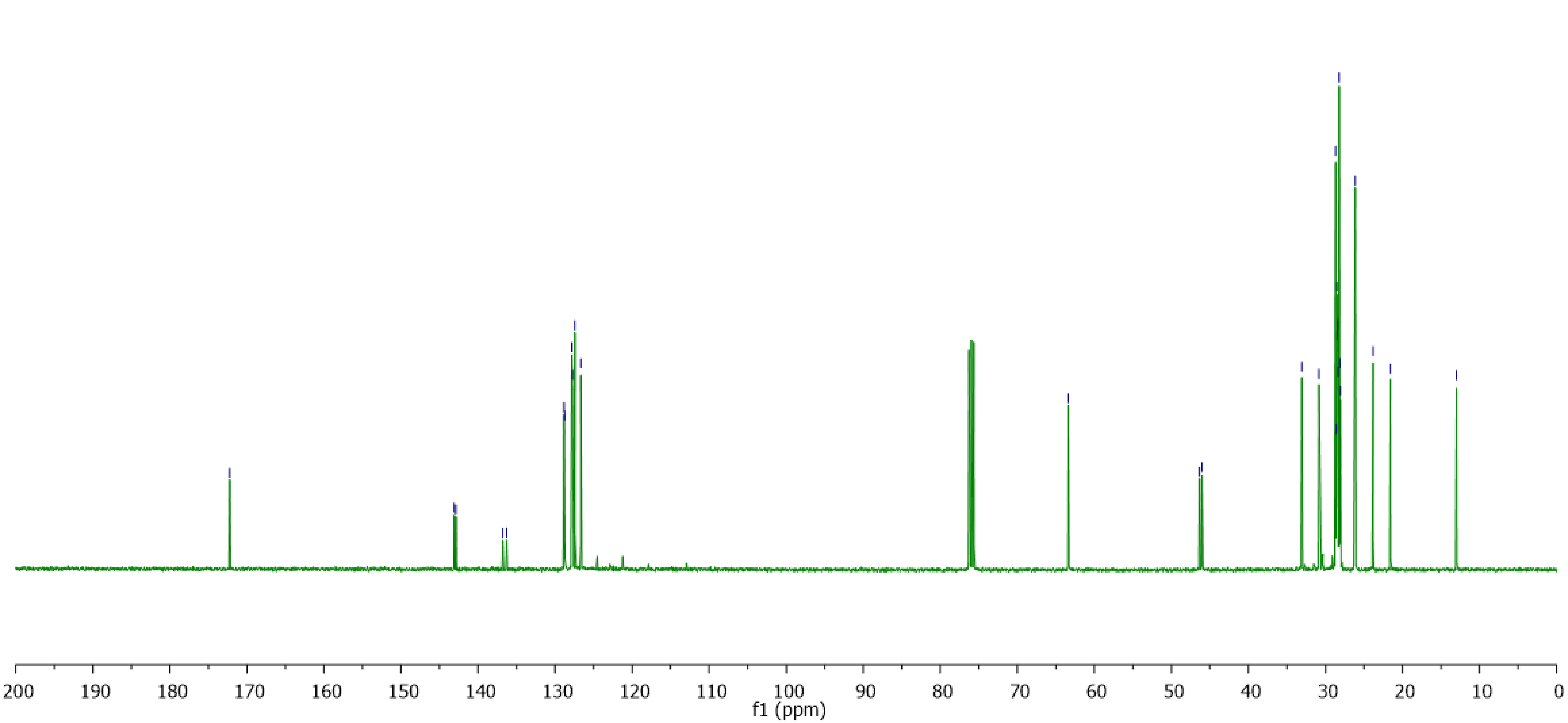

^{19}F {^{1}H} NMR spectrum
Solvent: $CDCl_3$
Spectrometer Frequency: 376 MHz

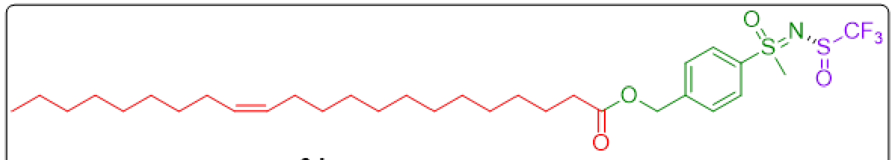

6d

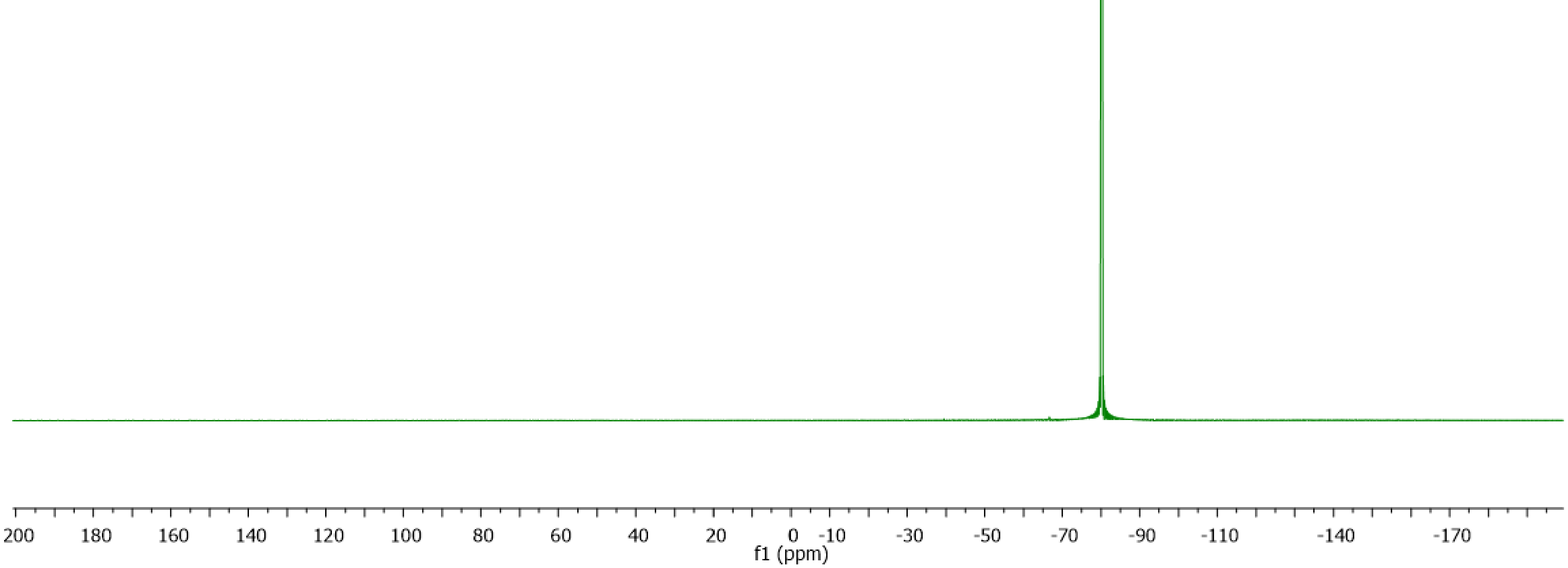

^{1}H NMR spectrum
Solvent: $CDCl_3$
Spectrometer Frequency: 400 MHz

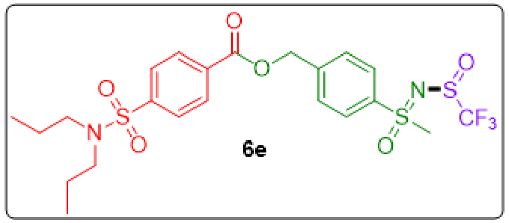

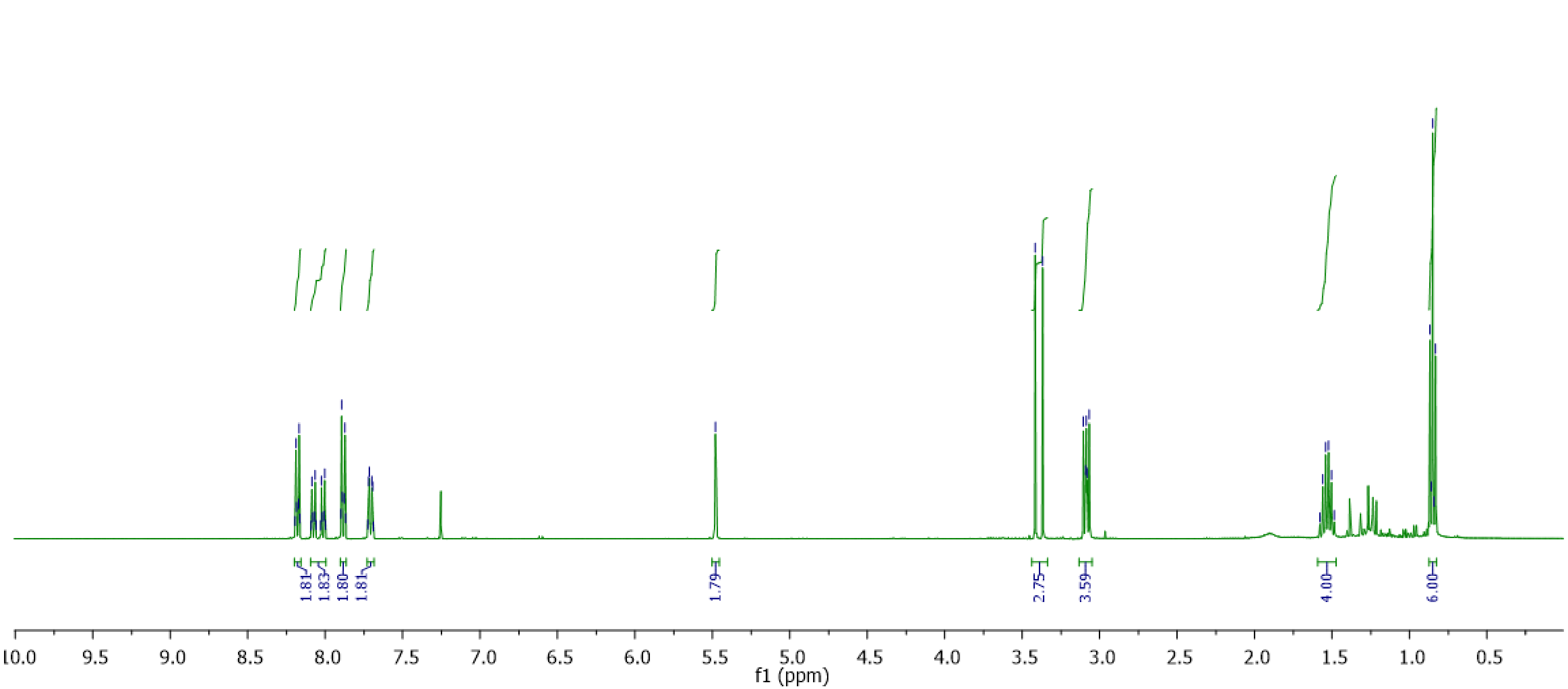

^{13}C {^{1}H} NMR spectrum
Solvent: $CDCl_3$
Spectrometer Frequency: 100 MHz

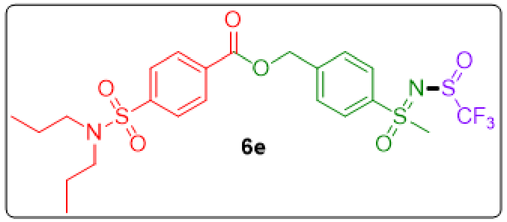

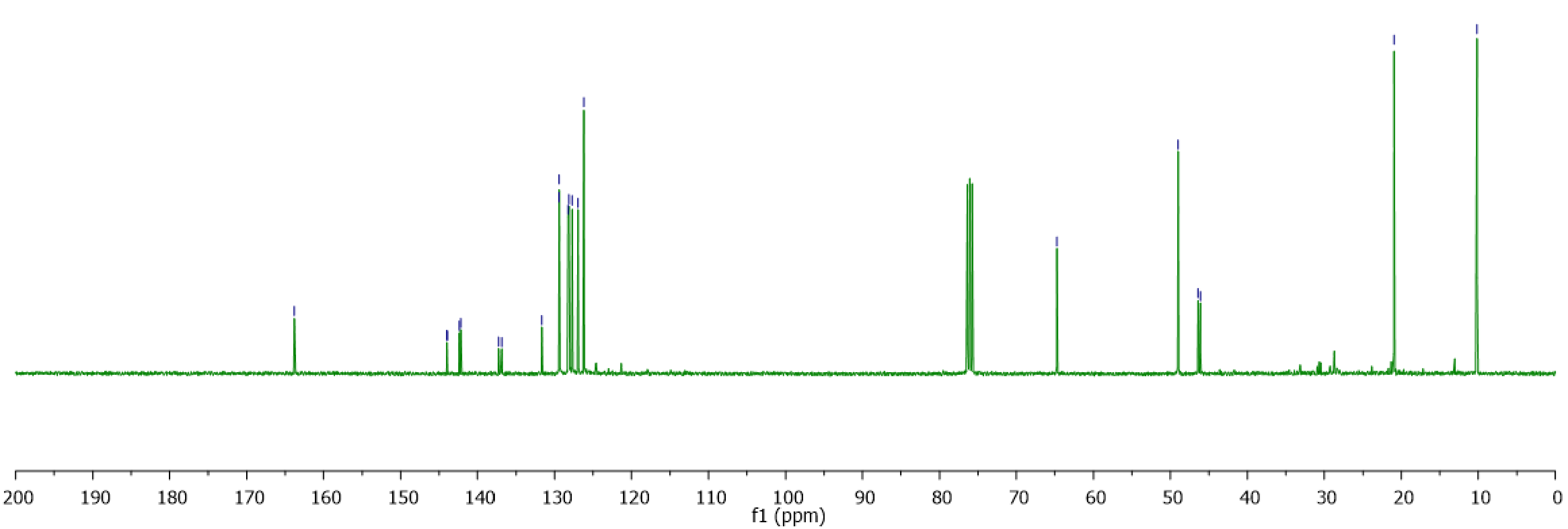

^{19}F {^{1}H} NMR spectrum
Solvent: $CDCl_3$
Spectrometer Frequency: 376 MHz

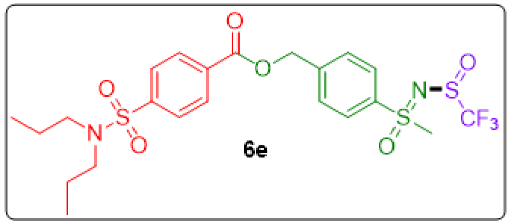

6e

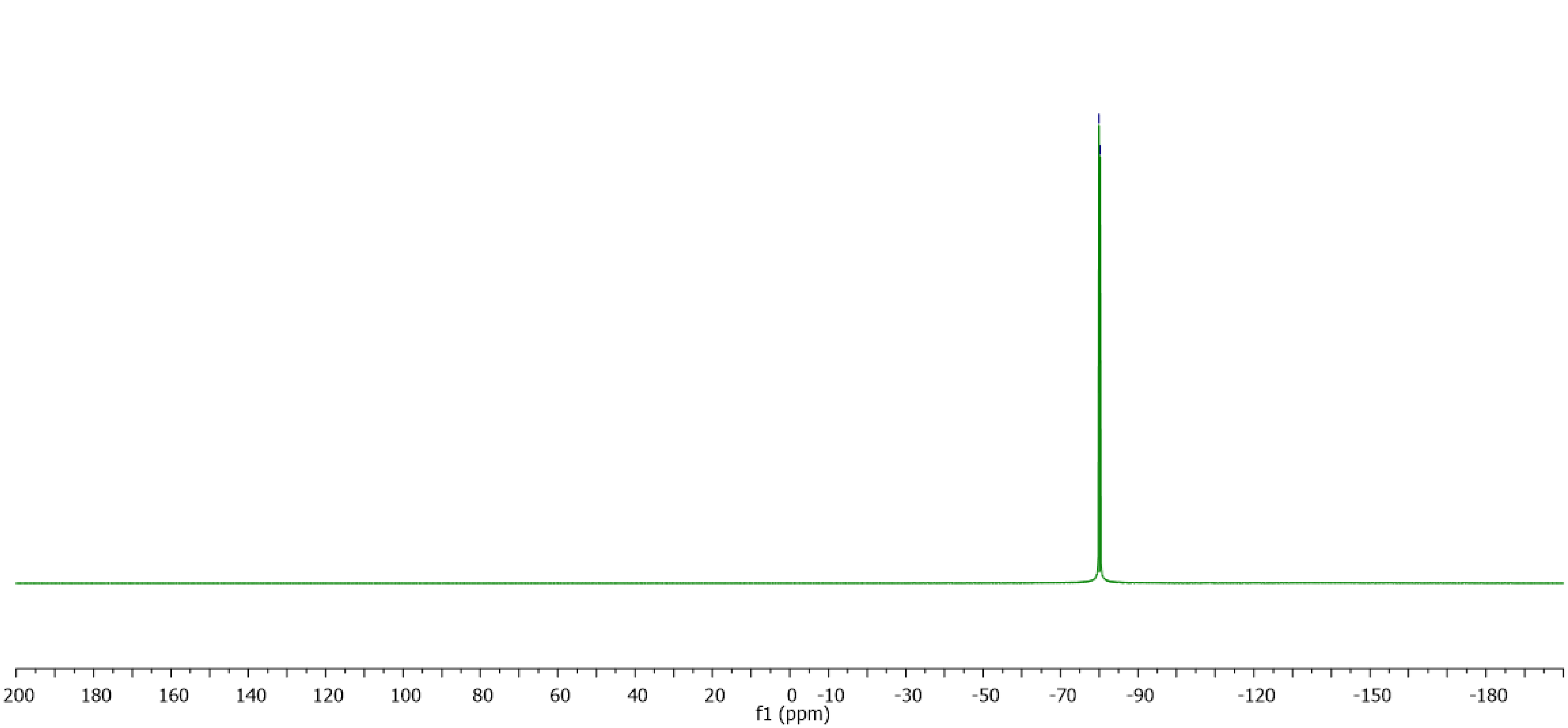

^1H NMR spectrum

Solvent: $CDCl_3$

Spectrometer Frequency: 400 MHz

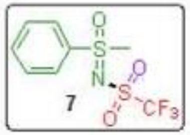

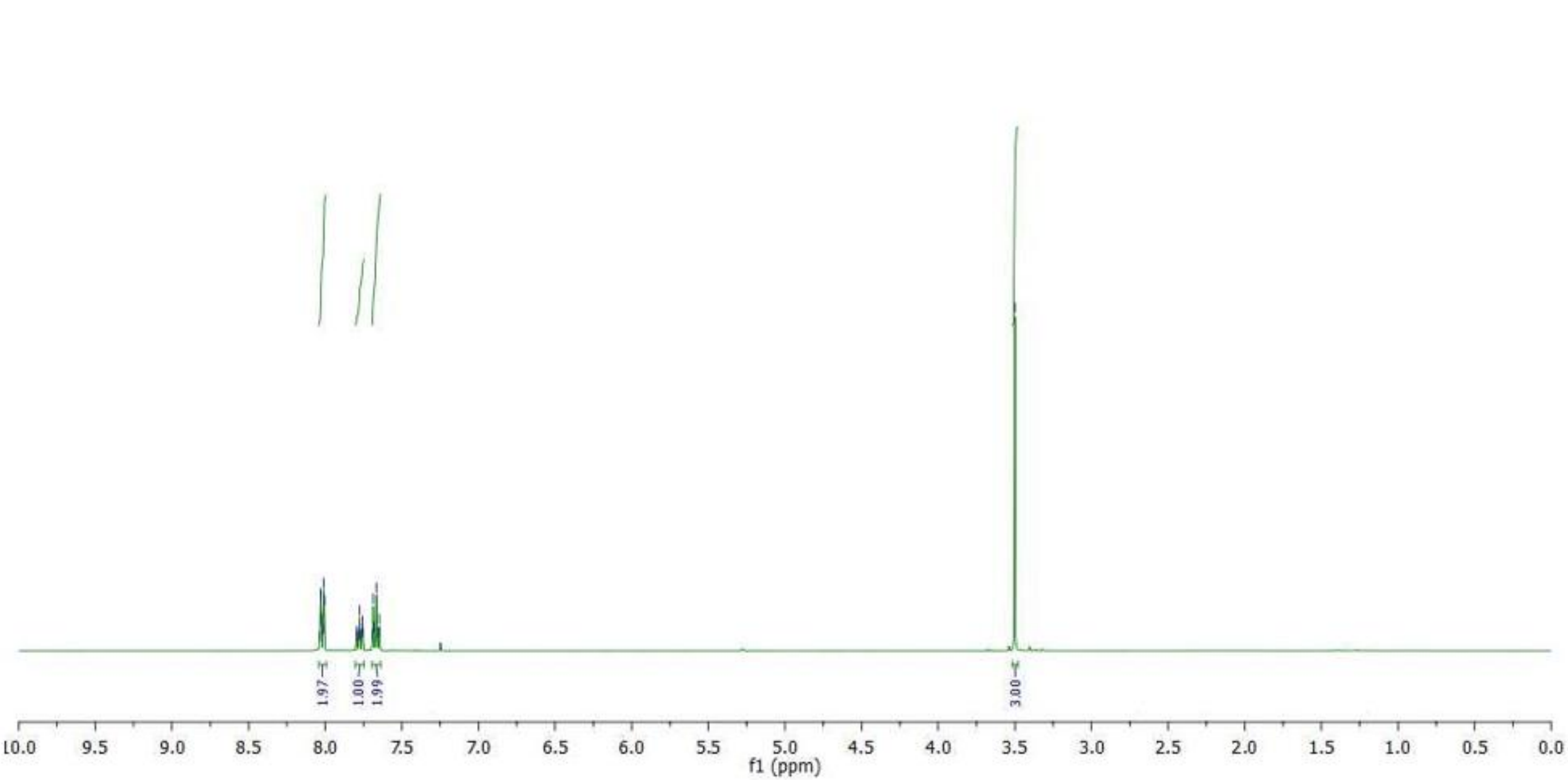

^{13}C {^1H} NMR spectrum
Solvent: $CDCl_3$
Spectrometer Frequency: 100 MHz

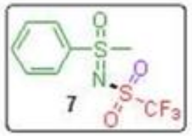

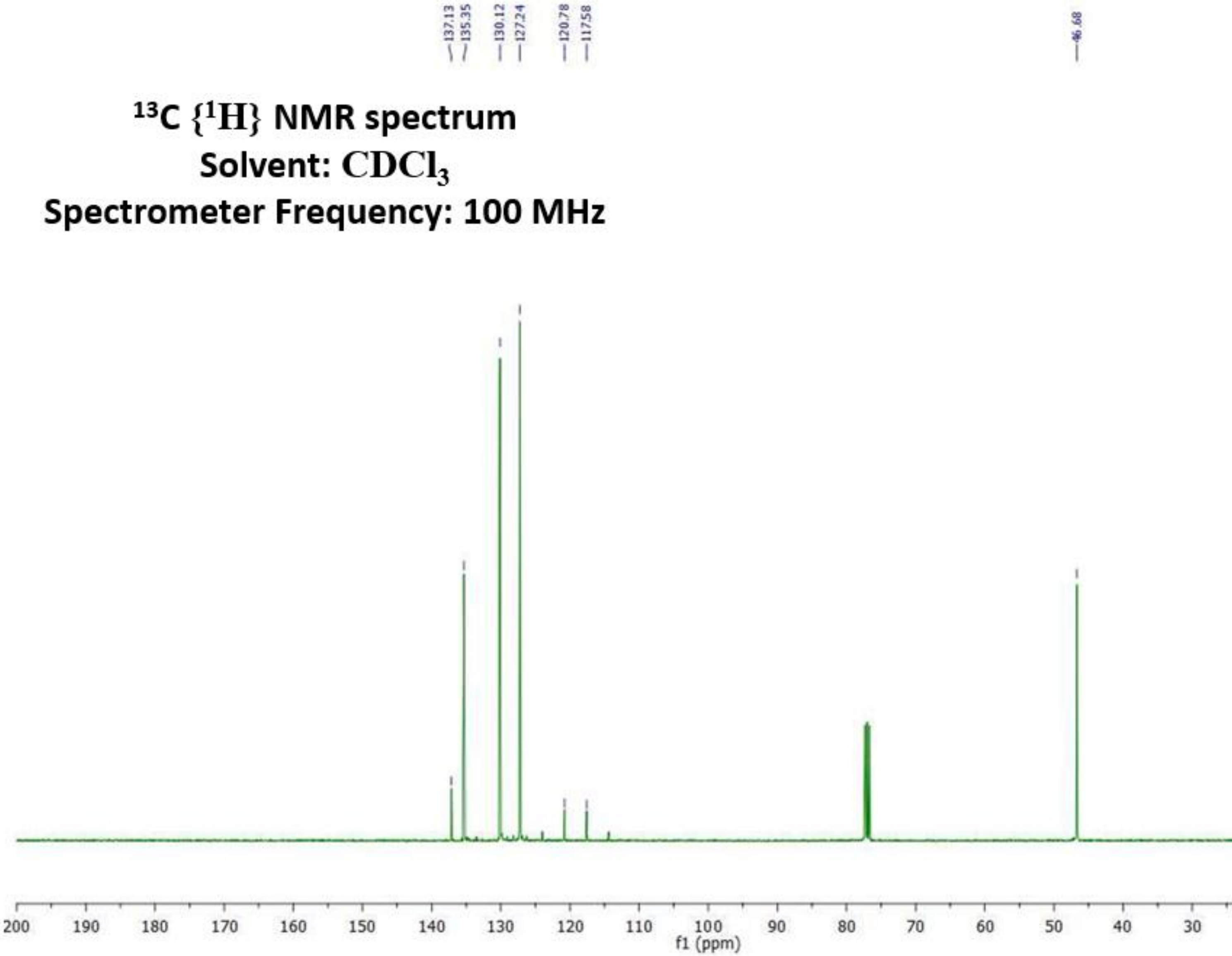

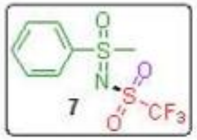

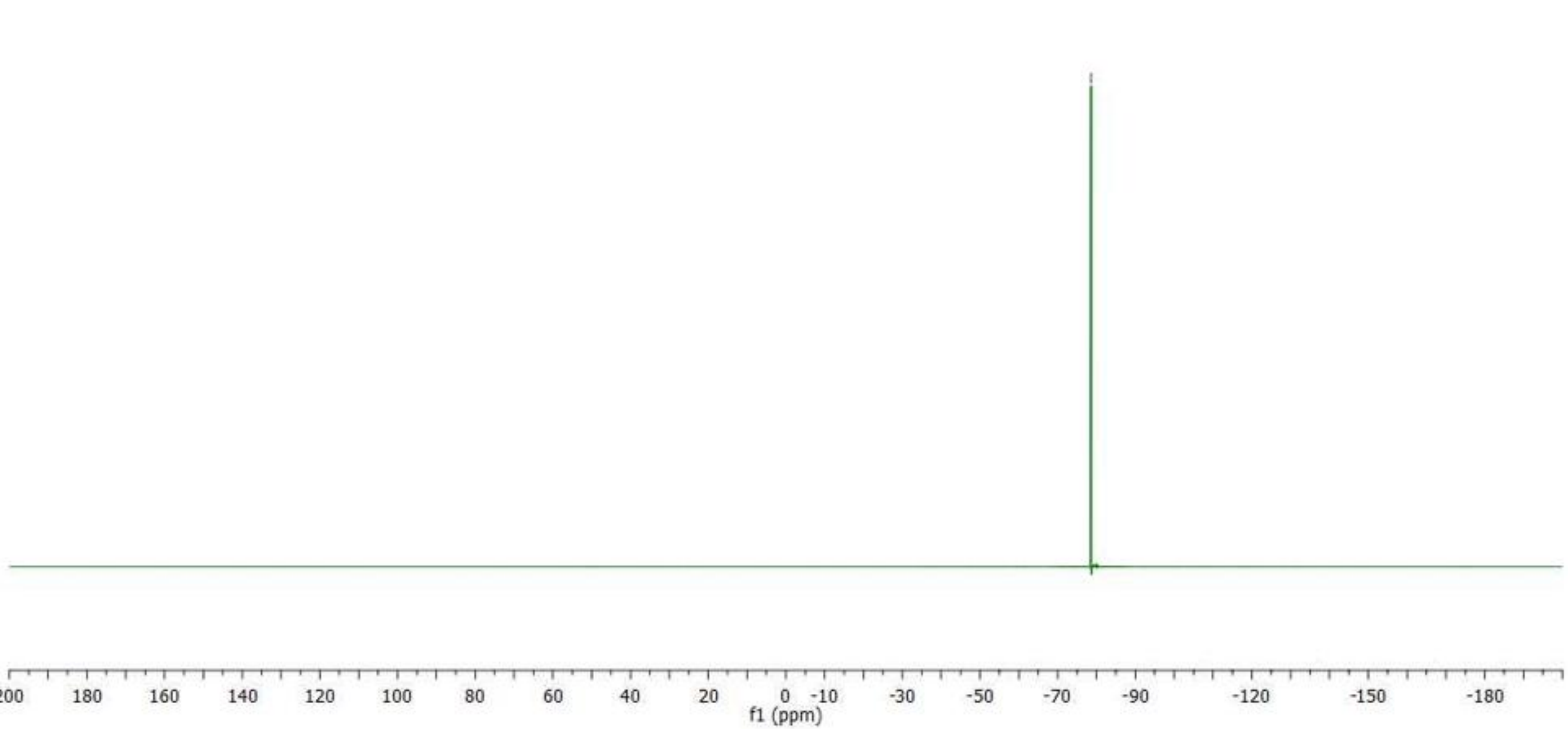

$^{19}F\ \{^{1}H\}$ NMR spectrum
Solvent: $CDCl_3$
Spectrometer Frequency: 376 MHz

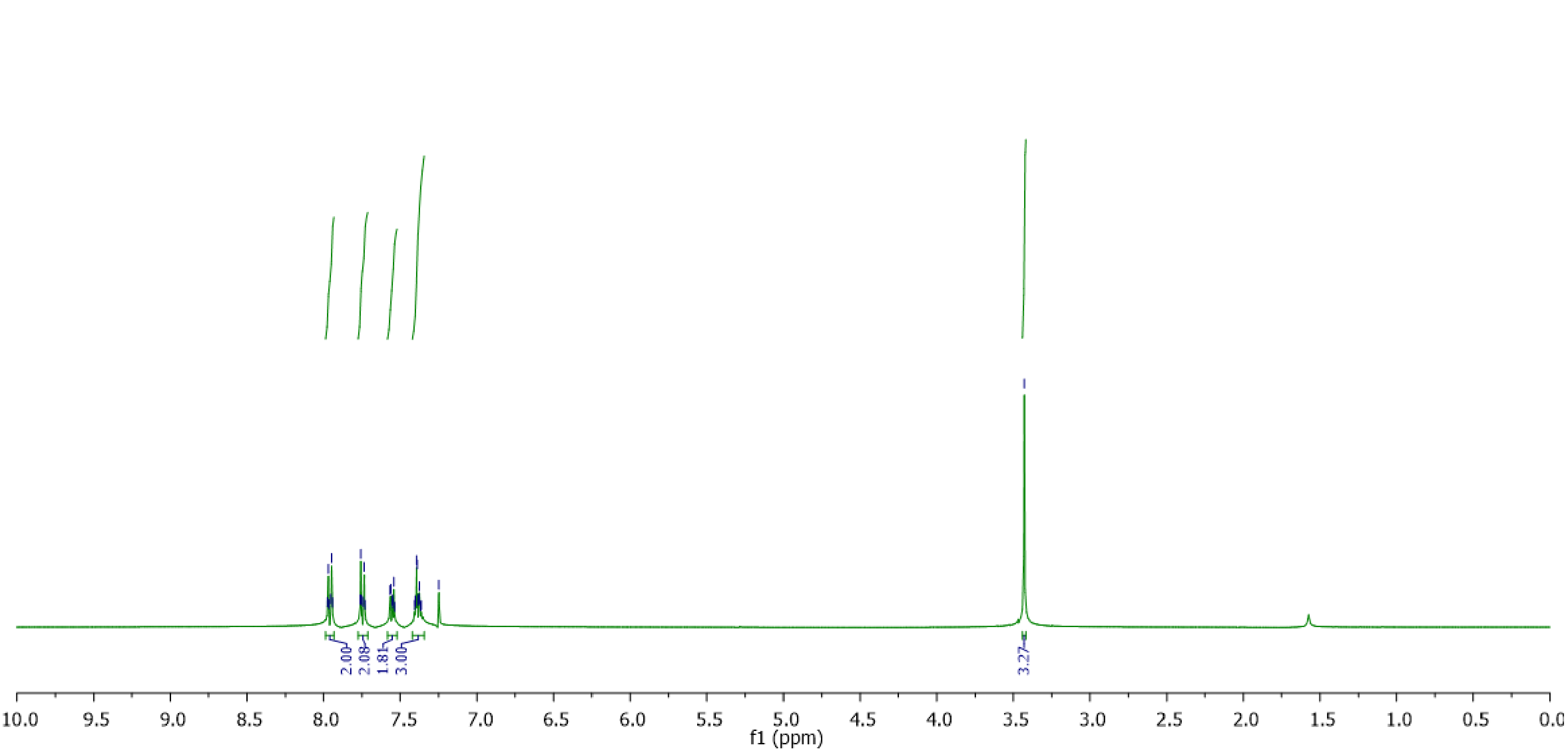

^{1}H NMR spectrum
Solvent: $CDCl_3$
Spectrometer Frequency: 400 MHz

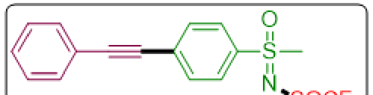

9

^{13}C {^{1}H} NMR spectrum
Solvent: $CDCl_3$
Spectrometer Frequency: 100 MHz

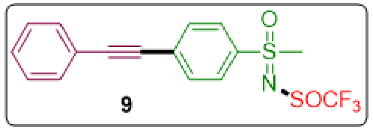

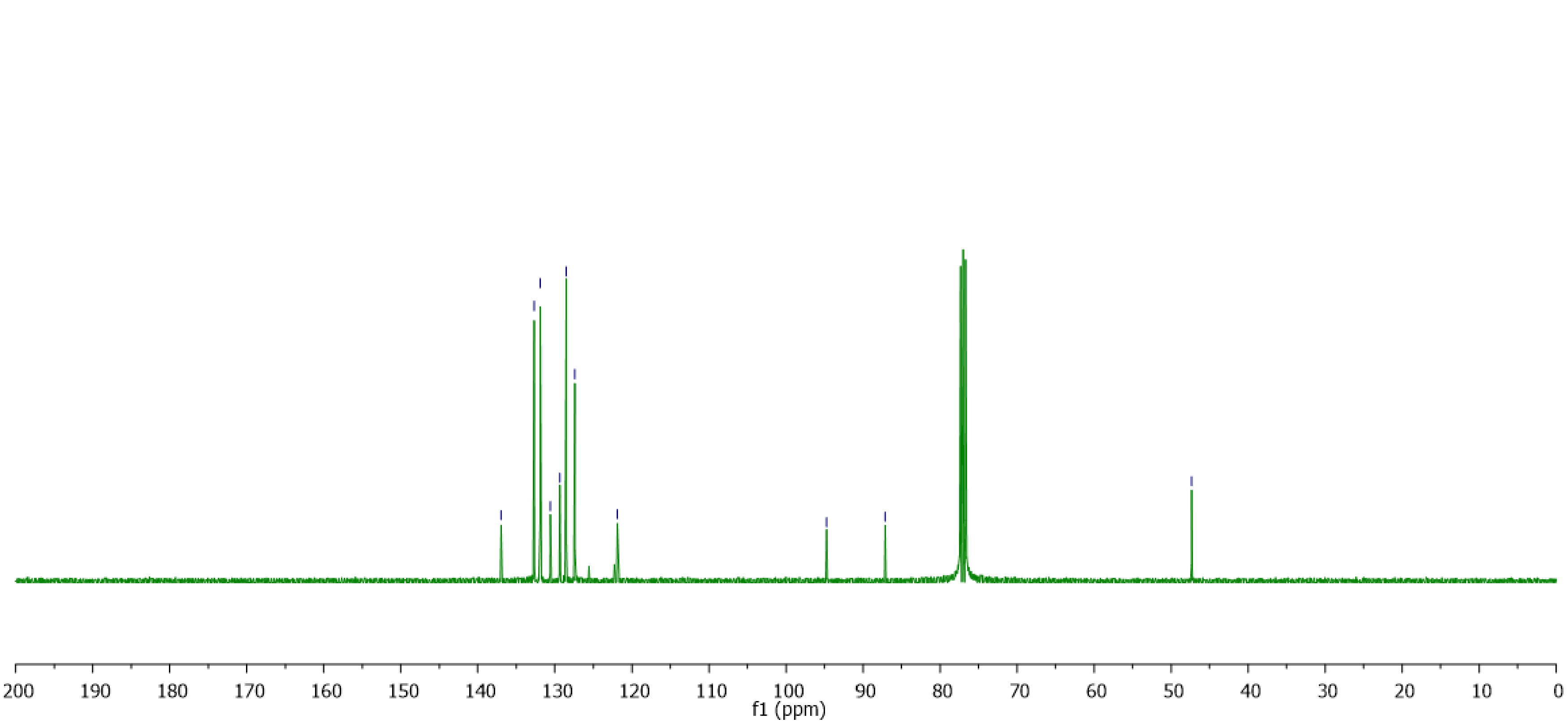

^{19}F {^{1}H} NMR spectrum
Solvent: $CDCl_3$
Spectrometer Frequency: 376 MHz

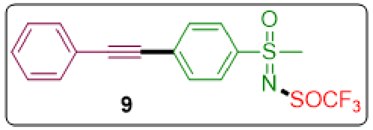

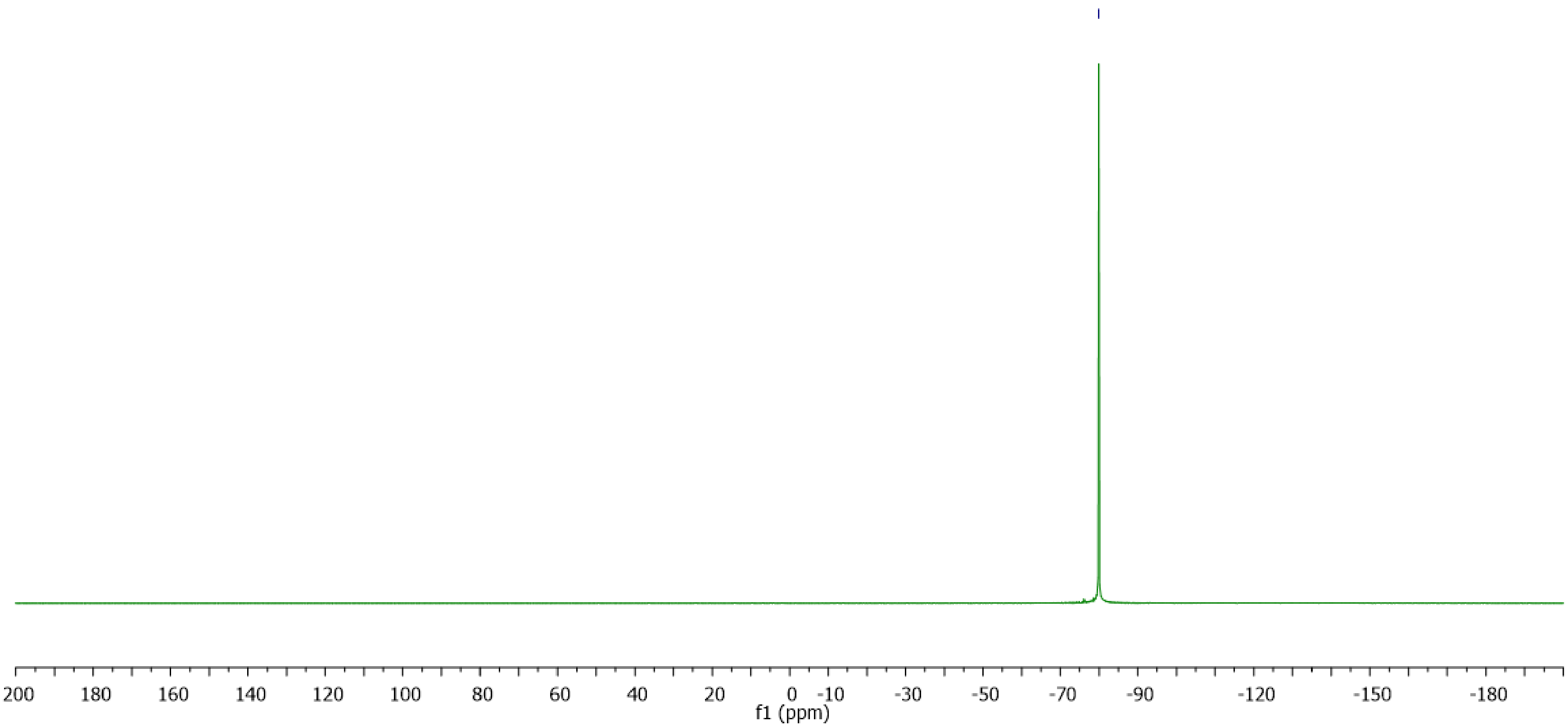

Supplement: Supplementary file 1 [file jo6c00569_si_001.pdf]
